# Direct insights into the role of epoxy groups on cobalt sites for acidic H_2_O_2_ production

**DOI:** 10.1038/s41467-020-17782-5

**Published:** 2020-08-21

**Authors:** Qingran Zhang, Xin Tan, Nicholas M. Bedford, Zhaojun Han, Lars Thomsen, Sean Smith, Rose Amal, Xunyu Lu

**Affiliations:** 1 Particles and Catalysis Research Group, School of Chemical Engineering, The University of New South Wales, Sydney, NSW 2052 Australia; 2 Integrated Materials Design Laboratory, Department of Applied Mathematics, Research School of Physics and Engineering, The Australian National University, Canberra, ACT 2601 Australia; 3 School of Mechanical and Manufacturing Engineering, The University of New South Wales, Sydney, NSW 2052 Australia; 4 CSIRO Manufacturing, P.O. Box 218, 36 Bradfield Road, Lindfield, NSW 2070 Australia; 5 Australian Synchrotron, Australian Nuclear Science and Technology Organisation, 800 Blackburn Road, Clayton, VIC 3168 Australia

**Keywords:** Electrocatalysis, Energy

## Abstract

Hydrogen peroxide produced by electrochemical oxygen reduction reaction provides a potentially cost effective and energy efficient alternative to the industrial anthraquinone process. In this study, we demonstrate that by modulating the oxygen functional groups near the atomically dispersed cobalt sites with proper electrochemical/chemical treatments, a highly active and selective oxygen reduction process for hydrogen peroxide production can be obtained in acidic electrolyte, showing a negligible amount of onset overpotential and nearly 100% selectivity within a wide range of applied potentials. Combined spectroscopic results reveal that the exceptionally enhanced performance of hydrogen peroxide generation originates from the presence of epoxy groups near the Co–N_4_ centers, which has resulted in the modification of the electronic structure of the cobalt atoms. Computational modeling demonstrates these electronically modified cobalt atoms will enhance the hydrogen peroxide productivity during oxygen reduction reaction in acid, providing insights into the design of electroactive materials for effective peroxide production.

## Abstract (web summary)

The production of hydrogen peroxide by electrochemical oxygen reduction is an attractive alternative to the industrial process, but catalysts should be optimized. Here, the authors enhance hydrogen peroxide production in acidic media with epoxy groups near cobalt centers on carbon nanotubes.

## Introduction

Hydrogen peroxide (H_2_O_2_) is an important chemical commodity that has been widely used as an environmentally benign oxidant and a potential energy carrier in various applications, including wastewater treatment^1^, disinfection, chemical synthesis^2,3^, paper/pulp bleaching, semiconductor cleaning^4^, and fuel cells^5^. The global demand of H_2_O_2_ is growing rapidly, reaching US$4.0 billion in 2017 and is expected to further increase to around US$5.5 billion by 2023^6^. Currently, over 95% of H_2_O_2_ is produced in a concentrated form using the anthraquinone process. This centralized production requires a huge infrastructure investment and high energy inputs, as well as poses safety concerns during the product distribution^7^. As a matter of fact, in many applications, only dilute H_2_O_2_ is required, indicating the concentrated peroxide agent needs to be diluted before usage. Moreover, the generation of organic byproducts and the hazard of handling the concentrated hydrogen peroxide make the anthraquinone method un-ecofriendly and uneconomic. Thus, it is highly desirable to develop green and cost-effective techniques for onsite H_2_O_2_ synthesis. Recently, the electrochemical reduction (ER) of oxygen via a selective two-electron transfer pathway has attracted extensive research interest as a promising alternative to the anthraquinone process. The oxygen reduction reaction (ORR) method will enable the decentralized production of H_2_O_2_ per demand under ambient reaction conditions without any hazardous byproducts^8,9^. Furthermore, if the ORR process is integrated with renewable electricity supplies (e.g., generation by photovoltaic cells or wind turbines), the H_2_O_2_ generated can be regarded as a renewable chemical^10^.

More recently, there has been a surge of interest in developing metal-free carbon catalysts for the electro-synthesis of H_2_O_2_ via O_2_ reduction^11,12^. The electronic structure of these carbon materials can be easily tuned by heteroatom-doping^13–15^ or defect-engineering^16,17^, thus endowing them high activity and selectivity towards H_2_O_2_ production in alkaline electrolytes. However, the production of H_2_O_2_ in alkaline media is somewhat constrained by the following limitations: (i) the H_2_O_2_ (or HO_2_^−^ at a pH value above 11.6) tends to readily decompose at basic conditions^18^; (ii) chelating agents (e.g., ethylenediamine tetraacetic acid) are required to prevent the tramp-metal-ion-induced H_2_O_2_ decomposition, thus resulting in the increased operation costs^19^; (iii) devices (such as fuel cell) based on hydroxide-conducting polymeric electrolytes exhibit low membrane stability, poor water management and low hydrogen oxidation activity^20,21^. Therefore, it is more desirable to generate H_2_O_2_ in acids via ORR. Currently, the state-of-the-art electrocatalysts for H_2_O_2_ production in acid are still restricted to the precious-metal-based (e.g., Pt–Hg and Pd–Hg) alloys or amalgams^22,23^, while their high cost and scarcity have severely hindered their commercial viability. Mesoporous carbon with nitrogen dopants^24^ or a defective structure^25^ has emerged as a type of promising catalysts for H_2_O_2_ electrosynthesis in acids, owing to their high H_2_O_2_ selectivity and low cost. Nevertheless, large amounts of overpotential (>400 mV) are normally associated with these materials to achieve high productivity and selectivity^26,27^, which has rendered the production process energy inefficient. Hence, the development of highly selective and active catalysts with low cost for electrolytic H_2_O_2_ production in acids is highly sought after.

In acids, the selective production of H_2_O_2_ via ORR requires the catalyst materials to adsorb the reaction intermediate, namely OOH*, with a suitable binding energy that could facilitate its single-electron reduction to H_2_O_2_, but suppress it from being further reduced or dissociated to O* and OH* that will eventually form H_2_O^7,22^. Thus far, porphyrins containing 3d transition metals (such as Fe, Ni, and Co) with an optimal binding energy toward OOH* are found to be a type of effective and selective homogeneous catalysts for the electrolytic production of H_2_O_2_ in acids^28^, while the unstable N ligands under the harsh reaction conditions will inevitably result in rapid performance losses^29^. Therefore, it is of paramount importance to develop an effective method to stabilize these metal-N active species as well as maintain their high selectivity towards H_2_O_2_ production under the corrosive reaction environments, such as strong acids.

Forming single-atom catalysts (SACs) on a carbon substrate seems an appealing solution to stabilize the N-coordinated transition-metal centers (metal-N_*x*_). Apart from that, the conductive nature of a carbon substrate will also enable the maximal exposure of the active metal-N_*x*_ sites. Compared with other transition metals (like Fe), Co-based composites exhibit less deleterious effects (like the active sites demetalation and Fenton reaction issue) that would eventually lead to the deactivation of the catalysts in acid^30^. Thus, in this work, a Co and N co-doped carbon nanotubes (CoN@CNTs) composite is prepared and employed as an electrocatalyst for ORR to produce H_2_O_2_ in acids. Moreover, by modifying the oxygen functionalities around the Co–N_*x*_ sites, significantly enhanced H_2_O_2_ catalytic performances can be obtained, showing both high activity (nearly zero overpotential) and selectivity (~100%), and exceeding nearly all the state-of-the-art catalysts and benchmarks reported in literature. Identification of specific oxygen functional groups in promoting the H_2_O_2_ production is carried out through a suite of spectroscopic measurements, revealing a synergy between epoxy O and Co–N_4_ centers, as further confirmed by computational modeling. Finally, by employing the epoxy functionalized CoN@CNTs as the cathode catalyst, a customized H_2_O_2_ electrolyzer is fabricated that can easily produce over 0.1 wt% (1199 ppm) H_2_O_2_ within 30 min, satisfying most of applications with an electro-Fenton process in water treatment (>10 ppm)^1^.

## Results

### Synthesis and characterizations of CoN@CNTs catalysts

**Fig. 1:**
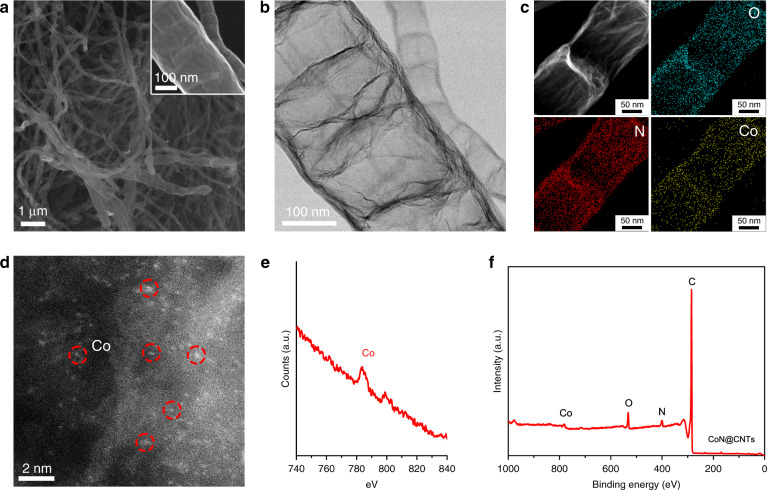
Microstructural analysis of the CoN@CNTs catalyst. **a** SEM images of CoN@CNTs. Inset is the high-resolution SEM image of a selected area of CoN@CNTs. **b** TEM image of CoN@CNTs showing a bamboo-like structure of the carbon nanotubes. **c** HAADF–STEM image and corresponding EDS maps of CoN@CNTs for O, N, and Co. **d** HAADF-STEM image of the CoN@CNTs showing many Co atoms (circled in red) well-dispersed in the carbon layers. **e** EELS analysis of selected area in **d** showing the signals of Co. **f** XPS elemental survey of CoN@CNTs.

The CoN@CNTs composite was prepared by pyrolyzing the carbon and metal precursors in Ar, followed by an acid leaching to remove the accessible metallic debris (see details in the “Methods” section). To characterize the typical structural morphologies of CoN@CNTs, the scanning electron microscopy (SEM) and transition electron microscopy (TEM) were firstly employed. The SEM images (Fig. 1a) show that numerous carbon nanotubes were formed after pyrolysis. Besides that, a bamboo-like feature of the nanotube structure is revealed by the TEM images (Fig. 1b and Supplementary Fig. 1), showing diameters ranging from 50 to 200 nm. Further conducting the high-resolution TEM (HR-TEM) (Supplementary Fig. 1e–g) confirms the graphitic characteristic of the nanotube composite, showing an interlayer spacing of ~0.35–0.36 nm on the CoN@CNTs, which could be ascribed to the (002) plane of graphitic carbon. Notably, the slightly larger d-spacing of the (002) plane than that in a well-ordered structure of graphite (0.33 nm) indicates a somewhat disordered structure of the carbon basal plane in CoN@CNTs, which might be caused by the incorporation of N and Co^31^. The energy dispersive X-ray spectroscopy (EDS) elemental mapping of a selected area on the CoN@CNTs composite (Fig. 1c and Supplementary Fig. 2) exhibits uniform distributions of the Co, N, O, and C elements across the tubular structure, demonstrating the successful incorporation of Co, N, and O into the carbon substrate. To identify the status of the Co species in CoN@CNTs, the high-angle annular dark field-scanning transmission electron microscopy (HAADF-STEM) measurement was conducted. As shown in Fig. 1d, the bright dots corresponding to Co atoms are homogenously dispersed throughout the CNTs. The statistical distribution of 50 pairs of two adjacent cobalt atoms shows that the Co–Co distances are in the range from 0.2 to 0.8 nm and the majority (~80%) exhibits a distance >0.4 nm (Supplementary Fig. 3), which suggests the dissociation of OOH* intermediate or re-adsorption of hydrogen peroxide by the adjacent Co atoms (less than 4 Å) for the further reduction to water is less likely^7^. Electron energy loss spectroscopy (EELS) analysis (Fig. 1e) of an area within Fig. 1d further reveals the existence of isolated Co atoms in the carbon matrix. In addition, a very limited amount of Co nanoparticles encapsulated inside 5 to 7 layers of graphitic carbon shells are also observed at the closed end of several carbon nanotubes (Supplementary Fig. 4), while this Co/C core-shell structure imposes negligible impacts on the electrolytic H_2_O_2_ production (see details in Supplementary Materials). The X-ray photon spectroscopy (XPS) elemental survey **(**Fig. 1f) also confirms the existence of Co, N, O, and C on the CoN@CNTs. To further reveal the nature of the isolated Co atoms in CoN@CNTs, X-ray absorption near-edge structure (XANES) and extended X-ray absorption fine structure (EXAFS) measurements (Supplementary Fig. 5) were performed. The higher white line intensity and position compared with Co^0^ indicates the existence of positively charged Co with an oxidation state in CoN@CNTs. Furthermore, the slightly lower absorption edge position than that of cobalt(II) phthalocyanine (CoPc) suggests the valence state of atomic Co is situated between 0 and 2+. The nearly absent appearance of a pre-edge feature (arisen from the forbidden 1s-to-3d transition) at ~7709 eV in the CoN@CNTs indicates a symmetric coordination environment of Co, e.g., Co–N_4_^32^. Notably, the Fourier transformed (FT) Co K-edge EXAFS spectra exhibit the signal of light scattering nearest neighbors at ~1.5 Å, corresponding to the Co–N/C scattering pair^33,34^ and further corroborating the existence of tetrahedrally coordinated Co (e.g., Co–N_4_)^35,36^ as revealed by fitting analysis (Supplementary Table 1). The weak signals originated from the Co–Co scattering path in a metallic feature might be ascribed to the rare appearance of Co nanoparticles.

### Electrochemical measurements and study of active sites

**Fig. 2:**
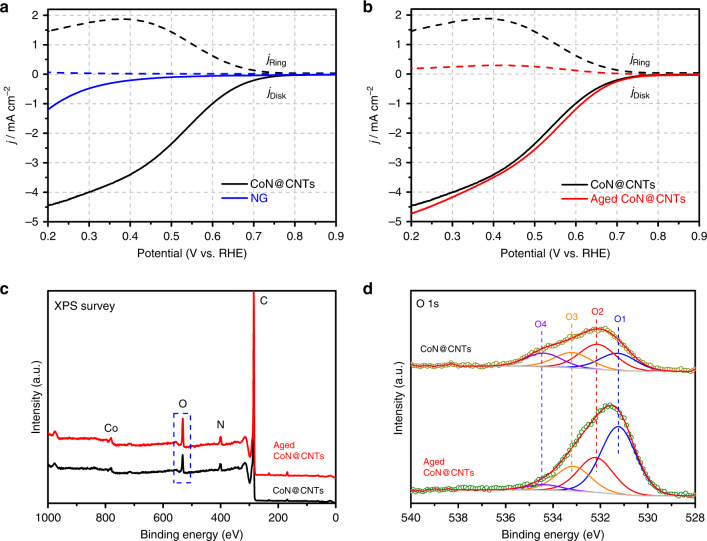
Oxygen reduction performance of CoN@CNTs. **a** RRDE voltammograms of CoN@CNTs and NG at 1600 rpm in an O_2_-saturated 0.1 M HClO_4_ electrolyte with disc current and ring current. **b** RRDE voltammograms of fresh and aged CoN@CNTs at 1600 rpm in an O_2_-saturated 0.1 M HClO_4_ electrolyte with disc current and ring current. All potentials are recorded without iR correction. **c** XPS elemental survey of CoN@CNTs and aged CoN@CNTs. **d** XPS O 1s spectra of CoN@CNTs and aged CoN@CNTs.

The electrocatalytic performances and efficiency of CoN@CNTs for H_2_O_2_ production via ORR were examined in O_2_-saturated 0.1 M HClO_4_ using a rotating ring-disk electrode (RRDE) setup. The Pt ring electrode was held at 1.2 V to quantify the amount of H_2_O_2_ produced on the disk electrode (see calculation details in the “Methods” section). Figure 2a shows the polarization curve obtained on CoN@CNTs, with the oxygen reduction current measured on the disk electrode (solid lines) and the H_2_O_2_ oxidation current measured on the Pt ring electrode (dashed lines). It is apparent that the CoN@CNTs composite exhibits a decent catalytic activity towards the electrolytic production of H_2_O_2_ in acid, showing obvious H_2_O_2_ oxidation currents at a potential range below 0.7 V. Notably, the majority of electrons is consumed by a 2-electron pathway on the CoN@CNTs, showing a high H_2_O_2_ selectivity (~80% from 0.5 to 0.7 V, Supplementary Fig. 6), as determined by RRDE measurements. In contrast, the FeN@CNTs and NiN@CNTs composites prepared via a similar method with an analogous nanotube structure (Supplementary Fig. 7) both exhibit a low H_2_O_2_ productivity and selectivity (<40%) within the same potential range, demonstrating the intrinsic active nature of Co–N_4_ species in selectively reducing O_2_ to H_2_O_2_^37^. Moreover, the Tafel slope of CoN@CNTs is much lower than that of FeN@CNTs and NiN@CNTs (Supplementary Fig. 8), indicating faster ORR kinetics on CoN@CNTs and further suggesting the superior role of Co–N_4_ species in reducing oxygen.

Furthermore, in the absence of Co species, nitrogen-doped graphene (NG) with a similar amount of N dopants (Supplementary Table 2) to the CoN@CNTs composite exhibits negligible H_2_O_2_ production within the whole potential range tested herein (Fig. 2a, blue lines), confirming the prerequisite role of Co–N_4_ in enabling the efficient H_2_O_2_ generation via ORR. The same conclusion can also be obtained with the poisoning experiment, where a significantly receded *j*_disk_ was detected after the Co–N_4_ centers were poisoned by thiocyanate ions (SCN^−^) (details can be found in Supplementary Fig. 9). Similarly, single Co atom embedded mesoporous carbon (M–CoNC, Supplementary Fig. 10) prepared via a different method also exhibits a H_2_O_2_-favored ORR process within the same range of potentials, demonstrating the 2-electron-transfer pathway is dominating the O_2_ reduction process on the Co–N_4_ sites in acid and precluding the possible effect from the Co/C core-shell structure. Notably, during a three-hour H_2_O_2_ production session (Supplementary Fig. 11), the selectivity is maintained at ~78% with negligible drop, showing a decent stability on the CoN@CNTs under the static operation conditions.

However, despite its high catalytic performance, after a long-term (~30 days) exposure to air, the selectivity of the CoN@CNTs composite towards H_2_O_2_ production falls dramatically. Figure 2b compares the RRDE curves obtained with freshly prepared CoN@CNTs and CoN@CNTs that has been exposed to air for one month (named as aged CoN@CNTs). It can be seen from Fig. 2b that *j*_ring_ on the aged CoN@CNTs drops significantly meanwhile the *j*_disk_ remains almost unchanged (compared with CoN@CNTs), leading to the H_2_O_2_ selectivity <30% over the whole potential range. This phenomenon indicates the occurrence of some unwanted oxidation process on the CoN@CNTs composite during the air exposure that has jeopardized its H_2_O_2_ productivity. This assumption is further confirmed by the electrochemical measurements of the CoN@CNTs composite that has been stored in vacuum for the same duration, exhibiting almost identical ORR activity and H_2_O_2_ selectivity (Supplementary Fig. 12). XPS was utilized to reveal the chemical changes between the CoN@CNTs and aged CoN@CNTs composites. As displayed in Fig. 2c, Supplementary Fig. 13 and Supplementary Table 2, the only noticeable change between these two samples is the increment of O concentration, which rises from 3.50 at% in CoN@CNTs to 5.17 at% in aged CoN@CNTs. More details are provided by the high-resolution XPS O 1s spectra (Fig. 2d) of the two composites, which involve four oxygen components^38,39^: (i) ketonic oxygen (C=O, O1, 531.2 ± 0.2 eV), (ii) oxygen atoms in epoxy (C–O–C) or hydroxyl groups and carbonyl oxygen in ester groups (O2, 532.3 ± 0.2 eV), (iii) the epoxy oxygen in ester groups (O3, 533.3 ± 0.2 eV), and (iv) oxygen atoms in carboxyl groups (O4, 534.2 ± 0.2 eV). Among all the four O functionalities detected within the composite, only the ketonic groups (O1) have gone through a noticeable increment, of which the concentration has increased from merely 0.84 at% in the fresh sample to 2.64 at% in the aged sample (Supplementary Table 3). This observation suggests that the ketonic groups might be responsible for the receded H_2_O_2_ productivity on the aged CoN@CNTs. Therefore, further study is warranted to find an effective method to mitigate the influence of this negative effect.

**Fig. 3:**
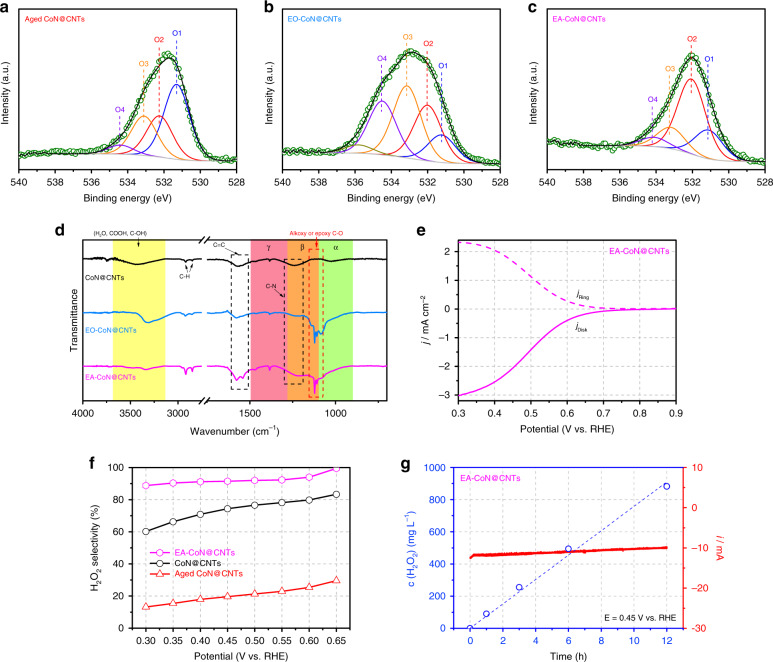
Electrochemical activation (EA) of CoN@CNTs. XPS O 1s spectra of **a** aged CoN@CNTs, **b** EO-CoN@CNTs and **c** EA-CoN@CNTs. **d** Background-corrected FTIR spectra of CoN@CNTs before and after ETs. **e** RRDE voltammograms of CoN@CNTs after EA (including EO and then ER) at 1600 rpm in an O_2_-saturated 0.1 M HClO_4_ electrolyte with disc current and ring current. **f** Calculated H_2_O_2_ selectivity on CoN@CNTs, aged CoN@CNTs and EA-CoN@CNTs based on the RRDE measurements. **g** H_2_O_2_ production amount (determined via the potassium permanganate titration) as a function of time on the EA-CoN@CNTs. Current (red curve) and concentration (blue ○) behavior with time for the electrochemical H_2_O_2_ production is shown.

Electrochemical treatment (ET) is selected here as an approach to in situ re-construct the surface oxygen functionalities on the carbon-based materials. Nevertheless, simply doing ER is hard to remove those oxygen functional groups (like ketonic O) that are thermodynamically more stable than the carboxyl groups^40^. As expected, the aged CoN@CNTs before and after ER treatment exhibits almost identical chemical properties (Supplementary Fig. 14) as well as H_2_O_2_ productivity (Supplementary Fig. 15), suggesting the ineffectiveness of ER in reconstructing the oxygen functionalities on CoN@CNTs. Compared with the ER process, the electrochemical oxidation (EO) treatment is more effective in rebuilding the surface oxygen functional groups on carbon-based materials^41^. Thus, herein, a two-step electrochemical process combing both EO and ER is adopted to treat the aged CoN@CNTs to modify the O functionalities. Firstly, the aged CoN@CNTs was electrochemically oxidized in the 0.1 M HClO_4_ solution (EO-CoN@CNTs) by an anodic linear sweep voltammetric scan (details can be referred to Supplementary Fig. 16). Based on the post-reaction XPS measurements (Fig. 3b), the EO treatment endows the aged CoN@CNTs composite a higher O content (12.66 at%, Supplementary Table 2) owing to the highly positive potential applied that caused carbon oxidation. Specifically, the EO-CoN@CNTs exhibits a higher ratio of O2 and O4, while that of ketonic O (O1) decreases, indicating the electrochemical generation of epoxy and carboxyl groups on the CoN@CNTs and a possible conversion of C=O into other O species during this treatment. Then, the EO-CoN@CNTs was subjected to an ER treatment to afford the electrochemically activated CoN@CNTs (EA-CoN@CNTs, Supplementary Fig. 17). From the XPS O 1s spectrum (Fig. 3c) of the EA-CoN@CNTs, it is interesting to note that, after ER process, the ratios of O3 and O4 both drop dramatically (from 33.2 and 24.7% to 15.9 and 6.9% respectively) while that of the O2 becomes dominant (55.8%) compared with those of EO-CoN@CNTs (Supplementary Fig. 18). The significantly reduced amount of O3 and O4 can be correlated to the reduction of some O functional groups (like ester and carboxyl) under a cathodic potential, coinciding with the observations in the previous work^40^. Epoxy groups are thermodynamically more stable than ester/carboxyl groups^38,40^, and they are hard to be removed by ER. Therefore, compared with the aged CoN@CNTs, the exclusively increased O2 species in the EA-CoN@CNTs can be attributed to the apparent emergence of epoxy groups on the catalysts. Fourier-transform infrared spectroscopy (FTIR) measurements (Fig. 3d) further confirm the appearance of these epoxy functionalities on the EA-CoN@CNTs, showing a strong and broad absorption band (alkoxy or epoxy C–O) at a region from 1000 to 1200 cm^−1^ as reported in recent studies^42,43^.

To understand the impact of O functionalities on the Co–N_4_ centers, N 1s and Co 2p XPS spectra were analyzed on the electrochemically treated samples. The N 1s spectrum shows that pyridonic N has increased substantially after ETs (details can be referred to Supplementary Fig. 19) while the concentration of pyridinic N has decreased, suggesting a possible conversion between these two N species. Thus, it is plausible to postulate that during the ETs, the epoxy groups produced will preferably bond to C atoms that are adjacent to the pyridinic N (coordinated with Co atoms), and consequently transform the pyridinic N to pyridonic N^44^. This postulation is further supported by the Co 2p_3/2_ XPS spectrum presented in Supplementary Fig. 20, where the Co–N peak in EA-CoN@CNTs shifts positively to a higher binding energy side (~780.7 eV) than that in the pristine CoN@CNTs (~780.4 eV)^45^, demonstrating the electro-generated O groups (e.g. epoxy) could be in the vicinity of Co–N_x_ centers and pose an electron-withdrawing effect that has affected the electronic structure of the Co atoms. In contrast, the ETs show no obvious influence on the valence state of Co species, reflected by the similar spin-energy separation values of samples before and after ETs (Supplementary Fig. 20). Moreover, no leaching of Co in the electrolyte was detected by the inductively coupled plasma mass spectroscopy (ICP-MS) measurement during the ETs, indicating the chemically stable property of the Co sites.

To testify the effectiveness of the ETs, electrolytic H_2_O_2_ production over the EA-CoN@CNTs was also evaluated in O_2_-sat. 0.1 M HClO_4_ solution. Surprisingly, EA-CoN@CNTs exhibits a superior H_2_O_2_ productivity that is even higher than the freshly prepared CoN@CNTs. Specifically, the onset potentials of EA-CoN@CNTs at the ring and disk coincide at ~0.7 V (Fig. 3e), which is the thermodynamic onset potential of the genuine 2-electron pathway of ORR in acid for H_2_O_2_ production^22^. As the overpotential increases, most of the *j*_disk_ recorded on EA-CoN@CNTs can be accounted for the production of H_2_O_2_. The selectivity of H_2_O_2_ production is well above 90% within a region between 0.3 and 0.6 V (Fig. 3f) based on the RRDE measurement, indicating a nearly complete 2-electron ORR process on the EA-CoN@CNTs. Also, at a potential of 0.3 V, the *j* on the disk (0.125 cm^2^) achieves 3 mA cm^−2^, which is close to the theoretical mass transport limit for the 2-electron reduction of oxygen^22^. To investigate the Faradaic efficiency (FE) and stability of EA-CoN@CNTs during bulk electrolysis, the electrocatalytic H_2_O_2_ production and accumulation through ORR was carried out in a H-cell setup separated by a Nafion membrane with continuous O_2_ bubbling. The FE was verified for the purpose of a more intuitive demonstration employing permanganate titration (average of three repetitions)^22^ within a range of potentials from 0.45 to 0.65 V. Of note, the titration results (Supplementary Fig. 21) exhibit a FE as high as 95%, which coincides with the RRDE results. The long-term stability performed via a chronoamperometric measurement at 0.45 V for 12 h (Fig. 3g) yields a current of ~10 mA, during which the concentration of accumulated H_2_O_2_ also increased continuously. The slight drop in current was attributed to the possible attachment of some species (like cations or H_2_O_2_) on the surface of electrode, and this can be recovered after changing the electrolyte and rinsing the electrode (Supplementary Fig. 22). From the electrochemical results shown above, it is evident that the synergistic effect between Co–N_4_ centers and their adjacent C–O–C groups plays an advocating role in reducing oxygen exclusively to H_2_O_2_, which makes the EA-CoN@CNTs among the most effective catalyst for electrolytic H_2_O_2_ production in acids (Supplementary Table 4).

**Fig. 4:**
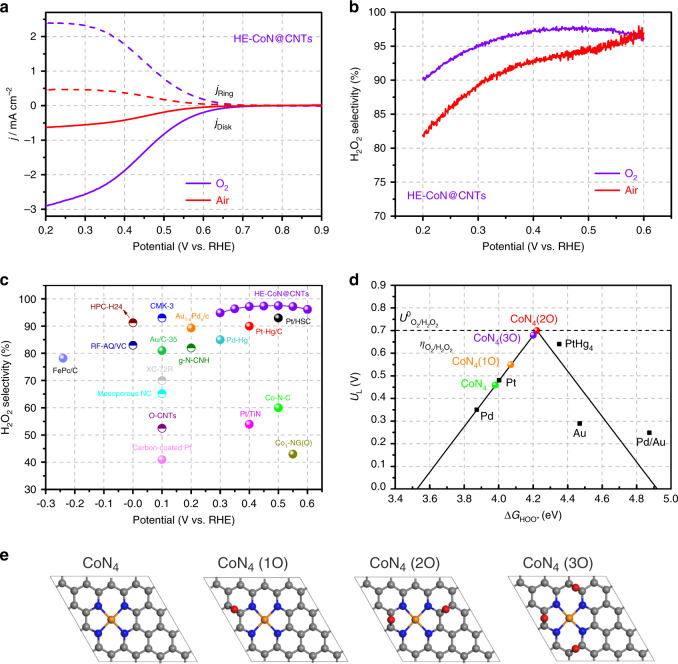
ORR activities of HE-CoN@CNTs and DFT results of different types of epoxy-modified Co–N_4_ sites. **a** RRDE measurements of HE-CoN@CNTs for ORR in 0.1 M HClO_4_ solution purged with O_2_ and air. **b** Calculated H_2_O_2_ selectivity on HE-CoN@CNTs in O_2_- and air-saturated 0.1 M HClO_4_ based on the RRDE measurements. **c** H_2_O_2_ selectivity on HE-CoN@CNTs and other recently reported catalysts (Supplementary Table 4) in acidic media. **d** Calculated Sabatier volcano plot for the 2-electron ORR to H_2_O_2_ for bare CoN_4_ moiety and CoN_4_ moieties with different epoxy oxygen coverages obtained from DFT simulations. The U_L_ is plotted as a function of $\Delta {\mathrm{G}}_{{\mathrm{HOO}}^ \ast }$. The black solid lines represent the theoretical Sabatier volcano^22^. The black squares are the data adapted from ref. ^22^. **e** The optimized geometry structures of bare CoN_4_ moiety and CoN_4_ moieties with different epoxy oxygen coverages, which were chosen to calculate the ORR activities in this work. Here, CoN_4_ indicates bare CoN_4_ moiety. CoN_4_ (1O), CoN_4_ (2O), and CoN_4_ (3O) indicate CoN_4_ moieties with 1, 2 or 3 epoxy oxygen, respectively. The gray, blue, orange and red balls represent C, N, Co and O atoms, respectively.

C–O–C groups on carbon can also be introduced through chemical approaches and H_2_O_2_ treatment (HT) was previously found to be an effective method^46^. Hence, to further understand the critical role of epoxy functionalized Co–N_4_ centers in promoting the 2-electron ORR for H_2_O_2_ production, aged CoN@CNTs sample was chemically treated in 0.1 M HClO_4_ solution containing 5 wt% H_2_O_2_ at 70 °C for 2 h. A dominant presence of epoxy groups is detected by both XPS (Supplementary Fig. 23a) and FTIR (Supplementary Fig. 23b) on the H_2_O_2_-treated CoN@CNTs (HT-CoN@CNTs), accompanied by a significantly reduced intensity of the ketonic group, suggesting the effectiveness of this treatment. As expected, HT-CoN@CNTs exhibits a much higher H_2_O_2_ selectivity (Supplementary Fig. 24a) as well as productivity (Supplementary Fig. 24b) than the aged CoN@CNTs, echoing the findings from the ETs. Thus, to further enhance the H_2_O_2_ productivity of the CoN@CNTs composite, both HT and electrochemical activation (EA) methods were applied together (see details in the “Methods” section) on the aged CoN@CNTs sample to obtain the HE-CoN@CNTs. As expected, a high content of O (12.3 at%) and the highest ratio of epoxy groups (71.3%) among all samples prepared were obtained (Supplementary Fig. 25a and Table 3), accompanied by an obviously increased ratio of pyridonic N (Supplementary Fig. 25b). The HE-CoN@CNTs exhibits, to the best of our knowledge, a record-breaking catalytic performance towards H_2_O_2_ production in acid. Shown in Fig. 4a, b, with the onset potential at 0.7 V, the HE-CoN@CNTs composite is capable of maintaining a nearly 100% selectivity of H_2_O_2_ production within a wide potential range from 0.3 to 0.6 V (>95%), exceeding the catalytic performances of those benchmarks and state-of-the-art catalysts, including precious metals, their alloys and recently reported carbon-based materials (Fig. 4c, Supplementary Table 4), in terms of both overpotential and selectivity. In contrast, the NG exhibits a much lower H_2_O_2_ current within the same potential range even after the H_2_O_2_ treatment and ETs (Supplementary Fig. 26), further advocating the importance of synergies between CoN_4_ active sites and nearby C–O–C groups. Post-reaction characterizations (XPS and FTIR, Supplementary Figs. 27 and 28) show negligible changes on the HE-CoN@CNTs after a 12-h reaction session, evidencing the fact that not only the Co species, but also the epoxy groups are stable during ORR. In addition, the catalytic activity and H_2_O_2_ selectivity is kept almost unchanged on the HE-CoN@CNTs sample even after long-term aging period in air. This is due to the chemically stable property of these epoxy functionalities on catalysts that eventually benefit a well retained H_2_O_2_ selectivity after aging, as revealed by FTIR and XPS O 1s spectra (Supplementary Fig. 29). Moreover, within a gas diffusion electrode (GDE, Supplementary Fig. 30) setup, the H_2_O_2_ concentration could be easily accumulated to 1199 ppm within 30 min at 0 V, which is sufficient for direct usage in industries like water disinfection and bleaching^1^. At a more moderate potential (0.3 V, Supplementary Fig. 31), 5525 ppm of H_2_O_2_ is produced within 12 h, giving the energy-efficient feature of this process as less overpotentials are required. Notably, even when the reaction is conducted using air bubbling (Fig. 4b), the H_2_O_2_ selectivity is still maintained at >80% within the whole voltage range, demonstrating its potential to be applied in practical applications.

To further corroborate the correlation between the emerged epoxy oxygen and the enhanced H_2_O_2_ selectivity, near-edge X-ray absorption fine structure (NEXAFS) measurements were performed on the post-treated CoN@CNTs samples. As shown in Supplementary Fig. 32a, high-resolution oxygen K-edge NEXAFS results of EO-CoN@CNTs, EA-CoN@CNTs, HT-CoN@CNTs and HE-CoN@CNTs all exhibit obvious features at ~533.4 and 534.8 eV, corresponding to transitions of O 1s core level electrons to final states of π* symmetry localized at C–O bond of epoxy groups^14,47–49^. The intense peak arisen at around 539 eV in the spectra curves of those samples after chemical and/or ETs can be ascribed to the transitions to σ* states of C–O bonding from ring C–O–C functionalities^48^, further confirming the emergence of epoxy O. Notably, the bisphenol A diglycidyl ether (BADGE) composite gives rise to obvious π* and σ* resonances at around 534.2 and 539.0 eV (Supplementary Fig. 32a), respectively, signifying the emergence of C–O–C groups in our samples. The split of antibonding π* symmetry of C–O bond in the post-treatment CoN@CNTs is probably ascribed to the high coverages of epoxy functionalities on the surface of carbon basal plane^47^. In addition, Co L-edge spectra (Supplementary Fig. 32b) show significant fine-structure at L_3_ peak due to the multiplet structure from electron-electron interaction, which depends greatly on the local coordination symmetry of Co sites. As expected, the CoN@CNTs sample exhibits the signatures of CoN_*x*_ moieties very close to Co–N–C catalysts in previous reports^30^, suggesting a tetrahedrally coordinated (T_d_) symmetry of Co atoms in our catalysts, which is consistent with the XANES Co K-edge results. The spectra of EA-CoN@CNTs, HE-CoN@CNTs and EO-CoN@CNTs show promiment fine-structure features at 778.5 and 779.0 eV, which are similar to the oxygen-induced distored CoN_4_ functionalities as reported previously^50^, causing a “octahedral-like” symmetry on the Co sites by nearby O atoms. Notably, the increases in overall intensity for those epoxy-modified samples (EA-CoN@CNTs, HE-CoN@CNTs and EO-CoN@CNTs) are acribed to electrons transferring away from the Co atoms, strongly implicating that nearby O atoms from epoxy groups are modifying the electronic structure of the Co sites (e.g., electron-withdrawing effect) and thus their ability to selectively produce hydrogen peroxide. NEXAFS Co L-edge spectrum from bulk Co_3_O_4_ reference is shifted to higher energy as expected for increase in oxidation state, suggesting that bulk oxidiation is not found in our materials during the electrochemical and chemical treatments.

### Theoretical simulations

The above experimental results demonstrated that the electrochemical/chemical treatments of CoN@CNT generate abundant amounts of epoxy groups, which may tailor the electronic structure of the CoN_4_ moieties and significantly improve their 2-electron-pathway ORR activity for the H_2_O_2_ production. To elucidate the mechanism within this enhancement effect, density functional theory (DFT) calculations were performed. Extensive details regarding the calculations are available in the Supplementary Materials. Here we considered epoxy oxygen on CoN_4_ catalysts with different coverage and locations (Supplementary Figs. 33 and 34), and chose the possible structures (Fig. 4e) with low epoxy oxygen formation energy ($E_{{\mathrm{ep}} - {\mathrm{O}}}^F$) to calculate the ORR activity. Supplementary Fig. 35 shows the free-energy diagrams for the 2-electron ORR to H_2_O_2_ at the equilibrium potential ($U_{{\mathrm{O}}_2/{\mathrm{H}}_2{\mathrm{O}}_2}^0 = 0.70\,{\mathrm{V}}$), and the theoretically calculated ORR volcano plot for the 2-electron pathways is given in Fig. 4d. It can be seen from Fig. 4d and Supplementary Fig. 35 that the introduction of epoxy groups close to CoN_4_ moiety dramatically weaken the binding strength of HOO* on the Co atoms. Especially for the structures of CoN_4_ moiety with 2 or 3 epoxy oxygens, the computed values of $\Delta G_{{\mathrm{HOO}}^ \ast }$ for CoN_4_ (2O) and CoN_4_ (3O) are 4.22 and 4.20 eV, respectively, which are located at the peak of the 2-electron volcano with *U*_L_ = 0.70 and 0.68 V, respectively. These results suggest that the CoN_4_ moiety with nearby epoxy groups exhibits their superior activity and selectivity of the 2-electron ORR to H_2_O_2_ with the overpotentials less than 0.02 V, which are comparable or even lower than that of the state-of-the-art PtHg_4_ for the 2-electron ORR to H_2_O_2_ in acidic conditions (*η* < 0.10 V)^22^. In contrast, the epoxy oxygen formed at the carbon atoms far from the Co–N_4_ centers (Supplementary Fig. 33) exhibits negligible effect in optimizing the binding energy of HOO* intermediate at Co sites, indicating the importance of synergies between CoN_4_ moieties and those nearby epoxy groups. The Bader analysis reveals that the decrease of ${\mathrm{\Delta }}G_{{\mathrm{HOO}}^ \ast }$ for CoN_4_ moiety with epoxy groups is due to the electro-withdrawing groups^51^, i.e., epoxy oxygen, deplete the electrons on Co atom of CoN_4_ moiety. For example, the electrons on Co are +0.92*e* and +0.97*e* for CoN_4_ and CoN_4_ (3O), respectively, based on the Bader analysis, echoing with the electron-transferring effect between Co and nearby O atoms observed in NEXAFS measurements. Furthermore, the climbing image nudged elastic band (CI-NEB) method is also performed to calculate the chemical dissociation barrier of HOO* on the active site, elucidating the origin of the H_2_O_2_ selectivity on the epoxy-modified CoN_4_ sites. As expected, the dissociation energy of HOO* on the CoN_4_ sites is high (1.4 eV), and that can be further increased after epoxy-modification (Supplementary Fig. 36), suggesting the possible O–O bond session could be effectively suppressed on the epoxy-modified CoN_4_ moieties that in turn benefit an improved H_2_O_2_ selectivity. This is further supported by the previous hypothesis that the chemical cleavage of O–O bond by the adjacent carbon atoms near the M–N sites could be sterically blocked by their surrounding oxygen groups^46^.

## Discussion

While the above results unequivocally confirm the synergies between epoxy oxygen and CoN_4_ species in promoting the H_2_O_2_ productivity, the possible effect of limited amount of hydroxyl groups in influencing the ORR selectivity cannot be fully excluded. Thus, the role of hydroxyl oxygen on the H_2_O_2_ productivity is also evaluated as it may cast a similar influence on the CoN_4_ centers. As expected, simulation results reveal a similar electron-withdrawing effect of –OH functionalities on the CoN_4_ species (Supplementary Fig. 37), which might facilitate the H_2_O_2_ formation. Nevertheless, the binding energy of OOH* on the hydroxyl-modified CoN_4_ centers is still not as optimal as that on the epoxy-modified active sites (Supplementary Fig. 35). Moreover, in this study, the possible origin of H_2_O_2_ selectivity from the synergies between –OH species and CoN_4_ sites are further precluded by a set of experiments. Hot-alkaline treatment is regarded as effective in introducing –OH groups as well as converting C–O–C functionalities into other species^52^. Thus, the aged CoN@CNTs sample was heated in concentrated KOH solution (see details in “Methods”), generating an ample amount of hydroxyl oxygen but no obvious C–O–C groups were observed (Supplementary Fig. 38). Notably, no enhancement of H_2_O_2_ selectivity was identified on the hot-alkaline-treated sample, suggesting a limited effect of –OH groups in affecting the H_2_O_2_ formation. Therefore, it can be concluded that the significantly enhanced H_2_O_2_ selectivity on the CoN_4_ active sites is mainly due to the emergence of epoxy oxygen rather than hydroxyl groups near the Co atoms. Previous reports suggest that oxidation of carbon can significantly influence the H_2_O_2_ performance on M–N–C (M = Co, Fe, Mn) catalysts^46,53^. However, no direct correlations between specific oxygen functionalities and H_2_O_2_ selectivity has been established, causing a debate on the real origin of H_2_O_2_ selectivity as the influence of diverse O groups cannot be universal. Herein, the enhancement effect of epoxy groups on H_2_O_2_ selectivity is answered systematically, providing guidelines in not only design of efficient catalysts for peroxide production but also stabilizing the performance of SACs in acidic fuel cells. Further, all these experiments and simulations strongly prove the effectiveness of our electrochemical/chemical approach in tuning the selectivity of O_2_ reduction through surface reconstruction on carbon-based materials, which could be readily adopted for other selectivity-critical electrochemical reactions, like N_2_ or CO_2_ reduction.

In summary, the DFT calculation provides an explanation for the synergistic effects observed experimentally over the EA-CoN@CNTs and HE-CoN@CNTs composites with significantly improved activity as well as selectivity towards H_2_O_2_ production in acidic electrolytes. The electronic structure of Co–N_4_ centers has been tuned by the presence of epoxy groups that are adjacent to them, resulting in the close-to-ideal binding energy that allows the ORR to proceed via a nearly complete two-electron transfer pathway to produce H_2_O_2_. Both the EA-CoN@CNTs and HE-CoN@CNTs composites exhibit record-breaking H_2_O_2_ productivity, outperforming nearly all the catalyst materials reported previously. Further optimizations of the catalyst materials as well as devices obtained in this study, including improved long-term stability (up to several thousand hours), faster mass transport in the electrolyzer and accelerated H_2_O_2_ production rate in air^54,55^, will be carried out to warrant their future practical applications.

## Methods

### Materials

All reaction reagents and chemicals were obtained and used in their as-received form without any further purification. Dicyandiamide, Co(NO_3_)_2_·6H_2_O, Ni(NO_3_)_2_·6H_2_O, Fe(NO_3_)_3_·9H_2_O, Nafion solution (5 wt%) and cobalt(II) phthalocyanine (CoPc) were obtained from Sigma-Aldrich. Deionized water was obtained through the water purification system (Milli-Q water) in the lab.

### Synthesis and surface treatments of CoN@CNTs

Typically, to prepare the CoN@CNTs catalyst, 3.5 mmol Co(NO_3_)_2_·6H_2_O, 35 mmol dicyandiamide and 2 mL ethanol were mixed in an agate mortar, followed by continuous grinding until the mixture forms a uniform pink paste. The as-obtained mixture was placed into a crucible boat and heated to 800 °C with a ramping rate of 3 °C min^−1^ for 3.5 h under Ar atmosphere. The impurities and undesirable nanoparticles outside the carbon nanotubes were removed through an acid leaching process in 0.5 M H_2_SO_4_ at 90 °C for 4 h, followed by repeatedly filtered and washed process in deionized water. The FeN@CNTs and NiN@CNTs were prepared using the similar pyrolysis process by changing the type of metal nitrate in the precursors. The effect of carbonization time on H_2_O_2_ productivity was investigated in detail, and we found that CoN@CNTs pyrolyzed at 800 °C for 3.5 h yields the highest ORR activity towards H_2_O_2_ production (Supplementary Fig. 39). The M–CoNC composite was synthesized via a modified method as reported previously^56^. The polymerized OPD (Phenylenediamine, Sigma Aldrich, 99.99%) in presence of hydrochloric acid and APS (Ammonium persulfate, Sigma Aldrich, 99.99%) was mixed with Co(NO_3_)_2_·6H_2_O and then pyrolyzed at 900 °C under Ar for 2 h to form M–CoNC. NG was directly purchased from Sigma Aldrich (99.99%). To obtain the HT-CoN@CNTs, the CoN@CNTs powder was dispersed and heated in 50 mL 0.1 M HClO_4_ solution containing 5 wt% H_2_O_2_ under 70 °C for 2 h. The EA-CoN@CNTs was obtained by oxidizing the CoN@CNTs electrochemically through conducting an anodic linear sweep voltammetric scan from 1.2 to 2.4 V vs. RHE, followed by an ER process via a cathodic linear sweep voltammetric scan from 0.6 to −0.6 V vs. RHE. To prepare the HE-CoN@CNTs, the CoN@CNTs sample was treated through a combined HT and EA process. Hot-alkaline treatment was conducted by heating the aged CoN@CNTs sample in 6 M KOH solution with an autoclave reactor under 180 °C for 12 h.

### Physicochemical characterizations

Transmission electron microscopy (TEM) was carried out on a Phillips/CM 200 microscope operated at an accelerating voltage of 200 kV. SEM was conducted on a JEOL 7001F operated at 5 kV. FTIR measurements were conducted on a PerkinElmer FTIR Spectrometer. X-ray photoelectron spectroscopy (XPS) measurements were carried out on a Thermo ESCALAB250Xi X-ray photoelectron spectrometer using Cu Kα X-rays as the excitation source with a voltage of 12.5 kV and power of 250 W. High-angle annular dark field-scanning transmission electron microscopy (HAADF-STEM) and energy dispersive X-ray (EDX) mapping were obtained on a spherical aberration corrected transmission electron microscope (FEI Titan G2 80–200) which was operated at 200 kV. ICP-MS was carried out using a PerkinElmer quadrapole Nexion instrument. Co K-edge X-ray absorption spectroscopy (XAS) measurements were performed at the 10-ID-B beamline of the Advanced Photon Source (APS), Argonne National Laboratory (ANL). Data were collected using a fluorescence geometry and scanned from 200 eV below the Co K-edge (7709 eV) to ~1000 eV past the edge. Data reduction and subsequent modeling efforts were performed using the Demeter software package^57^. Modeling results used a Co-(N_3_C_2_)_2_ cluster to modeling Co–N and Co–C backscattering paths^58^. An S_0_^2^ value of 0.776 was used for all models and obtained from modeling a reference Co foil. O K-edge and Co L-edge NEXAFS measurements were performed at the SXR beamline of the Australian Synchrotron. Powders were pressed onto Ir foil and mounted onto metallic sample holders for measurements under partial electron yield (PEY) mode. O K-edge measurements were performed from 520 eV to 580 eV while Co L-edge measurements were performed from 770 to 810 eV and internally calibrated using MnO and Co foil reference samples respectively. All data processing, energy calibrations, and normalization was performed using the program QANT.

### DFT calculations

All of the spin-polarized DFT calculations were performed using the VASP program^59–61^, which uses a plane-wave basis set and a projector augmented wave method (PAW) for the treatment of core electrons^59^. The Perdew, Burke, and Ernzerhof exchange-correlation functional within a generalized gradient approximation (GGA-PBE)^62^ was used in our calculations, and the van der Waals (vdW) correction proposed by Grimme (DFT-D3) was employed due to its good description of long-range vdW interactions^63^. For the expansion of wavefunctions over the plane-wave basis set, a converged cutoff was set to 400 eV. Dipole correction was considered in all cases. Extensive details were given in from Supplementary Figs. 33–37.

### Electrochemical measurements

The electrochemical tests were all performed in 0.1 M HClO_4_ aqueous solution within a three-electrode system at room temperature on a computer-controlled potentiostat (CH Instrument, CHI 760E). The oxygen reduction activity and selectivity were investigated by polarization curves and rotating ring-disk electrode (RRDE) measurements in oxygen-saturated electrolyte with a scan rate of 5 mV s^−1^. A glassy carbon electrode loaded with catalyst was used as the working electrode. A graphite rod and a saturated calomel electrode were used as counter and reference electrode respectively. To prepare the working electrode loaded with the catalyst, typically, 5 mg as-prepared catalyst and 25 μL Nafion solution (Sigma Aldrich, 5 wt %) were dispersed in 1 mL ethanol aqueous solution (50%) to form a homogeneous ink with the help of a sonication process for 30 min. Then, 6.5 μL of the ink was dropcasted onto the surface of a polished glassy carbon electrode and dried under room temperature in atmosphere. The final loading of the catalysts on working electrode was 0.25 mg cm^−2^. Commercially available Pt/C catalyst (20 wt% of Pt, Sigma-Aldrich) was employed as the benchmark for 4e^−^ ORR process to testify the electrochemical testing conditions in acid (Supplementary Fig. 40). All of the obtained potentials were calibrated to a reversible hydrogen electrode (RHE, *E*_RHE_ = *E*_SCE_ + 0.2415 + 0.059 × pH). No correction of the system resistance was employed. The Tafel slope was calculated using the Tafel equation: *η* = b log (*j*/*j*_o_) (η, b, *j*, and *j*_o_ represent the overpotential, Tafel slope, current density and exchange current density respectively).

H_2_O_2_ selectivity of the catalysts was calculated from the current of both disc and ring electrodes using the following equation:

1 $${\mathrm{H}}_{\mathrm{2}}{\mathrm{O}}_{\mathrm{2}}\left( {\mathrm{\% }} \right) = {\mathrm{200}} \times {\mathrm{I}}_{\mathrm{r}}{\mathrm{/N/}}\left( {{\mathrm{I}}_{\mathrm{d}}{\mathrm{ + I}}_{\mathrm{r}}{\mathrm{/N}}} \right)$$

where I_d_ is disk current, I_r_ is ring current, and N is current collection efficiency of the Pt ring. N was determined to be 0.32 in our system after calibration using the reversible [Fe(CN)_6_]^4−/3−^ redox couple (+0.36 vs. SHE). A potential of 1.2 V vs. RHE was applied on the Pt ring of the working electrode at a speed of 1600 r.p.m. during the entire testing process.

FE and electrocatalytic production of H_2_O_2_ were measured in a two-compartment cell with Nafion membrane as separator. Both the cathode compartment (75 mL) and anode compartment were filled with 0.1 M HClO_4_ aqueous solution. Oxygen was continuously purged into the cathode (working) compartment under vigorous stirring. A graphite rod and a saturated calomel electrode were used as counter and reference electrode respectively. A hydrophobic carbon fiber paper (CFP) loaded with as-prepared CoN@CNTs catalyst (0.5 mg cm^−2^) was used as the working electrode.

To quantify the amount of H_2_O_2_ produced, we carried out an independent test not based on electrochemical methods: permanganate titration. Typically, samples with a volume of 1 mL collected at certain time intervals were diluted into 10 mL and titrated with 0.005 M KMnO_4_ aqueous solution. The concentration of H_2_O_2_ produced was calculated according to the following equation:

2 $${\mathrm{C}}_{{\mathrm{H2O2}}} = {\mathrm{ 5C}}_{{\mathrm{KMnO4}}} \times {\mathrm{V}}_{{\mathrm{KMnO4}}} \div {\mathrm{2V}}_{{\mathrm{H2O2}}}$$

where C_H2O2_ is the H_2_O_2_ concentration (mol L^−1^), C_KMnO4_ is the precise concentration of KMnO_4_ solution (mol L^−1^), V_KMnO4_ is the volume of KMnO_4_ solution consumed during titration (mL), V_H2O2_ is the volume of H_2_O_2_ solution.

## Supplementary information

Supplementary Information

Peer Review File

## Data Availability

The data that support the plots within this paper and other findings of this study are available from the corresponding author upon reasonable request.
