# Supplementary material for: Direct insights into the role of epoxy groups on cobalt sites for acidic H2O2 production
Source: Nat Commun. 2020 Aug 21;11:4181. doi: 10.1038/s41467-020-17782-5 (PMC7442824; doi:10.1038/s41467-020-17782-5)
Supplement: Supplementary file 1 — Supplementary Information [file 41467_2020_17782_MOESM1_ESM.pdf]

<u>Supplementary Information</u>

Direct insights into the role of epoxy groups on cobalt sites for acidic H_2O_2 production

Zhang *et al*.

Direct insights into the role of epoxy groups on cobalt sites for acidic H_2O_2 production

Qingran Zhang[1], Xin Tan[2], Nicholas M. Bedford[1], Zhaojun Han[1,3,4], Lars Thomsen[5], Sean Smith[2], Rose Amal[1]*, Xunyu Lu[1]*

Affiliations:

[1]Particles and Catalysis Research Group, School of Chemical Engineering, The University of New South Wales, Sydney, NSW 2052, Australia

[2]Integrated Materials Design Laboratory, Department of Applied Mathematics, Research School of Physics and Engineering, The Australian National University, Canberra, ACT 2601, Australia

[3]School of Mechanical and Manufacturing Engineering, The University of New South Wales, Sydney, NSW 2052, Australia

[4]CSIRO Manufacturing, P.O. Box 218, 36 Bradfield Road, Lindfield, NSW 2070, Australia

[5]Australian Synchrotron, Australian Nuclear Science and Technology Organisation, 800 Blackburn Road, Clayton, Victoria, 3168, Australia

*Corresponding author: r.amal@unsw.edu.au, xunyu.lu@unsw.edu.au

This file includes:

I. Supplementary Notes

II. Supplementary Figures

III. Supplementary Tables

Supplementary Notes:

In this work, the electrochemical production of H_2O_2 proceeds via the cathodic reduction of O_2 (ORR), which can either produce the desired H_2O_2 via the 2-electron pathway or H_2O via the 4-electron pathway. Herein, we focus on the 2-electron pathway as the H_2O_2 is electrochemically produced on purpose.

2-electron-pathway ORR:

In acid (or pH < 11.6)

$O_2 + 2H^+ + 2e^- \rightarrow H_2O_2$ ($U_0^{O2/H2O2}$ = 0.7 V vs. RHE)

This reaction can be followed by either a further 2-electron reduction process

$H_2O_2 + 2H^+ + 2e^- \rightarrow 2H_2O$ ($U_0^{H2O2/H2O}$ = 1.76 V vs. RHE)

or a chemical disproportionation process

$2H_2O_2 \rightarrow 2H_2O + O_2$

In alkaline (or pH > 11.6, where products become HO_2^-)

$O_2 + H_2O + 2e^- \rightarrow HO_2^- + OH^-$ ($U_0^{O2/HO2-}$ = 0.74 V vs. RHE)

The reaction could also be followed by either a further reduction to OH^-

$HO_2^- + H_2O + 2e^- \rightarrow 3OH^-$ ($U_0^{HO2-/OH-}$ = 0.86 V vs. RHE)

or a chemical decomposition process

$2HO_2^- \rightarrow 2OH^- + O_2$

Supplementary Figures:

Supplementary Figures on the catalyst morphology and microstructure:

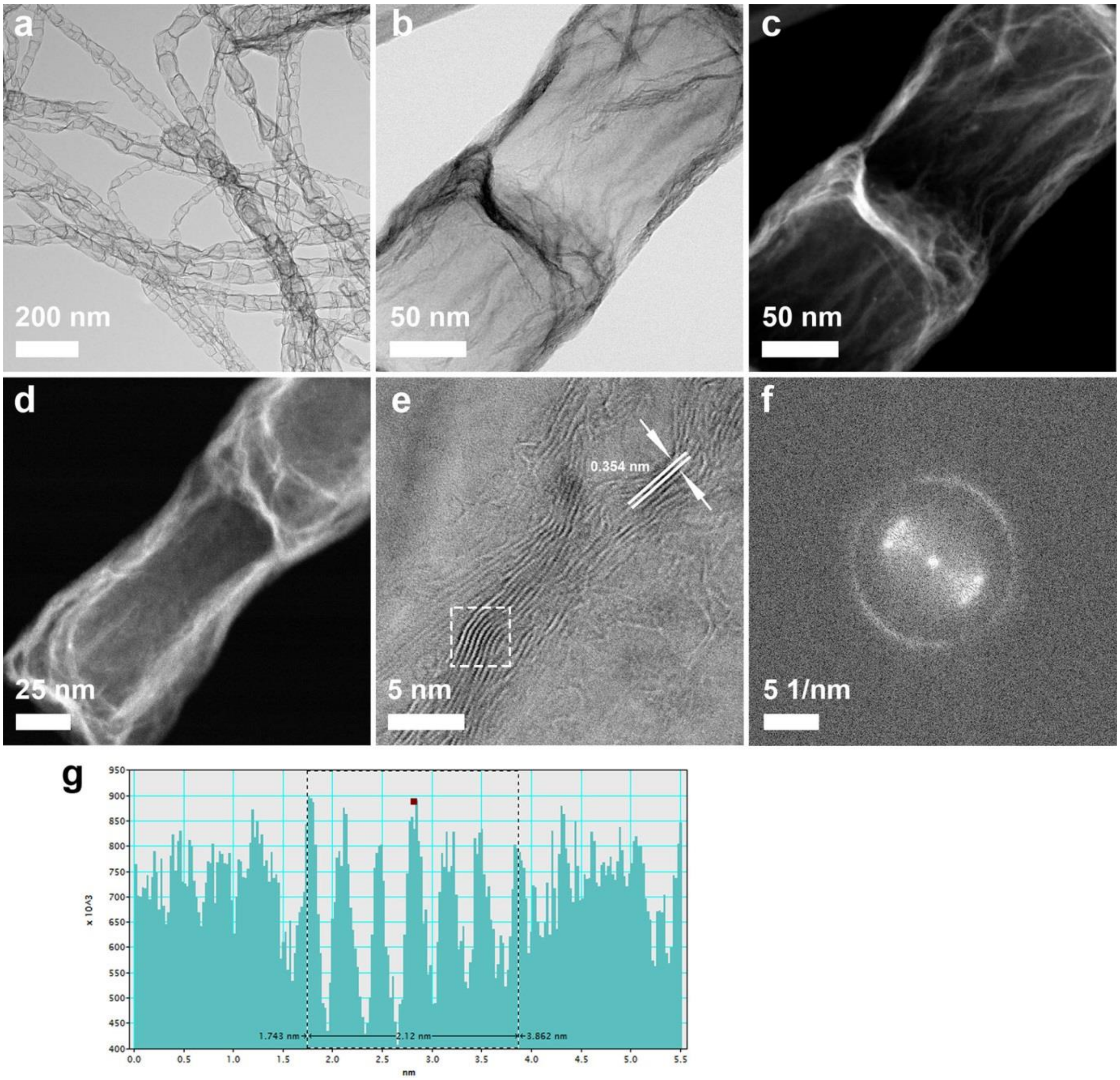

Supplementary Figure 1. (**a**, **b**) The TEM, (**c**, **d**) STEM and (**e**) HR-TEM images of CoN@CNTs, showing a bamboo-like structure and graphitic feature of CNTs. (**f**) Reduced fast Fourier transform (FFT) pattern of a selected area (dash line) given in Supplementary Figure 1e, showing an interlayer spacing of ~0.355 nm. (**g**) The distance profile of lattices shown in Supplementary Figure 1e (white dash line area).

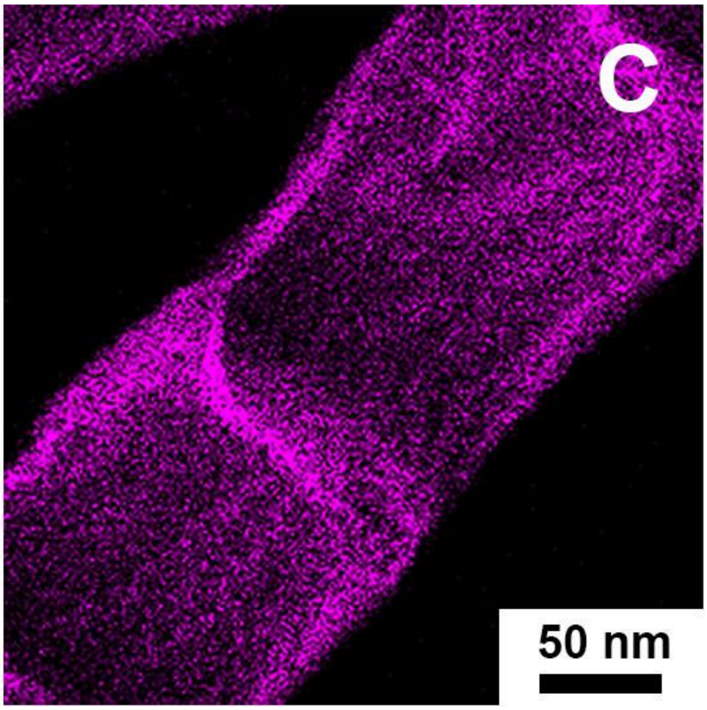

Supplementary Figure 2. EDS elemental map of CoN@CNTs, showing a uniform distribution of C on the tubular structure.

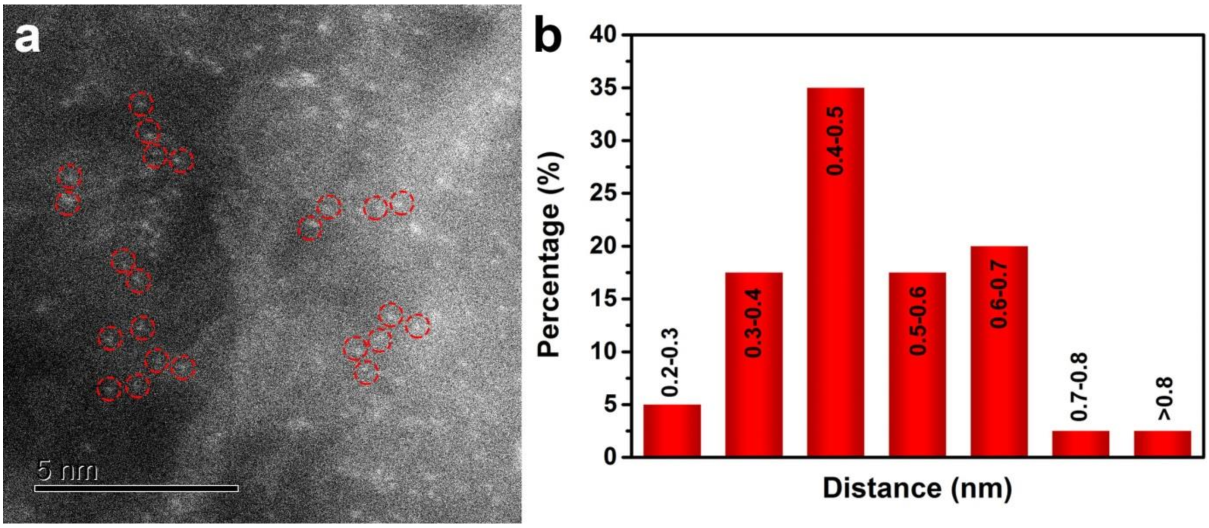

Supplementary Figure 3. (**a**) STEM image of a selected area on the CoN@CNTs, showing the atomic distribution of Co atoms on the carbon layers. (**b**) The distribution of distances of adjacent cobalt atoms counted from 50 neighbouring metal atom pairs. The red circles in Supplementary Figure 3 are giving examples of single atom pairs used to investigate the atomic distances between adjacent Co atoms. Sampling of distances of metal atom pairs from other STEM images was also adopted, they are not presented here for the sake of brevity.

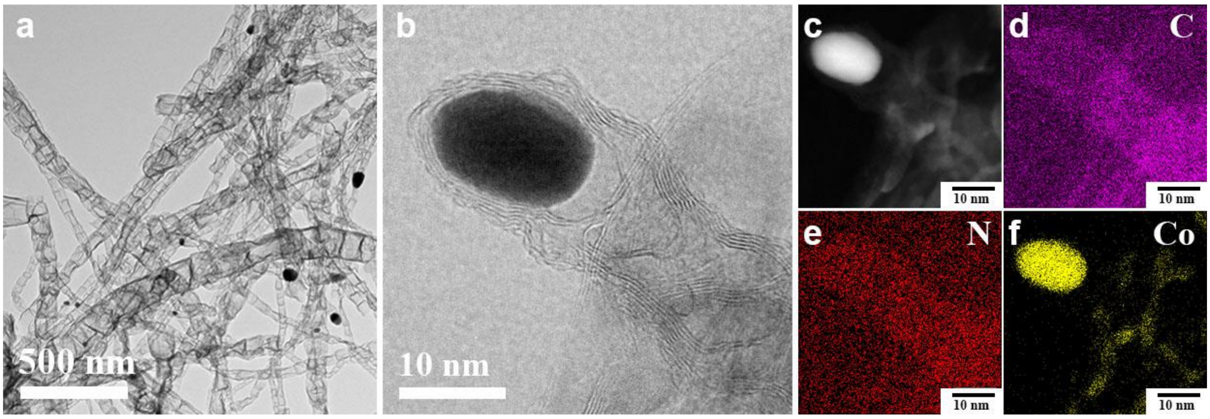

Supplementary Figure 4. (**a**) TEM (**b**) HR-TEM and (**c**) STEM image of the CoN@CNTs composite and the correlating EDS maps of (**d**) C, (**e**) N and (**f**) Co. The images in Supplementary Figure 4 reveal the existence of cobalt nanoparticles encapsulated in 5 to 7 layers of carbon at the enclosed end of CNTs.

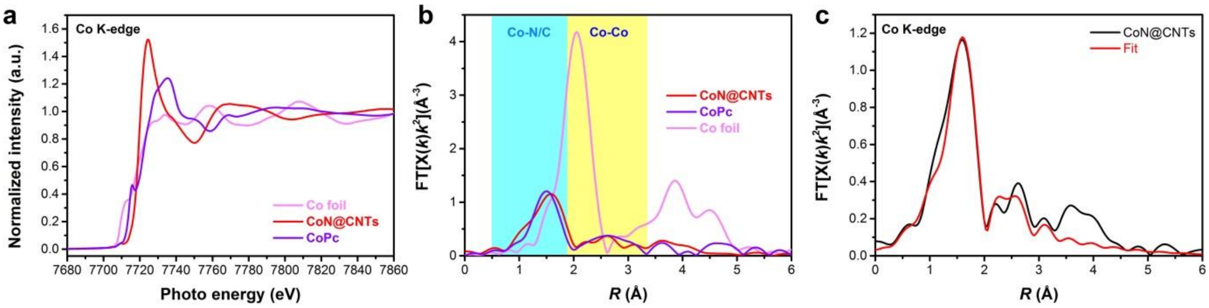

Supplementary Figure 5. The Co K-edge (**a**) XANES spectra and (**b**) FT-EXAFS spectra of the CoN@CNTs composite, CoPc and Co foil. (**c**) FT-EXAFS curves-fitting analysis of the CoN@CNTs composite. Measured and calculated spectra are matched very well for the CoN@CNTs sample. The best-fit parameters are shown in Supplementary Table 1. The weak signals at ~2.3 to 2.7 Å in the Co K-edge EXAFS of CoN@CNTs might be ascribed to the Co-Co scattering path originated from a metallic feature in the Co nanoparticles wrapped at the closed end of carbon nanotubes (Supplementary Figure 4)[1-3]. Compared with the obvious Co-N scattering feature (at ~1.5 Å), the weak signals of Co-Co bond suggest a very rare appearance of metallic cobalt nanoparticles in the CoN@CNTs samples, as evidenced from TEM images (Supplementary Figure 1 and 4).

Supplementary Figures on the catalytic performances and surface physical properties on catalyst:

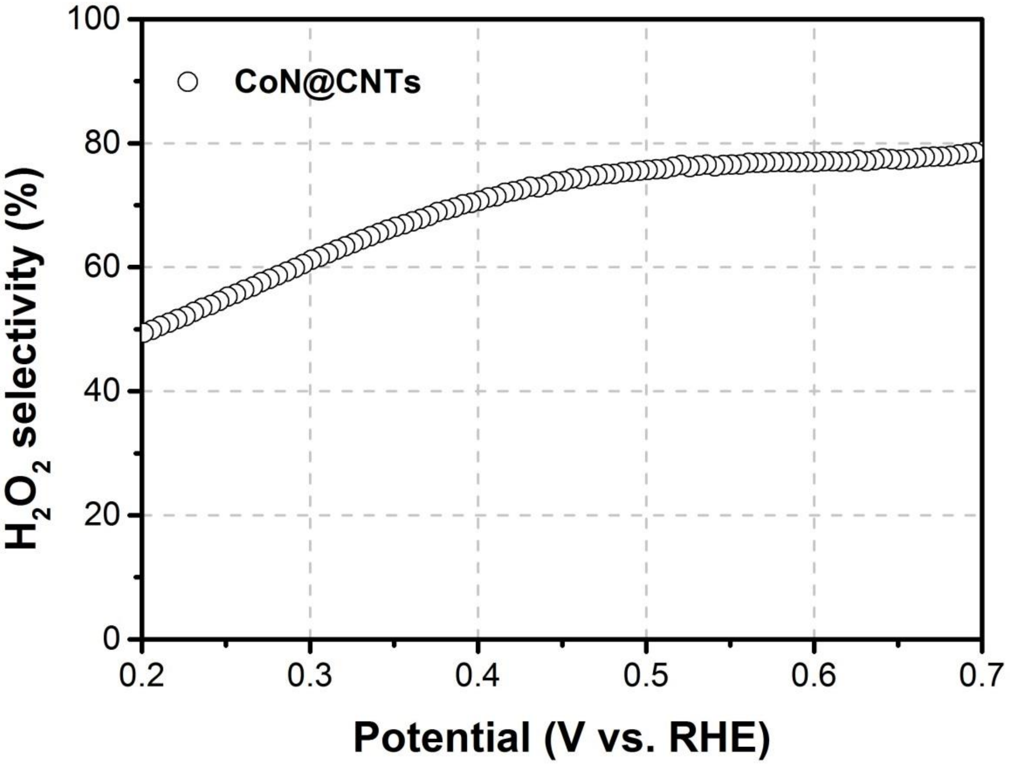

Supplementary Figure 6. Calculated H_2O_2 selectivity on CoN@CNTs based on the RRDE measurements.

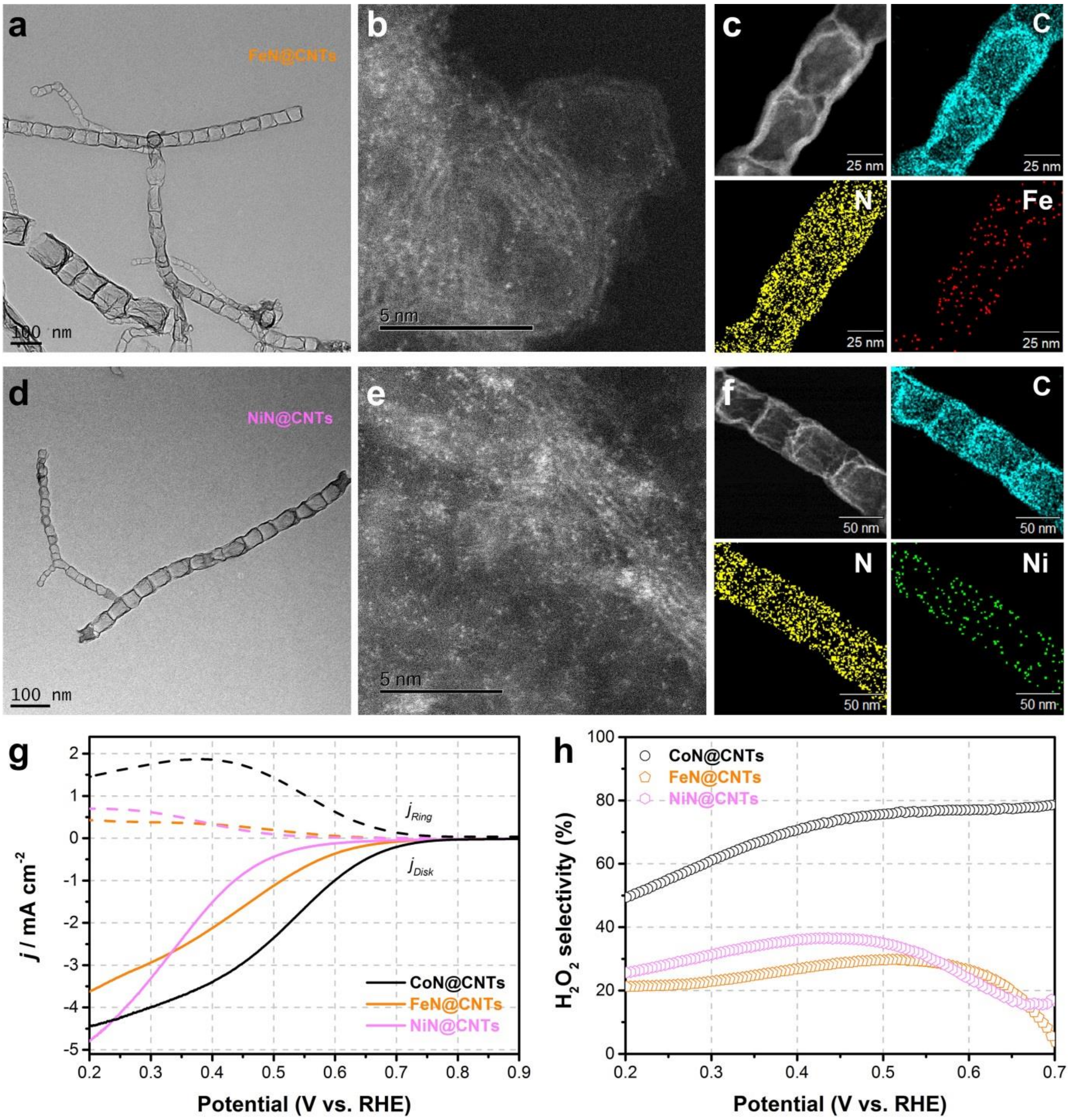

Supplementary Figure 7. TEM images of the (**a**) FeN@CNTs and (**d**) NiN@CNTs composite. HAADF-STEM images of (**b**) FeN@CNTs and (**e**) NiN@CNTs, showing the isolated distribution of Fe and Ni single atoms (bright dots). HAADF–STEM images and corresponding EDS maps of (**c**) FeN@CNTs and (**f**) NiN@CNTs for C, N, Fe and Ni. (**g**) RRDE voltammograms of CoN@CNTs, FeN@CNTs and NiN@CNTs at 1600 rpm in an O_2-saturated 0.1 M $HClO_4$ electrolyte with disc current and ring current. (**h**) Calculated H_2O_2 selectivity on CoN@CNTs, FeN@CNTs and NiN@CNTs based on the RRDE measurements.

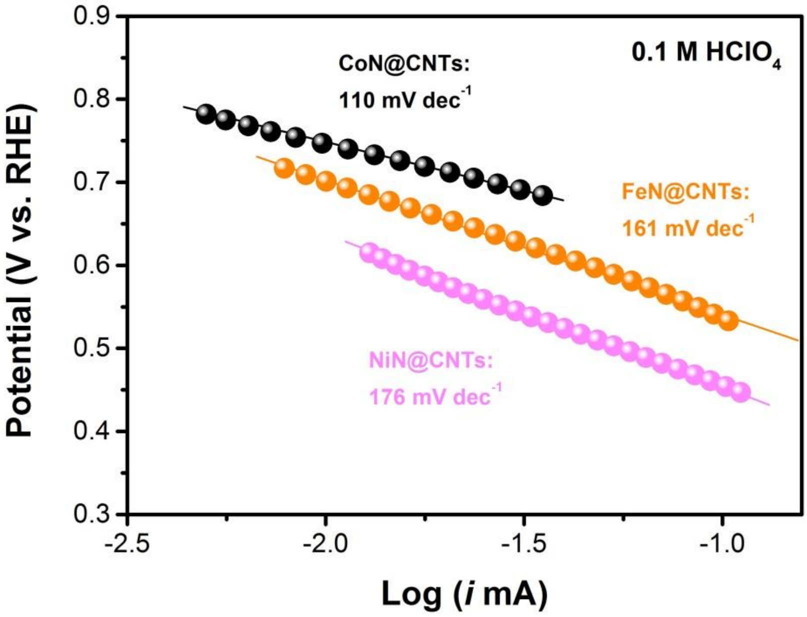

Supplementary Figure 8. Tafel plots of the CoN@CNTs, FeN@CNTs and NiN@CNTs composites based on RRDE measurements.

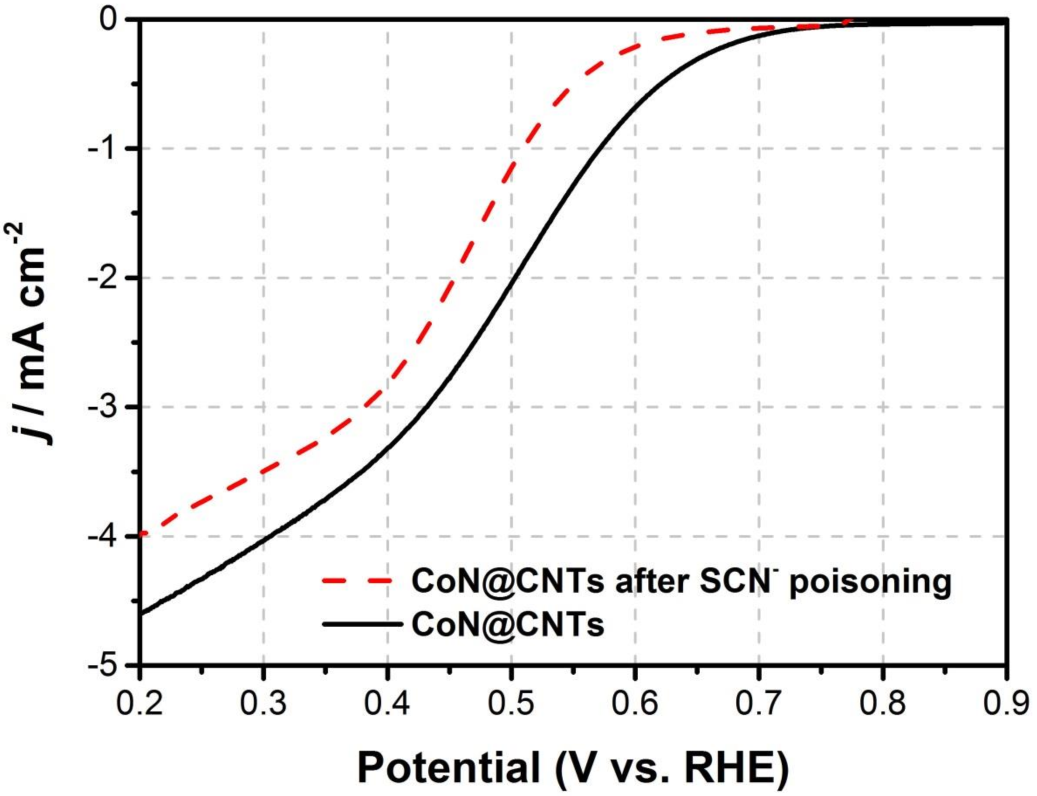

Supplementary Figure 9. ORR polarization curves of CoN@CNTs before and after adding 5 mM SCN^- ions into the 0.1 M $HClO_4$ electrolyte. During the whole process, the solution is purged with O_2 continuously. Due to the huge oxidation current of SCN^- ions reflected by the Pt ring, the peroxide current cannot be detected.

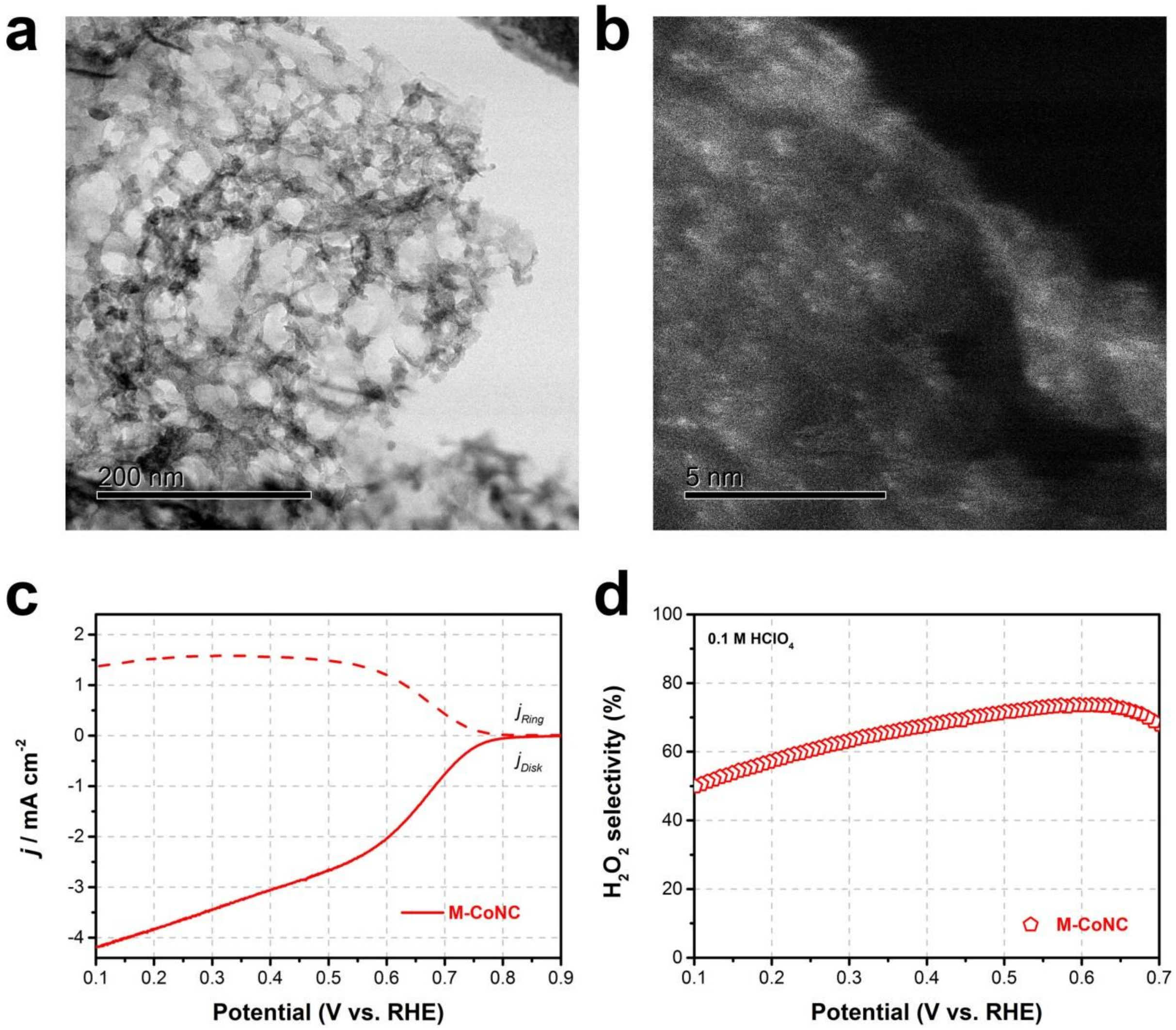

Supplementary Figure 10. (**a**) TEM and (**b**) STEM images of the M-CoNC composite. (**c**) RRDE voltammograms of M-CoNC at 1600 rpm in an O_2-saturated 0.1 M $HClO_4$ electrolyte with disc current and ring current. (**d**) Calculated H_2O_2 selectivity on M-CoNC based on the RRDE measurements. Supplementary Figure 10 shows that M-CoNC prepared via a different method contains single Co atoms only. Besides, judging from the RRDE results, a 2-electron-transfer pathway is dominating the O_2 reduction process on the M-CoNC, confirming the important role of Co-N_x species in catalysing the H_2O_2 production via ORR and further precluding the possible effect of a Co/C core-shell structure in promoting the H_2O_2 synthesis.

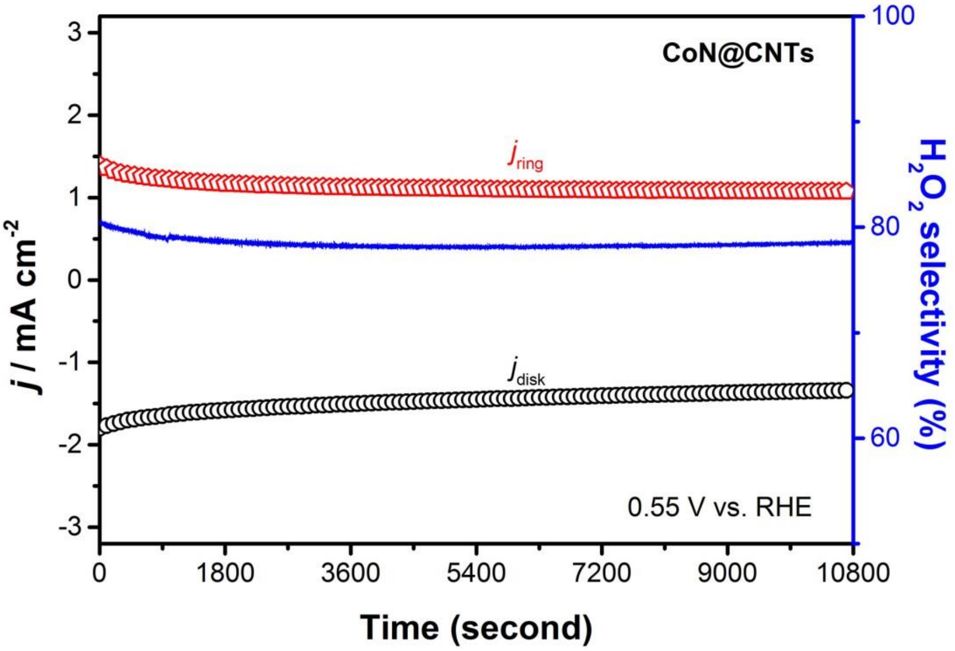

Supplementary Figure 11. The chronoamperometry performed using the RRDE system with the glassy carbon disk and Pt ring held at 0.55 and 1.2 V vs. RHE, respectively, in 0.1 M $HClO_4$. The peroxide current (red curve) on the Pt ring (j_{ring}) was corrected by the collection efficiency. The H_2O_2 selectivity (blue curve) was calculated from the RRDE measurements. Based on the observations from Supplementary Figure 11, the ORR activity (reflected by j_{disk}) on the CoN@CNTs was maintained constantly within 3 hour, and the H_2O_2 selectivity is kept around 78% during the whole process.

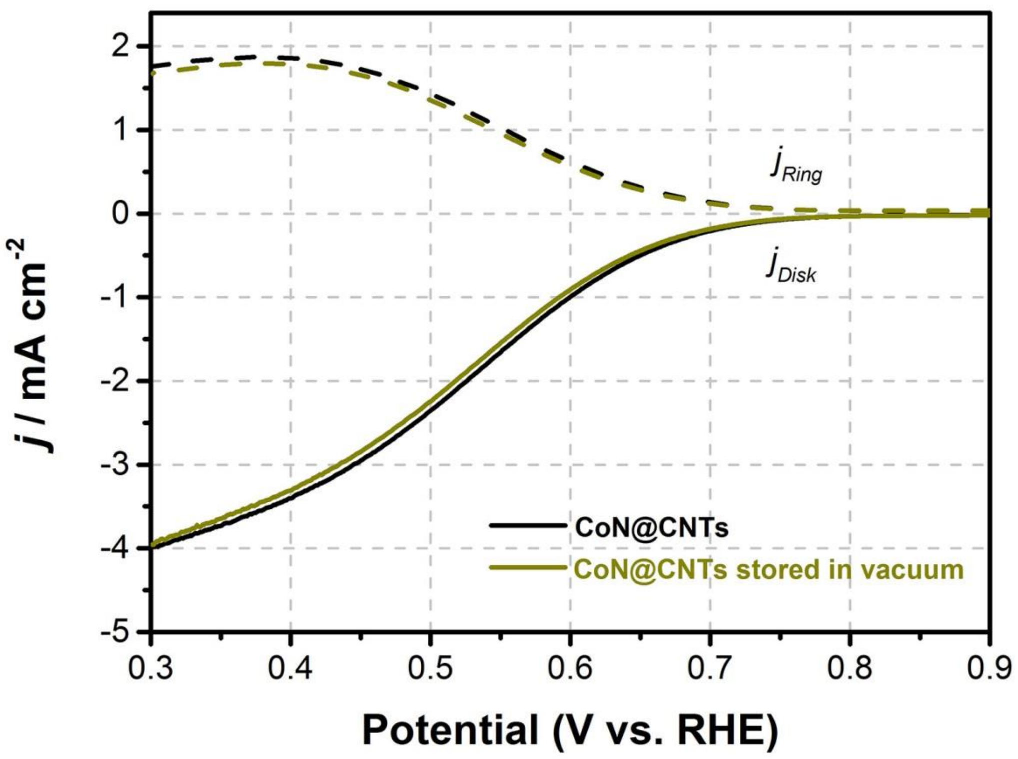

Supplementary Figure 12. ORR polarization curves of fresh CoN@CNTs and sample stored in vacuum for one month in 0.1 M $HClO_4$ electrolyte. During the whole process, the solution is purged with O_2 continuously.

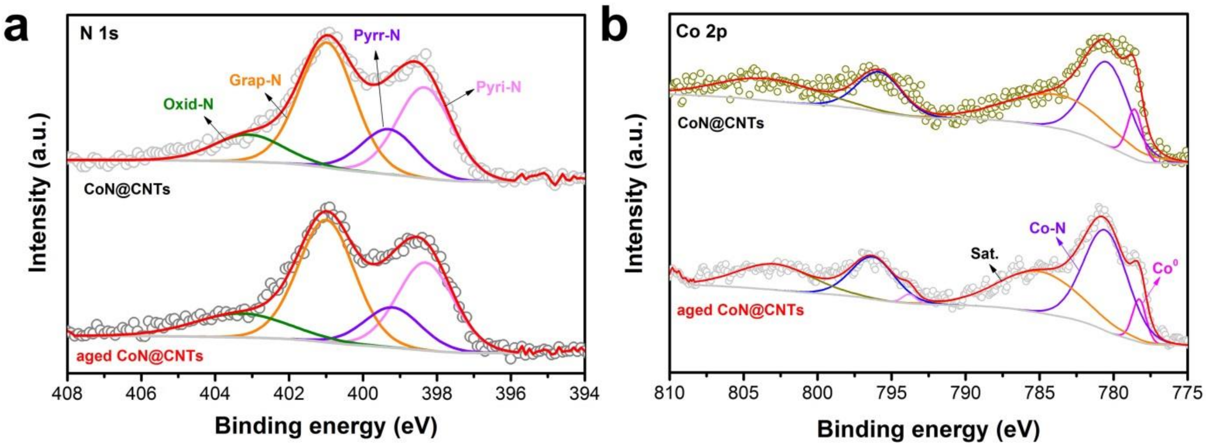

Supplementary Figure 13. The XPS (**a**) N 1s and (**b**) Co 2p spectra of the fresh and aged CoN@CNTs composite.

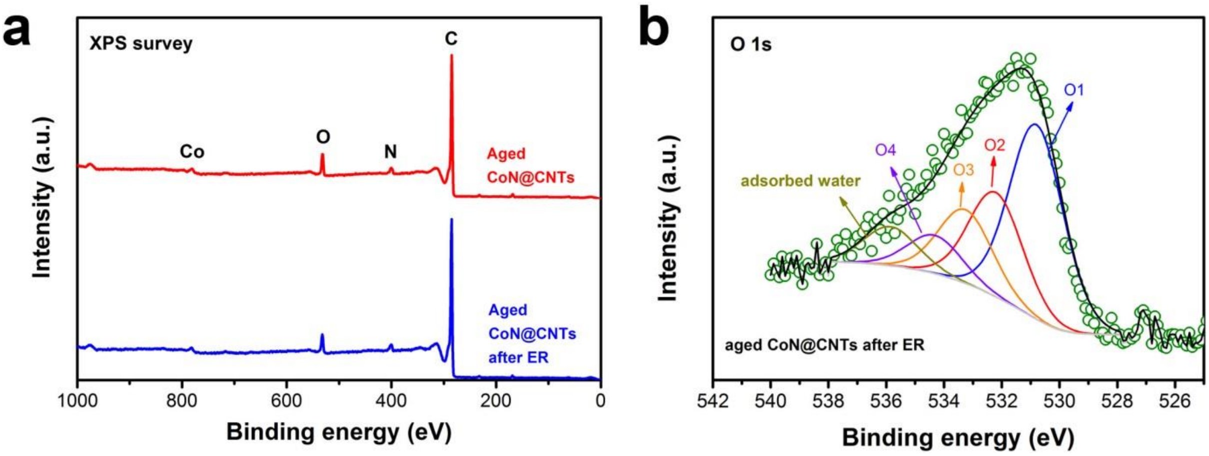

Supplementary Figure 14. (**a**) The XPS elemental survey and (**b**) O 1s spectrum of the aged CoN@CNTs after electrochemical reduction (ER) process. The appearance of the adsorbed water might be due to the water molecules attached on the surface of catalysts after performing ER process in acidic electrolyte. Compared with the aged CoN@CNTs, the dominant emergence of ketonic O (O1) in the CoN@CNTs is kept unchanged after ER process, suggesting that simply doing electrochemical reduction is hard to influence the oxygen environment on the surface of the catalysts.

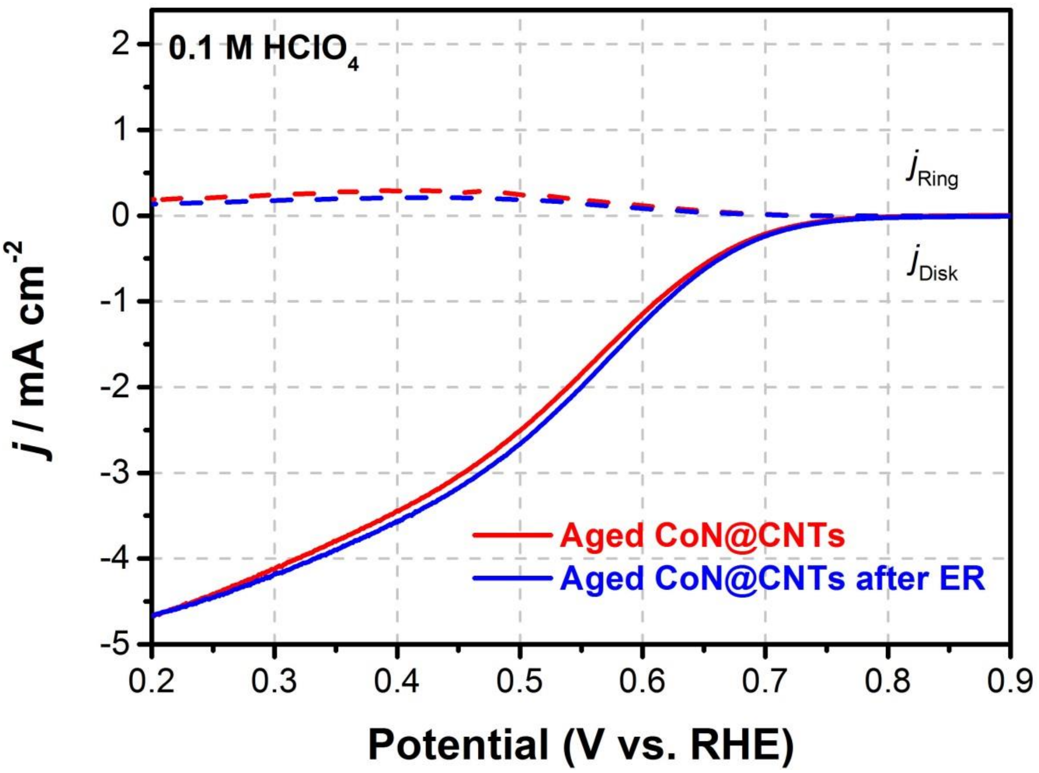

Supplementary Figure 15. RRDE voltammograms of the aged CoN@CNTs before and after electrochemical reduction treatment (ER) at 1600 rpm in an O_2-saturated 0.1 M $HClO_4$ electrolyte with disc current and ring current. The electrochemical reduction process was conducted by performing the linear scan voltammetry (LSV) from 1 to -0.5 V vs. RHE.

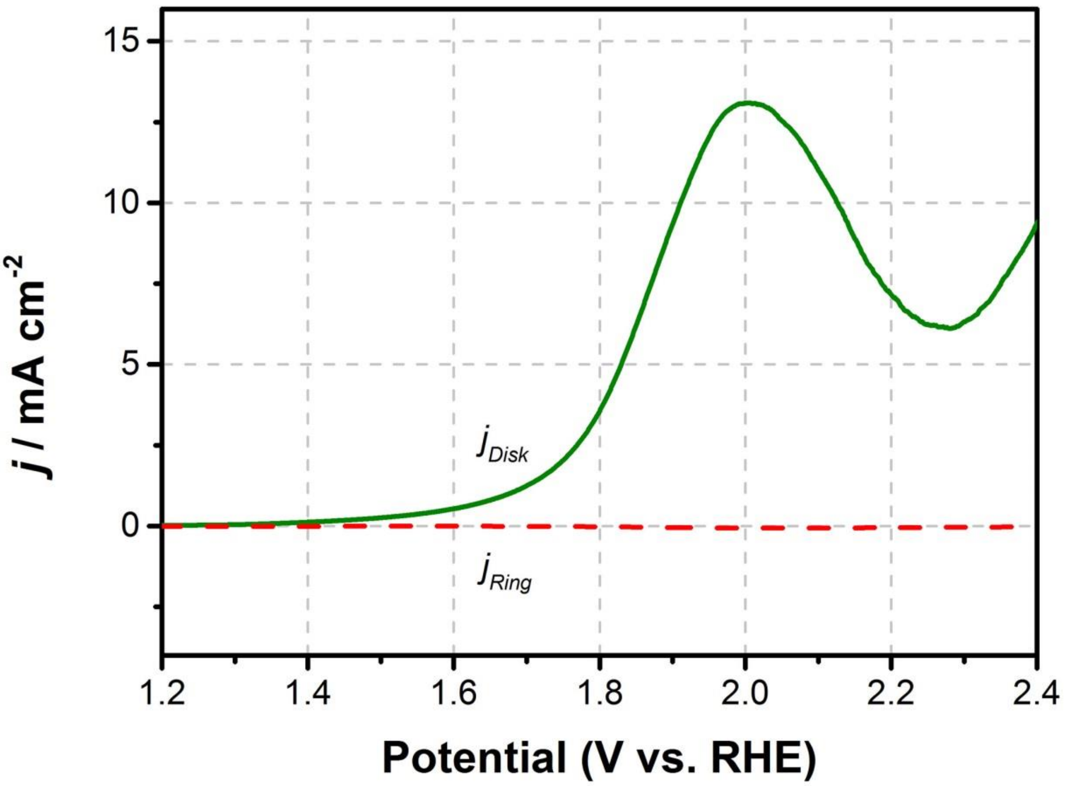

Supplementary Figure 16. Polarization curves showing the electrochemical oxidation applied on the CoN@CNTs. The Pt ring is held at 0.2 V during the oxidation process to detect the possible O_2 evolved from oxidation process. Specifically, the aged CoN@CNTs was electrochemically oxidized by conducting an anodic linear scan voltammetry (from 1.2 to 2.4 V vs. RHE) in the 0.1 M $HClO_4$ solution, during which an anodic peak appears at ~2 V and no oxygen evolution can be detected at Pt ring, indicating the anodic peak is related to a surface oxidation process on CoN@CNTs rather than oxygen evolution.

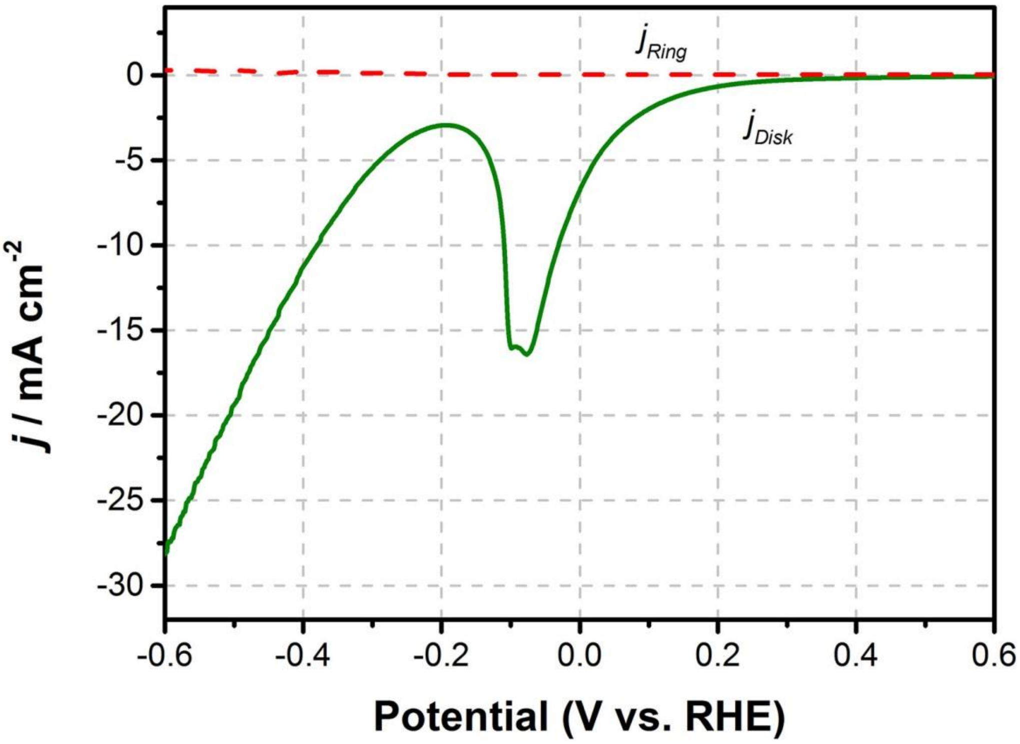

Supplementary Figure 17. Polarization curves showing the reduction process applied on the CoN@CNTs. The ring is held at 1.2 V during the reduction process to detect the possible H_2O_2 formed from ORR reduction process. Specifically, the aged CoN@CNTs was electrochemically reduced through performing a cathodic linear scan voltammetry (from 0.6 to -0.6 V vs. RHE) in the 0.1 M $HClO_4$ solution, during which a cathodic peak appears at ~-0.1 V and no H_2O_2 can be detected at Pt ring, suggesting the cathodic peak might be related to a surface reduction process on CoN@CNTs.

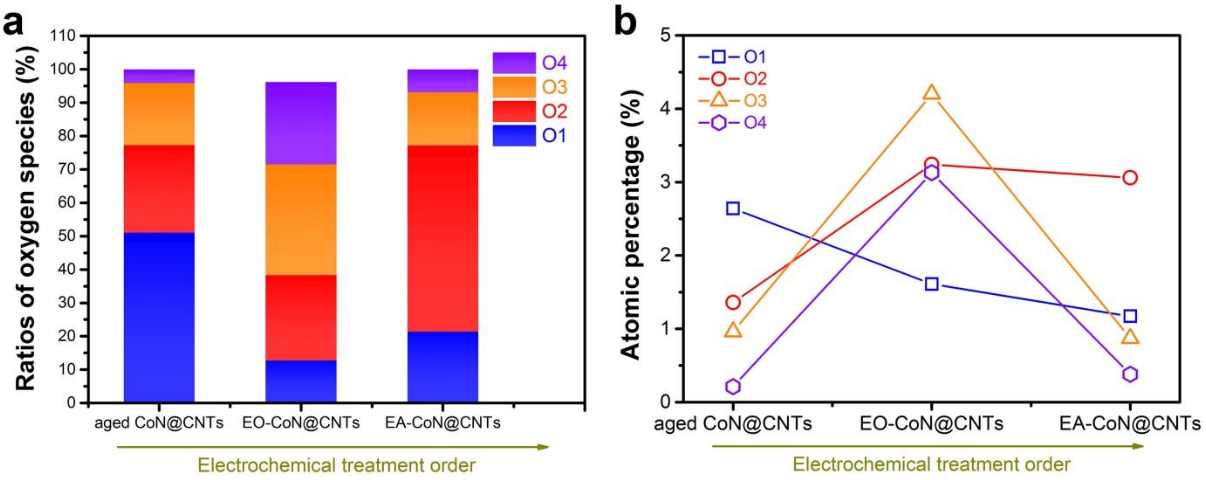

Supplementary Figure 18. (**a**) The ratios of different oxygen species within the total amount of oxygen in the aged CoN@CNTs, EO-CoN@CNTs and EA-CoN@CNTs powders. (**b**) The atomic percentages of different oxygen functional groups within the aged CoN@CNTs, EO-CoN@CNTs and EA-CoN@CNTs powders. All these results were obtained by XPS O 1s measurements and analysis, details are shown in Supplementary Table 3.

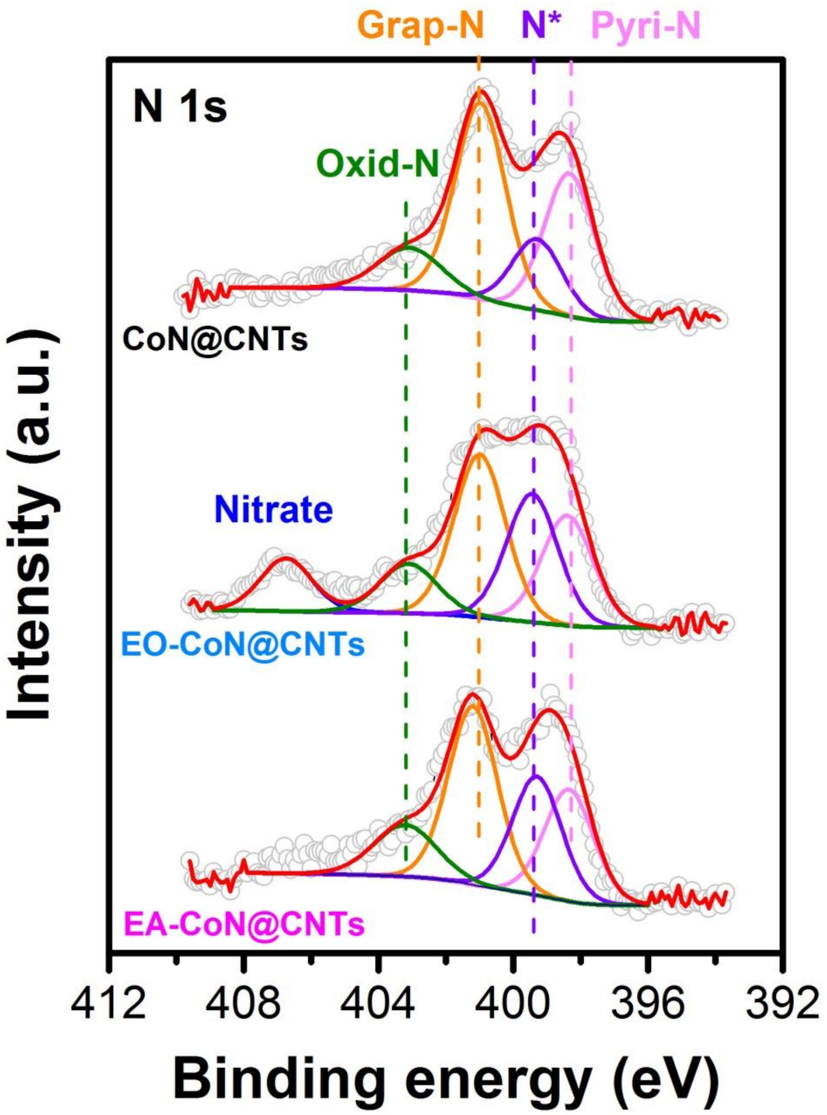

Supplementary Figure 19. XPS N 1s spectra of CoN@CNTs before and after a series of ETs. The peaks locating at 398.4±0.2 eV, 399.3±0.2 eV, 401.1±0.2 eV and 403.2±0.2 eV could be correlated to the pyridinic N, N*, graphitic N and oxidized N. The N* peak with a ~1 eV upshift from the pristine pyridinic N could be correlated to (1) pyrrolic N or (2) pyridonic N[4].

Compared to CoN@CNTs (15.3%), the electrochemical treatments yield a higher concentration of N component (namely N*) corresponding to the peak at ~399.3 eV on both EO-CoN@CNTs (28.1%) and EA-CoN@CNTs (25.0%) (Supplementary Figure 19). In contrast, the pyridinic N (398.4±0.2 eV) decreased from 30.8% (aged CoN@CNTs) to 20.6% (EA-CoN@CNTs) after electrochemical treatments, whereas the sum of pyridinic N and N* species remained largely constant (from 46.1 to 48.5%), suggesting a possible conversion

between these two N components during ETs. Generally, the N* peak with a ~1 eV upshift from the pristine pyridinic N could be correlated to either pyrrolic N or pyridonic N[4], and in some cases it corroborates the existence of Co-bonded N component[5]. In this work, “chemical intuition” suggests that the increased N* species in EO-CoN@CNTs and EA-CoN@CNTs would be pyridonic N, as both Co-bonded N and pyrrolic N are electrochemically stable and hard to be generated by simple ETs[6]. Thus, the difference in the composition of nitrogen species before and after ETs suggests that carbon atoms next to pyridinic N tend to bond with oxygen functionalities generated via ETs with consequent transformation of pyridinic N to pyridonic N[4]. By combining these observations above, it is plausible to postulate that the epoxy O produced through ETs would bond to carbon atoms near pyridinic N that possibly coordinate with Co atoms in EA-CoN@CNTs.

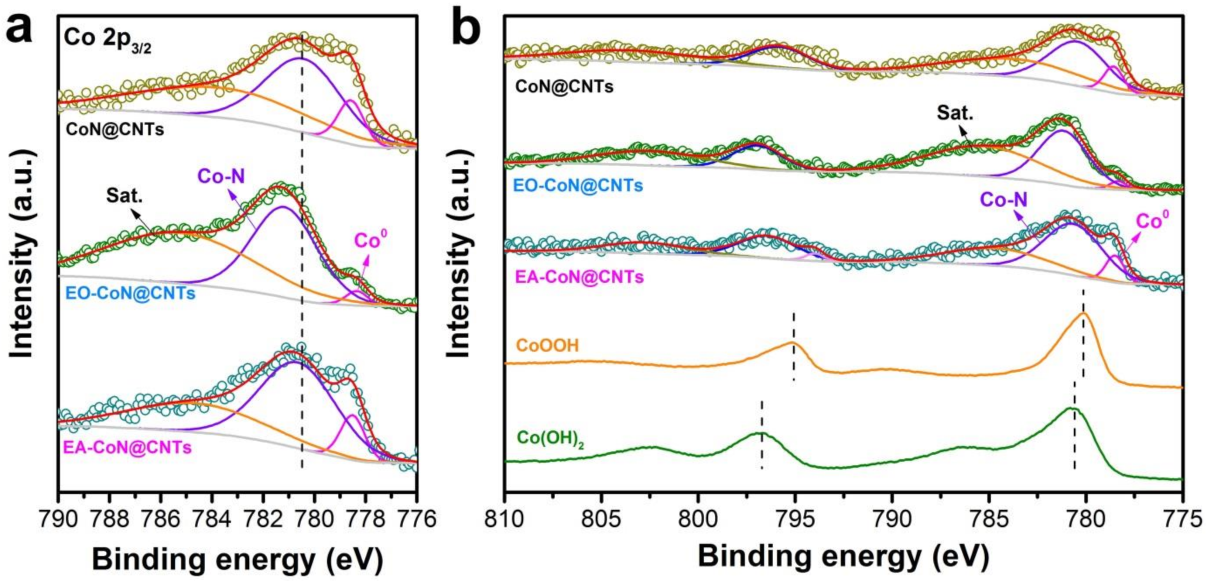

Supplementary Figure 20. XPS Co 2p spectra of CoN@CNTs before and after ETs. (**a**) Co $2p_{3/2}$ spectra of CoN@CNTs before and after ETs exhibit an obvious shift of Co-N peak upon the ETs, suggesting an interaction between oxygen functional groups formed via ETs and Co-N_x centers. (**b**) Co 2p spectra showing a nearly unchanged spin-energy separation and Co^0 position after ETs. For comparison purpose, CoOOH (Co^{3+}) and $Co(OH)_2$ (Co^{2+}) were also measured to evaluate the valence state of Co in the CoN@CNTs.

High resolution XPS Co $2p_{3/2}$ spectra presented in Supplementary Figure 20a exhibit characteristic peaks of the Co^0 and N-coordinated Co (Co-N_x) in all three samples. Notably, compared with CoN@CNTs, the Co-N_x peak of EO-CoN@CNTs (~781.1 eV) shifts positively toward the higher binding energy side, while that of EA-CoN@CNTs shifts back to a lower binding energy position (~780.7 eV) which is still higher than the CoN@CNTs (~780.4 eV). These significant shifts of binding energy for the Co-N_x peak on electro-modified samples demonstrate that the electrochemical re-construction of oxygen functional groups might happen around those Co-N_x centers, and that in turn changes the electronic state of Co atoms by an electron-withdrawing effect of oxygen species. In contrast, the spin-energy separation (Supplementary Figure 20b) and Co^0 peak in all three samples remain at the same value (15.7±0.2 eV) and position (~778.5 eV), respectively, suggesting the valence states of positively charged Co in Co-N_x species were not influenced by the electrochemical treatments.

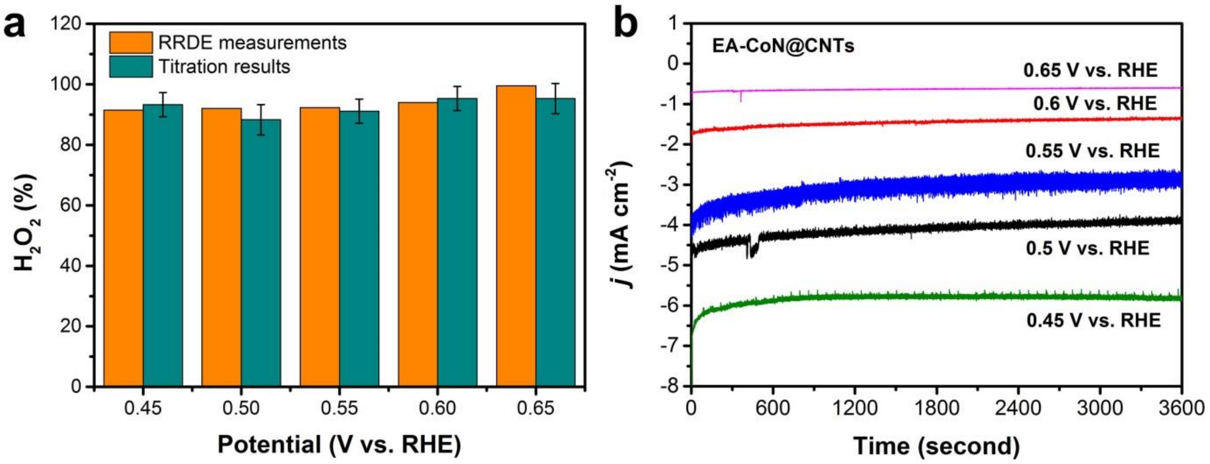

Supplementary Figure 21. (**a**) The H_2O_2 selectivity obtained from both RRDE measurements and chemical titration for the electro-activated CoN@CNTs in an O_2-saturated 0.1 M $HClO_4$ electrolyte. (**b**) *i*-t curves obtained from the H_2O_2 bulk production on the electro-activated carbon fibre paper electrode loaded with EA-CoN@CNTs under different operation potentials for the chemical titration.

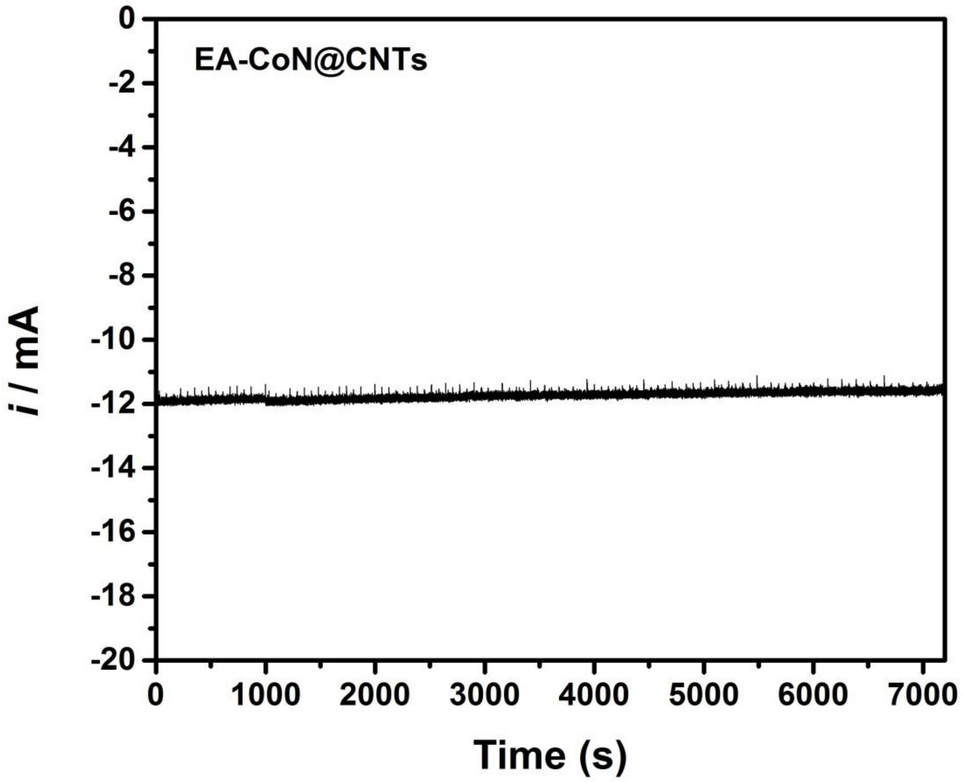

Supplementary Figure 22. The chronoamperometric measurement at 0.45 V for 2 h after changing the electrolyte and rinsing the electrode with deionized water. The EA-CoN@CNTs modified electrode after a 12 h chronoamperometric testing at 0.45 V was employed as working electrode here. Supplementary Figure 22 shows that the ORR activity on the EA-CoN@CNTs has been totally recovered.

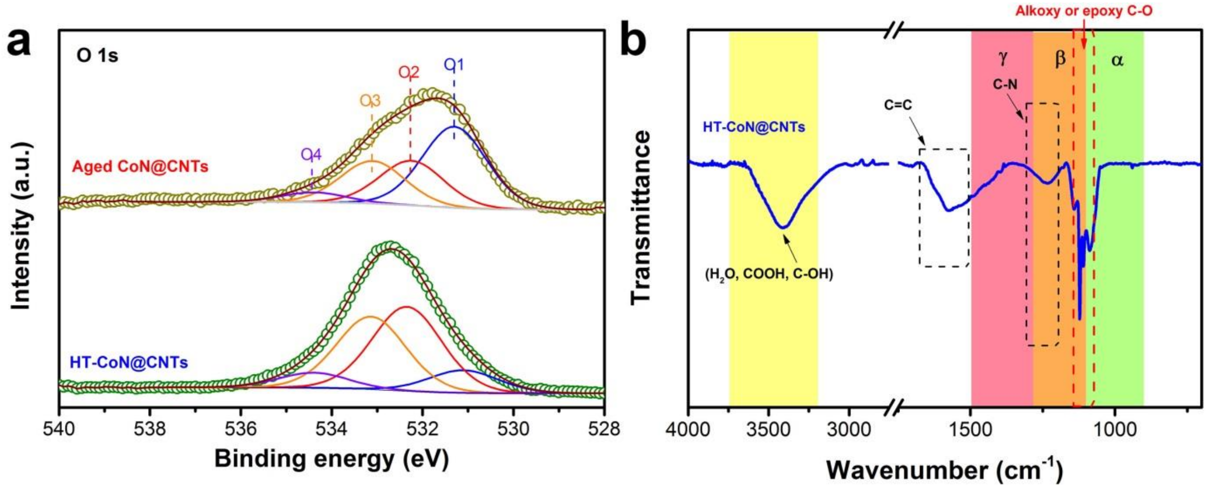

Supplementary Figure 23. (**a**) XPS O1s spectra of the aged CoN@CNTs and HT-CoN@CNTs, showing an apparent emergence of epoxy groups on CoN@CNTs after H_2O_2 treatment. (**b**) Background-corrected FTIR spectrum of HT-CoN@CNTs.

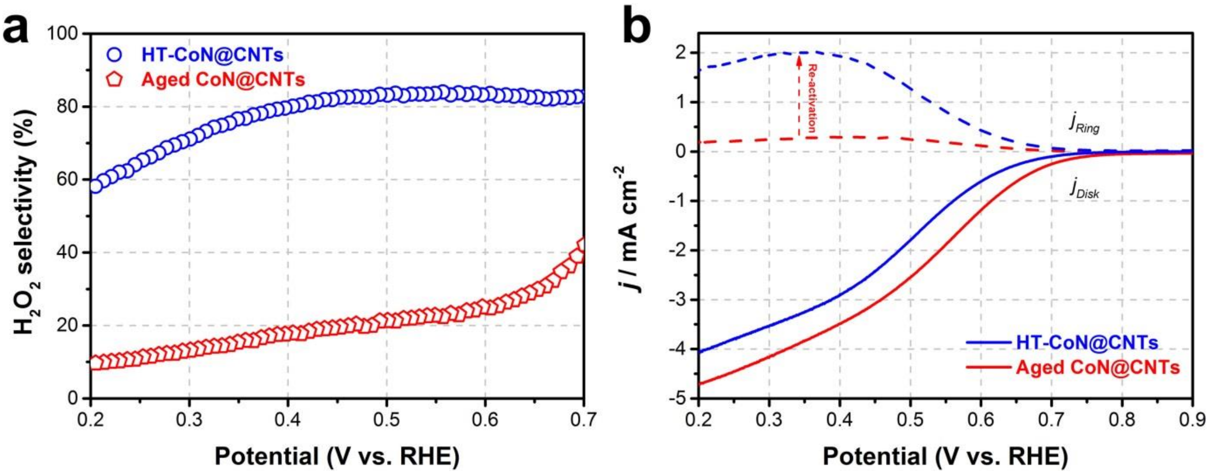

Supplementary Figure 24. (**a**) The calculated H_2O_2 selectivity of HT-CoN@CNTs and aged CoN@CNTs from the RRDE measurements. (**b**) RRDE voltammograms of the aged CoN@CNTs before and after H_2O_2 treatment (HT) at 1600 rpm in an O_2-saturated 0.1 M $HClO_4$ electrolyte with disc current and ring current.

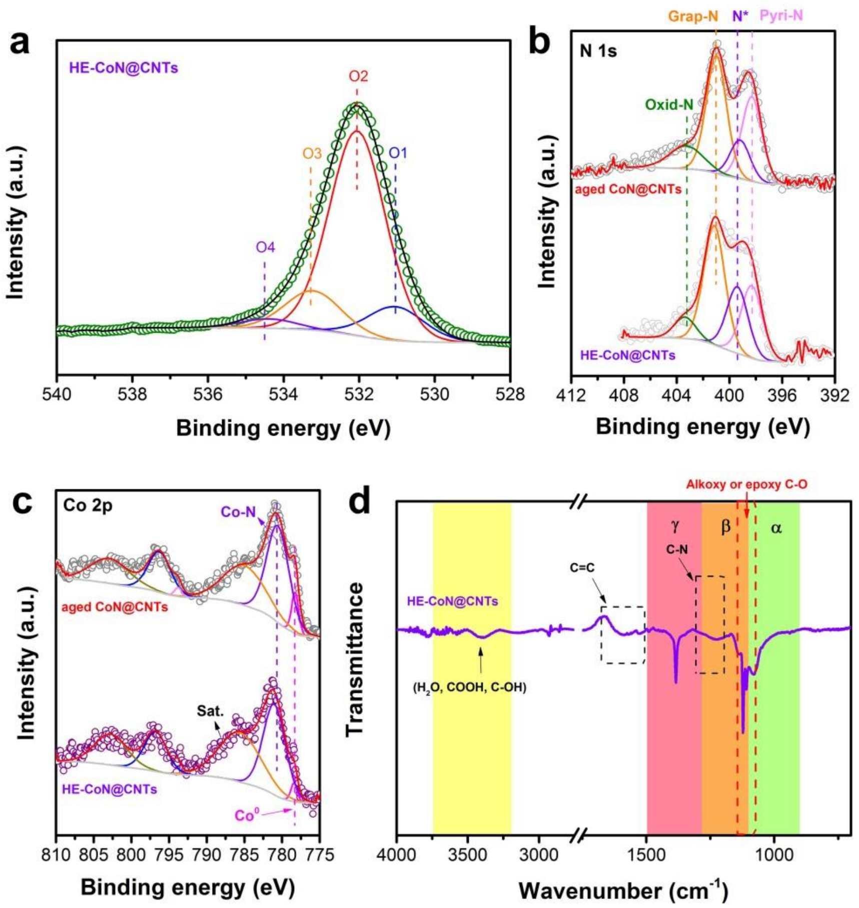

Supplementary Figure 25. (**a**) XPS O 1s spectrum of HE-CoN@CNTs, showing an apparent emergence of epoxy groups on CoN@CNTs after both H_2O_2 and electrochemical treatment. (**b**) XPS N 1s spectra of the aged CoN@CNTs and HE-CoN@CNTs. (**c**) XPS Co 2p spectra of the aged CoN@CNTs and HE-CoN@CNTs. (**d**) Background-corrected FTIR spectrum of HE-CoN@CNTs.

Compared to both CoN@CNTs and aged CoN@CNTs (Supplementary Figure 13), the HE-CoN@CNTs exhibits a significantly higher ratio of N component located at ~399. 3 eV (namely N*), corroborating well the formation of more pyridonic N that might be converted from the pyridinic N near the HE-generated epoxy groups. Co 2p spectrum of HE-CoN@CNTs also reveals a slightly positive shift (~0.4 eV) of binding energy of Co-N peak, suggesting an interaction between the HE-generated epoxy groups and Co-N_x species via a possible electron-withdrawing effect. In the FTIR spectrum of HE-CoN@CNTs (Supplementary Figure 25d), the much receded intensity of a peak at ~3500 cm^{-1} compared to HT-CoN@CNTs (Supplementary Figure 23b) indicates some oxygen functional groups (e.g. C-OOH, C-OH) are easier to be removed by electrochemical reduction method than the epoxy oxygen, and these functionalities should have no correlations with the enhanced H_2O_2 selectivity.

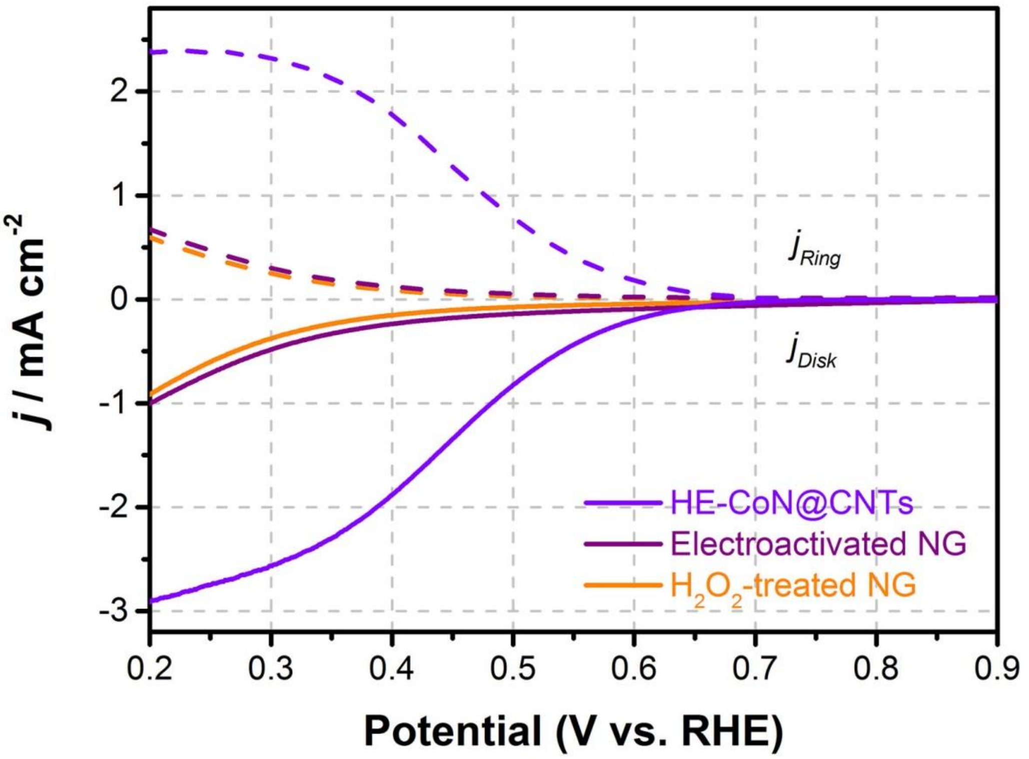

Supplementary Figure 26. Polarization curves of HE-CoN@CNTs and NG after different treatments obtained on RRDE in O_2-saturated 0.1 M $HClO_4$. H_2O_2 and electrochemical treatments are applied on NG to obtain the H_2O_2-treated NG and electroactivated NG, respectively.

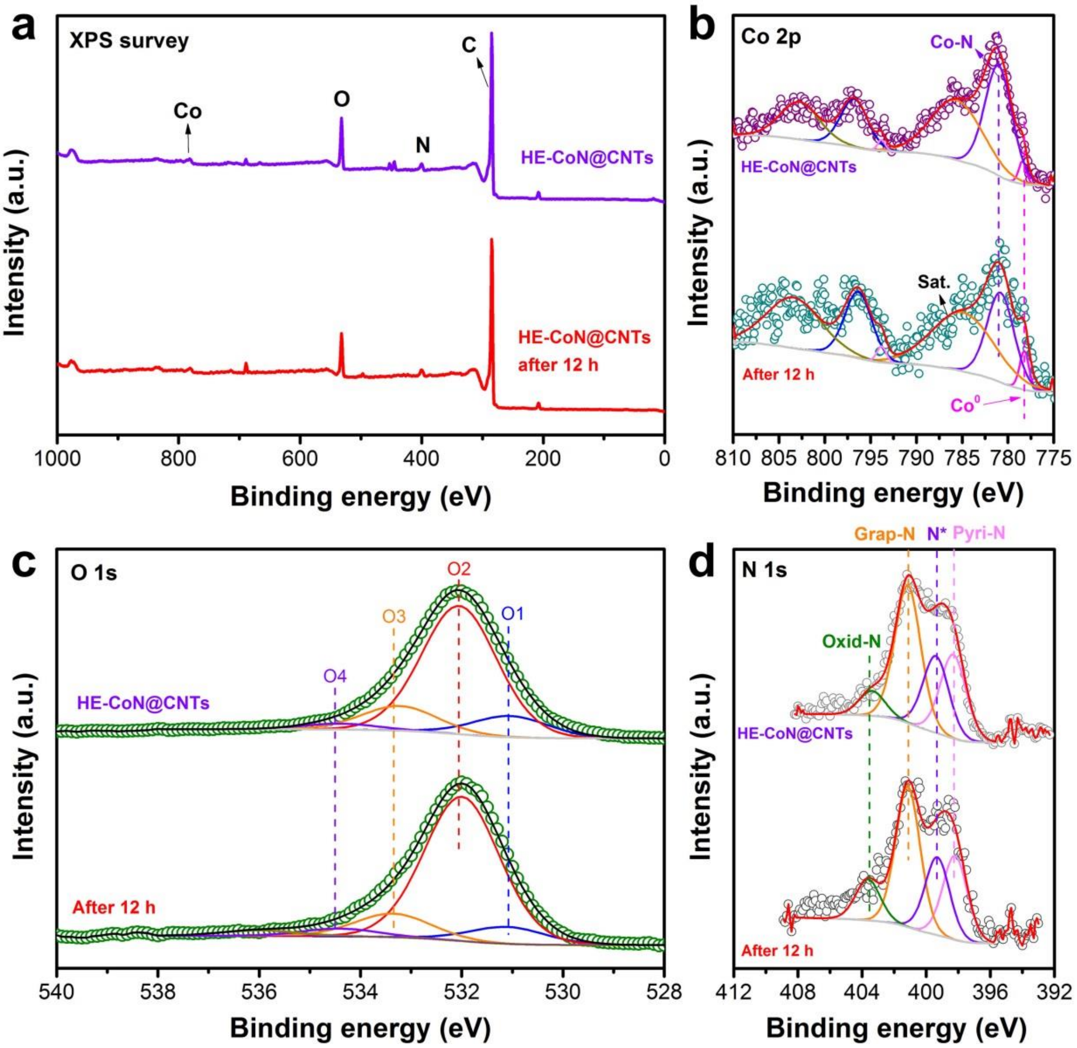

Supplementary Figure 27. (**a**) XPS elemental survey of the HE-CoN@CNTs sample before and after 12-hour testing session for O_2 reduction in 0.1 M $HClO_4$. High resolution XPS (**b**) Co 2p spectra, (**c**) O 1s and (**d**) N 1s of the HE-CoN@CNTs sample before and after 12-hour testing session for O_2 reduction in 0.1 M $HClO_4$.

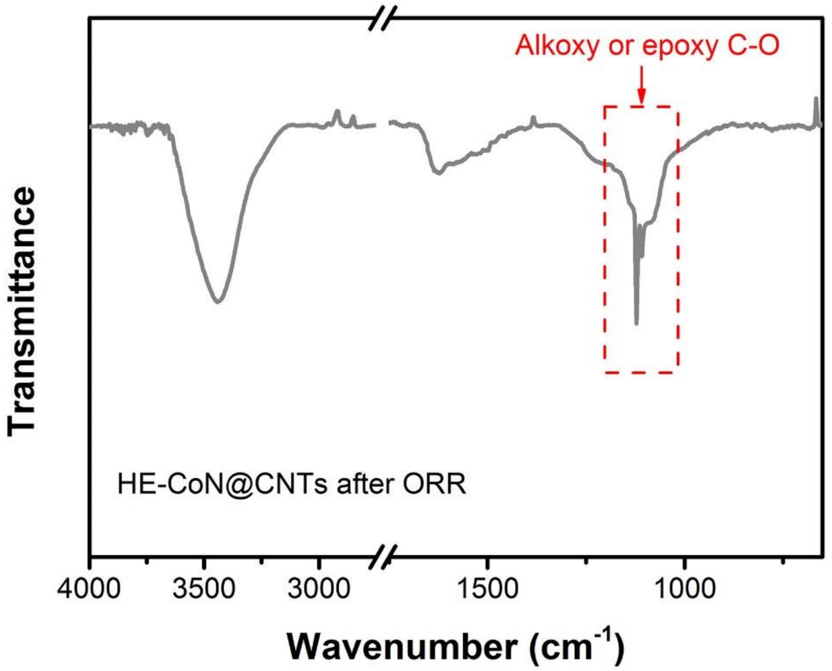

Supplementary Figure 28. Background-corrected FTIR spectrum of the HE-CoN@CNTs sample after 12-hour testing session for O_2 reduction in 0.1 M $HClO_4$, showing obvious emergence of epoxy groups.

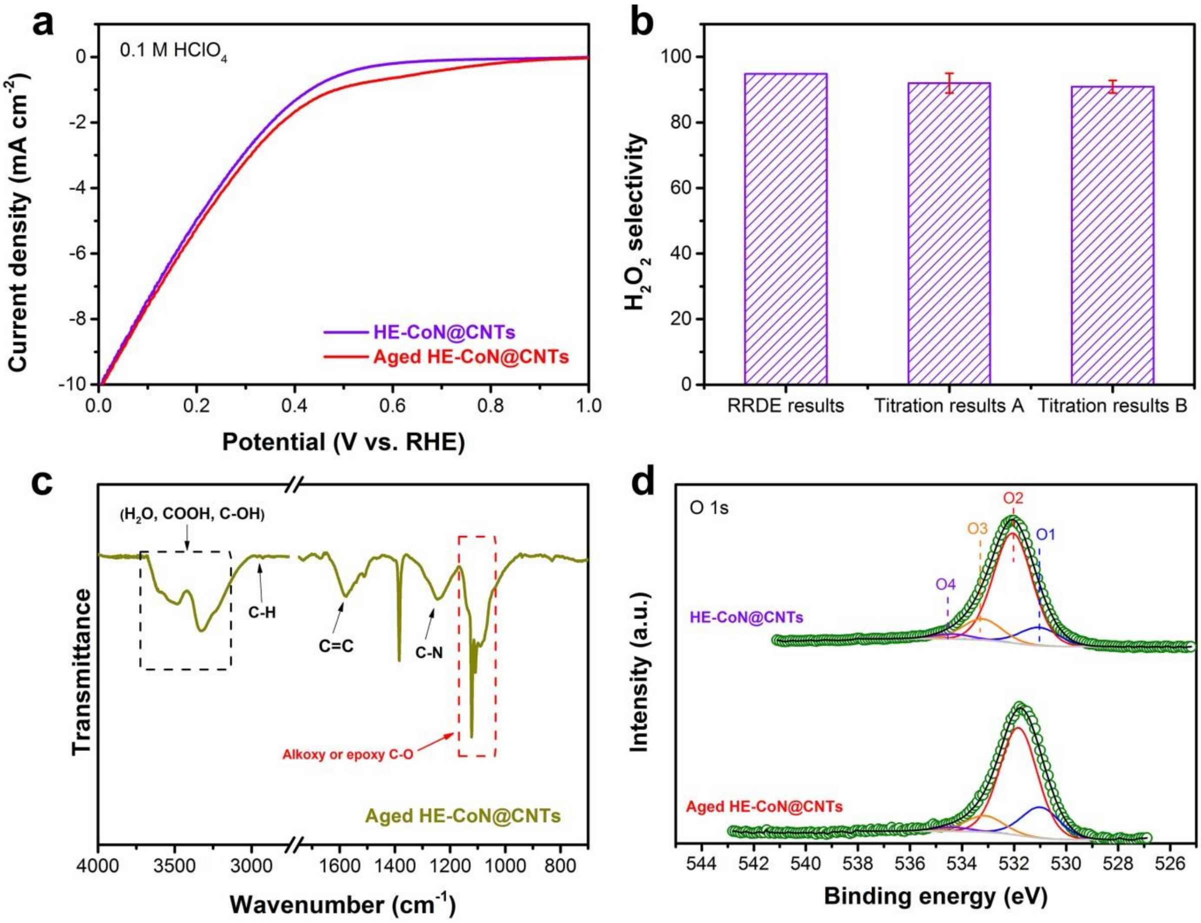

Supplementary Figure 29. (**a**) The polarization curves of HE-CoN@CNTs loaded CFP electrode before and after aging process. (**b**) The H_2O_2 selectivity of HE-CoN@CNTs based on RRDE measurements and chemical titration. The titration results A were obtained from the fresh HE-CoN@CNTs loaded CFP electrode, and the titration results B were obtained from the aged HE-CoN@CNTs loaded CFP electrode. (**c**) Background-corrected FTIR spectrum of aged HE-CoN@CNTs. (**d**) XPS O1 s spectra of HE-CoN@CNTs sample before and after aging in the ambient atmosphere. The lack of obvious increment for C=O groups after aging suggests the emergence of C-O-C groups might occupy the sites that ketonic O would arise (e.g. surface defects) and eventually suppress their significant appearance.

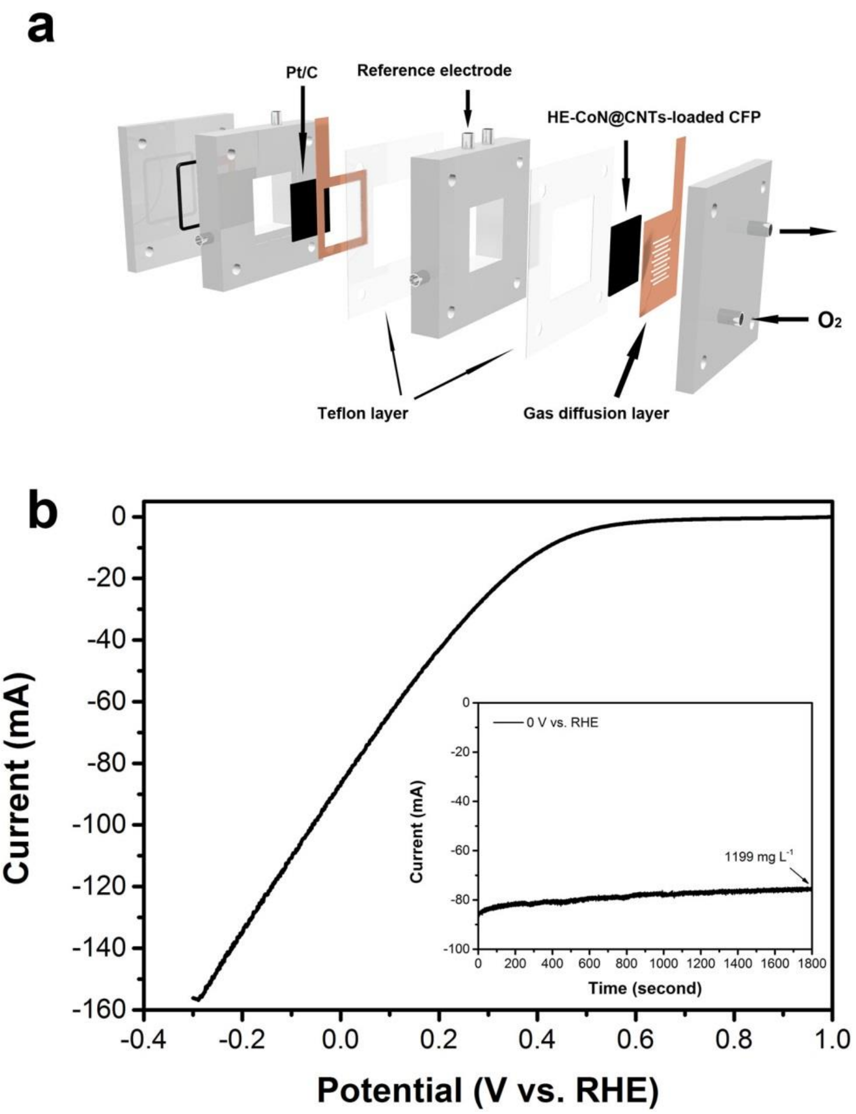

Supplementary Figure 30. (**a**) The schematic illustration of a gas-diffusion electrode (GDE) reactor employed for the bulk H_2O_2 production with continuous O_2 purging in 0.1 M $HClO_4$. A carbon fibre paper (CFP, hydrophobic, 3*3 cm) loaded with HE-CoN@CNTs catalysts (0.24 mg cm^{-2}) was used as working electrode attached to the gas diffusion layer to produce H_2O_2 via ORR. Pt/C-loaded CFP was used as counter electrode. All catalysts (HE-CoN@CNTs or Pt/C) were coated on the left-side of the CFP in Supplementary Figure 30a. (**b**) The ORR polarization curve of the HE-CoN@CNTs obtained within a GDE system in Supplementary Figure 30a. The inset is the chronoamperometric curve of HE-CoN@CNTs loaded GDE obtained at 0 V vs. RHE for H_2O_2 production, giving a current density of ~9 mA cm^{-2} (surface area of CFP = 9 cm^2). The amount of hydrogen peroxide was determined as 1199 mg L^{-1} by a chemical titration method employing the potassium permanganate.

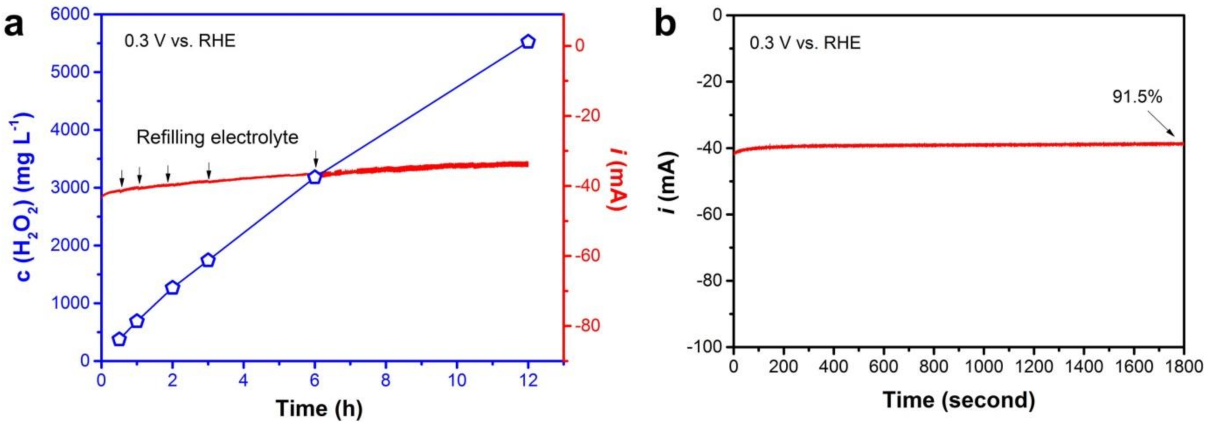

Supplementary Figure 31. (**a**) H_2O_2 production amount (determined via the potassium permanganate titration) as a function of time on the HE-CoN@CNTs loaded CFP in a GDE setup. Current (red curve) and concentration (blue pentagon) behavior with time for the electrochemical H_2O_2 production is shown. The refilling of electrolyte was conducted right after sampling the solution. (**b**) The chronoamperometric measurement on the HE-CoN@CNTs loaded CFP in a GDE set up at 0.3 V for 1800 s after 12-hour H_2O_2 production session in the refreshed electrolyte.

The longer term H_2O_2 production was also performed using the GDE reactor as shown in Supplementary Figure 30 under a more moderate potential (0.3 V_{RHE}) for 12 h (Supplementary Figure 31a), and a current density around 4 mA cm^{-2} was yielded during the whole process. The concentration of H_2O_2 accumulated during the first 30 min was 374 ppm under this moderate operation potential, and it was further increased to around 0.3 wt% (3179 ppm) after 6-hour testing, which is sufficient for many usages in the water treatment industry. Notably, the Faradaic efficiency decreases from ~88% to 68.5 % after 6-hour test , and that is ascribed to the limited mass transport of H_2O_2 near the surface of the working electrode where further H_2O_2 reduction and decomposition could be enhanced under relatively high H_2O_2 concentration. In a RRDE system, H_2O_2 produced at the disk could be rapidly transported away and oxidized at the Pt ring, while this could not be achieved within a carbon fiber paper electrode in a static electrolyte environment. Nevertheless, the decreased current efficiency and ORR activity could be regained by simply rinsing the electrode and changing the electrolyte (Supplementary Figure 31b), indicating the performance loss is not due to the deactivation of catalysts. For future industrial level production of H_2O_2, better cell management and device designs (like a flow cell or trickle bed reactor) are required to avoid the further contact between the generated H_2O_2 and the electrodes.

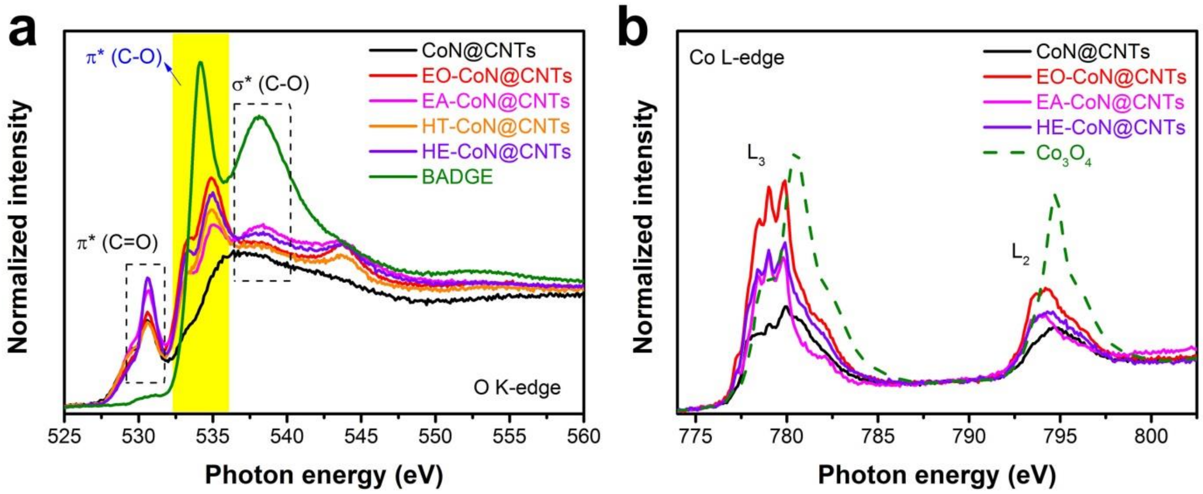

Supplementary Figure 32. (**a**) High resolution O K-edge NEXAFS spectra of CoN@CNTs, EO-CoN@CNTs, EA-CoN@CNTs, HT-CoN@CNTs, HE-CoN@CNTs and bisphenol A diglycidyl ether (BADGE) reference. (**b**) High resolution Co L-edge NEXAFS spectra of CoN@CNTs, EO-CoN@CNTs, EA-CoN@CNTs, HE-CoN@CNTs and Co_3O_4 reference.

Supplementary figures on computational modeling:

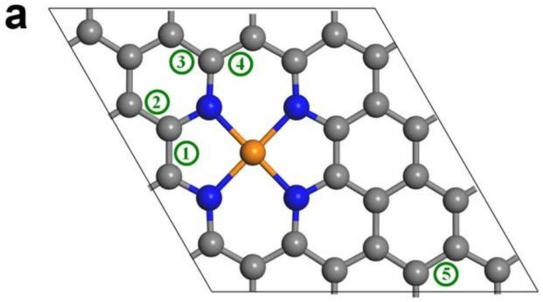

b

Epoxy Oxygen Site	$E^{F}_{\text{ep-O}}$ (eV)	ΔG_{HOO^*} (eV)
1	2.82	4.04
2	2.92	4.07
3	2.96	3.94
4	2.87	3.91
5	3.14	3.99

Supplementary Figure 33. (**a**) The structure of a CoN_4 moiety embedded in a basal graphene plane used in our calculations. The green numbers indicate the possible epoxy group sites considered in this work. The grey, blue and orange balls represent C, N and Co atoms, respectively. (**b**) The calculated $E^{F}_{\text{ep-O}}$ and ΔG_{HOO^*} of CoN_4 moiety with epoxy oxygen at positions as indicated in (**a**). It can be seen that the epoxy oxygen prefer to locate close to CoN_4 moiety because of the less positive $E^{F}_{\text{ep-O}}$.

In order to simulate a CoN_4 moiety embedded in a basal graphene plane, a 4×4 graphene supercell with periodical boundary conditions was used, and then, six carbon atoms was removed to create CoN_4 moiety (Supplementary Figure 33a). The vacuum space was set to larger than 20 Å in the *z* direction to avoid interactions between periodic images. In geometry optimizations, all the atomic coordinates were fully relaxed up to the residual atomic forces smaller than 0.005 eV/Å, and the total energy was converged to 10^{-5} eV. The Brillouin zone integration was performed on the (3×3×1) Monkhorst-Pack k-point mesh[7].

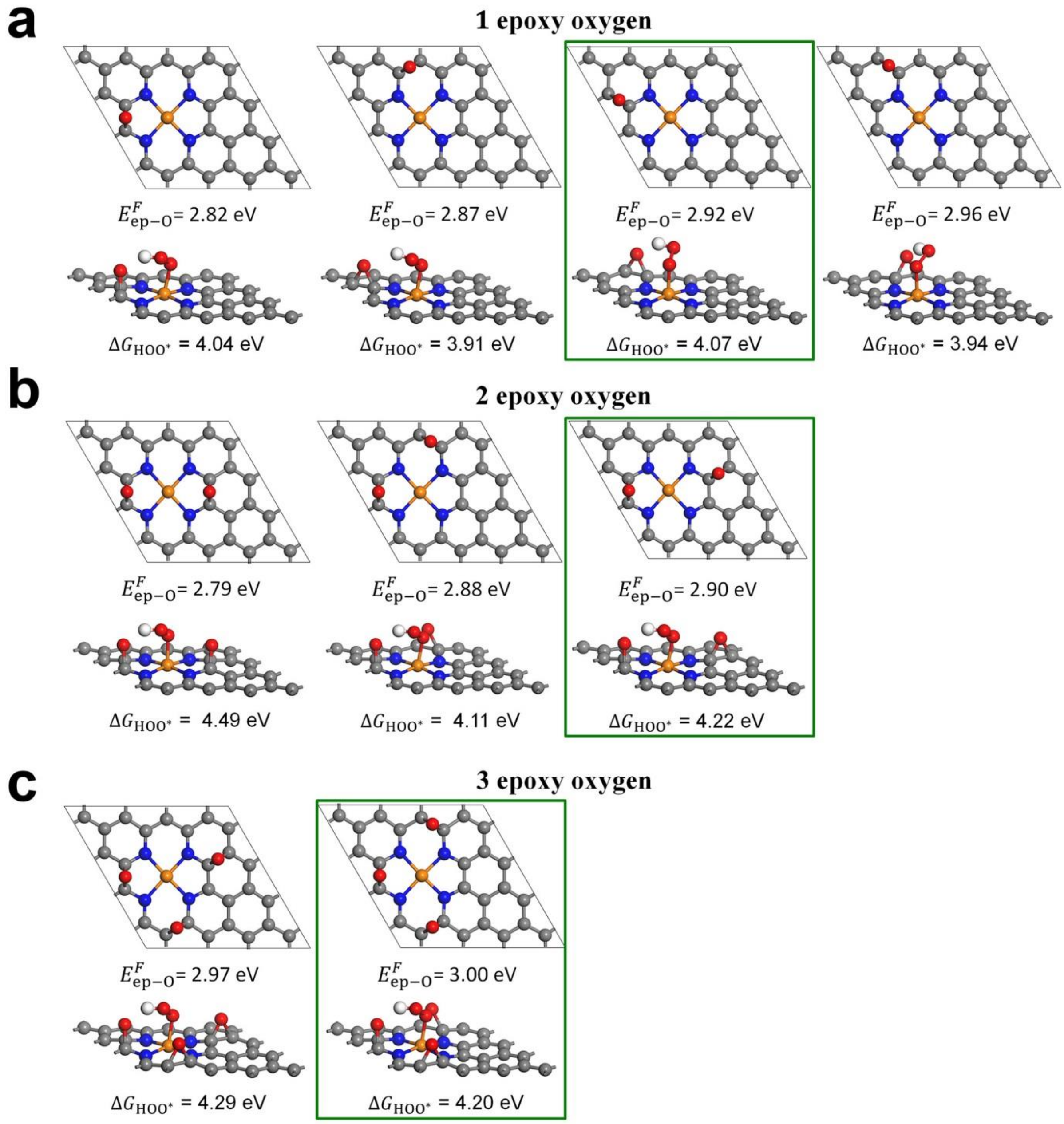

Supplementary Figure 34. The optimized geometry structures of CoN_4 catalysts with different epoxy oxygen coverages (upper) and after the adsorption of HOO* at Co site (lower). The corresponding $E^F_{\mathrm{ep-O}}$ and $\Delta G_{\mathrm{HOO^*}}$ are listed below. Here, we only show the low $E^F_{\mathrm{ep-O}}$ structures, and the green boxes in Supplementary Figure 34a, b and c indicate the structures we used to calculate the ORR activities in Fig. 4d in the main text. Specifically, the structures in Supplementary Figure 34a, b and c, respectively, are named as CoN_4 (1O), CoN_4 (2O) and CoN_4 (3O).

In electrochemical oxygen reduction reaction (ORR), the O_2 can be convert through one of two reactions: the 4-electron process to form H_2O (equation (1)) or the 2-electron process to synthesize H_2O_2 (equation (2))[7,8].

$$\mathrm{O_2 + 4H^+ + 4e^- \rightarrow 2H_2O} \qquad U_0 = 1.23\ \text{V versus RHE} \tag{S1}$$

$$\mathrm{O_2 + 2H^+ + 2e^- \rightarrow H_2O_2} \qquad U_0 = 0.70\ \text{V versus RHE} \tag{S2}$$

where U_0 is the equilibrium potential for each reaction, and RHE is the reversible hydrogen electrode. For an efficient catalyst for electroreduction of O_2 to H_2O_2, the catalyst should provide high activity, by minimizing the overpotential for the 2–electron pathway (equation (S1)), and high selectivity, by suppressing the 4–electron pathway (equation (S2))[7,8].

It is generally accepted that the 2–electron ORR to H_2O_2 involves two coupled electron and proton transfers:

$$\mathrm{O_2 + {}^* + (H^+ + e^-) \rightarrow HOO^*} \tag{S3}$$

$$\mathrm{HOO^* + (H^+ + e^-) \rightarrow H_2O_2 + {}^*} \tag{S4}$$

where * represents an unoccupied active site on electrocatalyst surface, and HOO* represents the sole adsorbed intermediate for the reaction. The $\Delta G_{\mathrm{HOO^*}}$ is proven to be the key parameter, or descriptor, to characterize the activity for the 2–electron ORR to H_2O_2, which lead to a Sabatier volcano where the highest activity is achieved on the surface with a moderate interaction with HOO*[7,8]. The limiting potential (U_{L}) is defined as the lowest potential at which the two reaction steps (equation (S3) and equation (S4)) are downhill in free energy, and the theoretical overpotential (η) is defined as the maximum difference between the U_{L} and U_0. For the ideal catalyst, the $\Delta G_{\mathrm{HOO^*}}$ should be about 4.22 eV, so that $U_{\mathrm{L}} = 0.70\ \mathrm{V}$ and $\eta = 0\ \mathrm{V}$[7,8]. Weaker binding to HOO* (right-hand side of volcano) are limited by hydrogenation of O_2 (equation (S3)), while stronger binding to HOO* (left-hand side of volcano) will lead to the overpotential due to the reduction of HOO* to H_2O_2 (equation (S4)).

For the 4-electron ORR to H_2O, we considered the four electron reaction pathway:

$$\mathrm{O_2 + * \rightarrow O_2^*} \tag{S5}$$

$$\mathrm{O_2^* + H^+ + e^- \rightarrow HOO^*} \tag{S6}$$

$$\mathrm{HOO^* + H^+ + e^- \rightarrow O^* + H_2O} \tag{S7}$$

$$\mathrm{O^* + H^+ + e^- \rightarrow OH^*} \tag{S8}$$

$$\mathrm{OH^* + H^+ + e^- \rightarrow H_2O + *} \tag{S9}$$

where $*$ represents an unoccupied active site on electrocatalyst surface, and O^*, OH^* and HOO^* are adsorbed intermediates for the reaction.

Here, we used computational hydrogen electrode (CHE) model proposed by Nørskov *et al.* to calculate the free energy levels of all intermediates[9,10]:

$$\Delta G_{\mathrm{ads}} = \Delta E_{\mathrm{ads}} + \Delta ZPE - T\Delta S + eU \quad \text{(S10)}$$

where ΔE_{ads} is the binding energy of adsorption species OH^*, O^*, and HOO^*. ΔZPE, ΔS, U and e are the ZPE changes, entropy changes, applied potential at the electrode, and charge transferred. The contributions of each component for ΔG_{ads} were obtained from previous literature[10]. As the ground state of O_2 molecule is poorly described in DFT calculations, we used gas-phase H_2O and H_2 as reference states as they are readily treated in the DFT calculations. In our simulations, the solvation effects were not considered.

To determine the energetically stability of epoxy groups at different locations of the CoN_4 catalysts, we calculated the formation energy of epoxy oxygen ($E^F_{\mathrm{ep-O}}$) on CoN_4 catalysts, which defined as $E^F_{\mathrm{ep-O}} = E(\mathrm{CoN_4} + \mathrm{O}) - E(\mathrm{CoN_4}) - [E(\mathrm{H_2O}) - E(\mathrm{H_2})]$, where $E(\mathrm{CoN_4} + \mathrm{O})$, $E(\mathrm{CoN_4})$, $E(\mathrm{H_2O})$, and $E(\mathrm{H_2})$ are the total energy of CoN_4 catalyst with epoxy oxygen, bare CoN_4 catalyst, H_2O gas, and H_2 gas, respectively. According to this definition, a less positive formation energy indicates a more energetically stable structure.

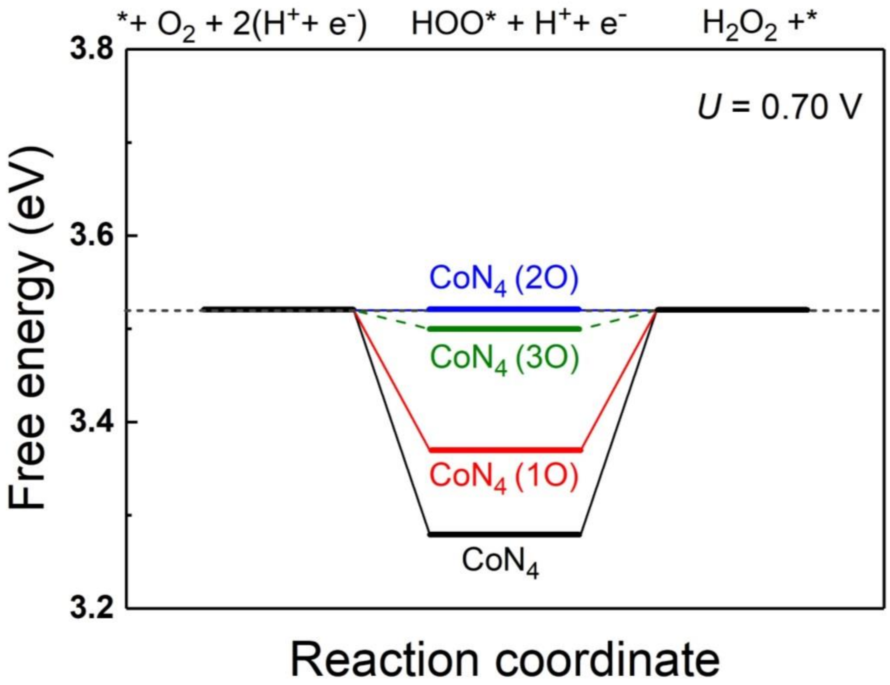

Supplementary Figure 35. Free-energy diagrams for the 2-electron ORR to H_2O_2 at the equilibrium potential ($U^0_{O_2/H_2O_2} = 0.70$ V).

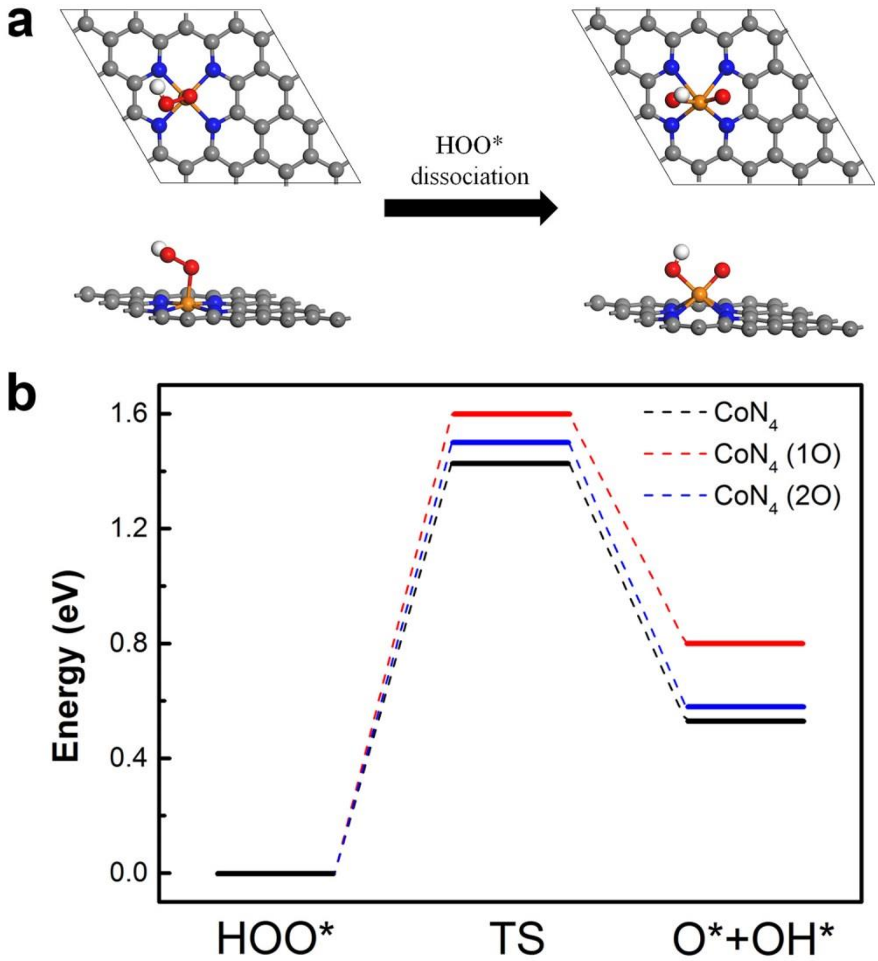

Supplementary Figure 36. (**a**) Atomistic structure of the initial state (left) and final state (right) for HOO* dissociation reaction on the CoN_4. The grey, blue, orange, red and white balls represent C, N, Co, O and H atoms, respectively. (**b**) The calculated dissociation energies of OOH* on CoN_4, CoN_4 (1O) and CoN_4 (2O).

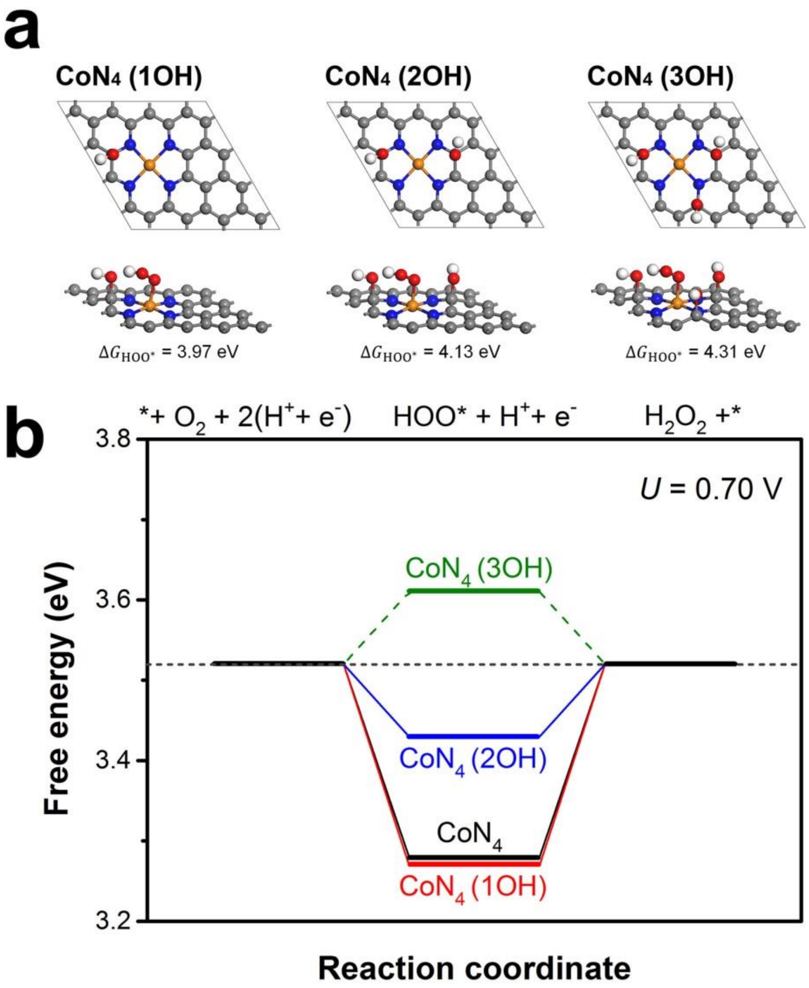

Supplementary Figure 37. (**a**) The optimized geometry structures of CoN_4 moieties with different hydroxyl oxygen coverages, which were chosen to calculate the ORR activities in this work. Here, CoN_4 (1OH), CoN_4 (2OH) and CoN_4 (3OH) indicate CoN_4 moieties with 1, 2 or 3 hydroxyl oxygen, respectively. The grey, blue, orange and red balls represent C, N, Co and O atoms, respectively. (**b**) Free-energy diagrams for the 2-electron ORR to H_2O_2 at the equilibrium potential ($U^0_{O_2/H_2O_2} = 0.70$ V).

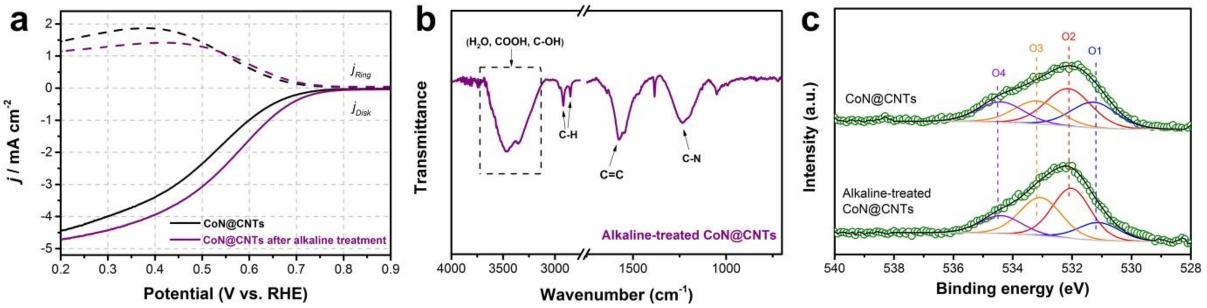

Supplementary Figure 38. (**a**) RRDE voltammograms of CoN@CNTs and alkaline-treated CoN@CNTs at 1600 rpm in an O_2-saturated 0.1 M $HClO_4$ electrolyte with disc current and ring current. (**b**) Background-corrected FTIR spectra of CoN@CNTs after alkaline treatment. (**c**) High resolution XPS O 1s spectra of CoN@CNTs before and after alkaline treatment.

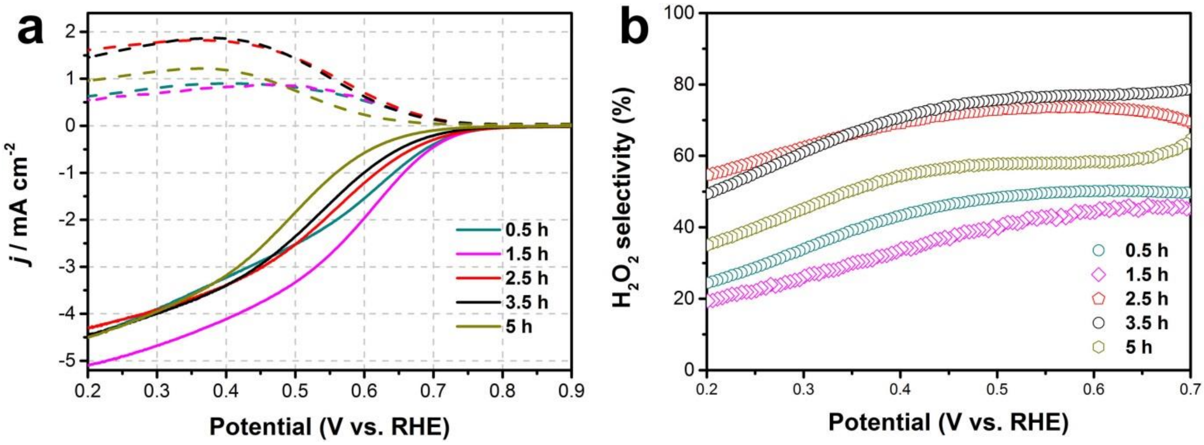

Supplementary Figure 39. (**a**) ORR polarization curves at 1600 rpm (solid lines) and simultaneous H_2O_2 detection currents at the ring electrode (dashed lines) for the CoN@CNTs prepared within different pyrolzing time (from 0.5 to 5 h). The measurements were conducted in 0.1 M $HClO_4$ solution saturated with O_2. (**b**) The calculated H_2O_2 selectivity on the CoN@CNTs samples prepared within different pyrolzing time (from 0.5 to 5 h). The sample obtained within the highest selectivity toward H_2O_2 (3.5 h) was used throughout this work.

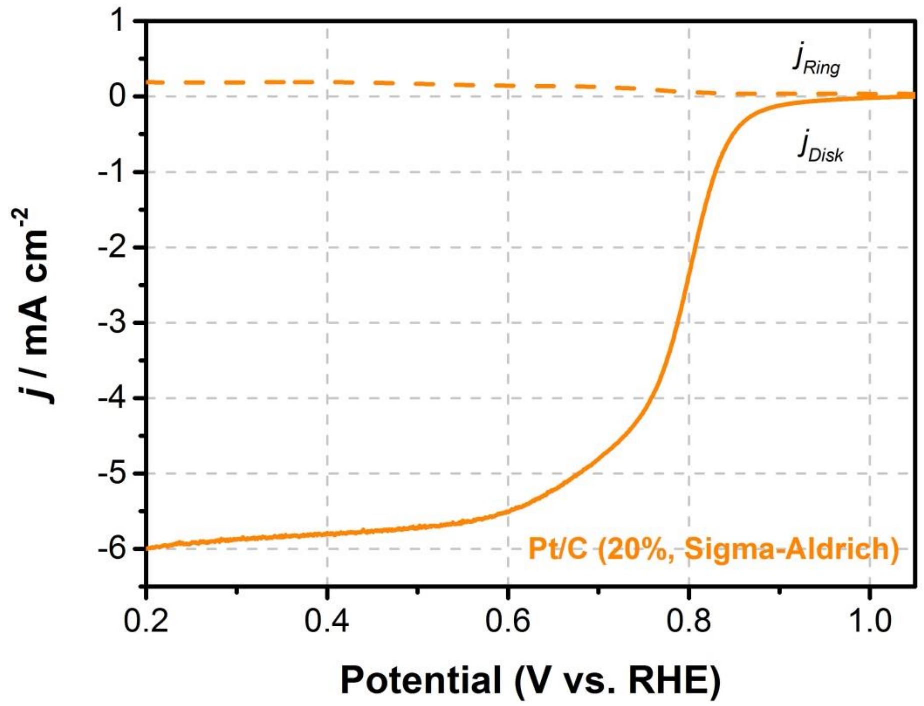

Supplementary Figure 40. ORR polarization curve of commercial Pt/C (20 wt%, Sigma-Aldrich) in O_2-saturated 0.1 M $HClO_4$ under 1600 rpm. The Pt ring is held at 1.2 V to oxidize the H_2O_2 generated from disk electrode.

Supplementary Tables:

Supplementary Table 1. EXAFS fitting parameters at Co K-edges of CoN@CNTs.

Sample	Co-N CN[a]	Co-N R (Å) [b]	σ^2 (Co-N)[c]	Co-C CN	Co-C R (Å)	σ^2 (Co-C)
Co K-edge CoN@CNTs	4.02 ± 0.36	2.114 ± 0.008	0.007 ± 0.003	5.02 ± 0.81	3.04 ± 0.04	0.007 ± 0.003

[a]CN: coordination numbers; [b]R: bond distance; [c]σ^2: Debye-Waller factors;

Supplementary Table 2. Summarization of Co, N, C and O content in the different CoN@CNTs samples based on the XPS measurements.

	Co (atom%)	N (atom%)	C (atom%)	O (atom%)
Fresh CoN@CNTs	0.69	4.29	91.32	3.50
Aged CoN@CNTs	0.45	4.18	90.20	5.17
EO-CoN@CNTs	0.66	3.50	83.18	12.66
EA-CoN@CNTs	0.35	4.37	89.80	5.48
HT-CoN@CNTs	0.66	3.32	73.68	22.34
HE-CoN@CNTs	0.48	2.76	84.41	12.35
NG	0	2.95	95.72	1.33

Supplementary Table 3. Summarization of the content of different O groups in the different CoN@CNTs samples based on the XPS measurement. The content of O2 could be ascribed to the epoxy O. The ratio means the percentage of a certain O group in the total O content, and the atom% reflects the amount of a certain O group in the whole materials.

	O1 (ratio/**at%**)	O2 (ratio/**at%**)	O3 (ratio/**at%**)	O4 (ratio/**at%**)	O5 (ratio/**at%**)	Total (**at%**)
Fresh CoN@CNTs	24.1/**0.84**	35.2/**1.23**	21.6/**0.76**	19.1/**0.67**	-	**3.50**
Aged CoN@CNTs	51.0/**2.64**	26.3/**1.36**	18.6/**0.96**	4.1/**0.21**	-	**5.17**
EO-CoN@CNTs	12.7/**1.61**	25.6/**3.24**	33.2/**4.20**	24.7/**3.13**	3.8/**0.48**	**12.66**
EA-CoN@CNTs	21.4/**1.17**	55.8/**3.06**	15.9/**0.87**	6.9/**0.38**	-	**5.48**
HT-CoN@CNTs	11.6/**2.59**	43.3/**9.67**	37.4/**8.36**	7.7/**1.72**	-	**22.34**
HE-CoN@CNTs	11.8/**1.46**	71.3/**8.81**	13.7/**1.69**	3.2/**0.39**	-	**12.35**

Supplementary Table 4. Comparison of our work and recently reported catalysts for H_2O_2 production via ORR in acid. *j* means current density.

Catalyst	Selectivity at different potentials (%@V_{RHE})	pH value	Accumulation (mg L^{-1}@V vs. RHE)	j_{ORR}@ 0.3 V vs. RHE (mA cm^{-2})	Stability (stable for a certain duration)	Mass loading (mg cm^{-2})	Reference
Pt-Hg/C	90@0.4	1	N/A	~0.74	8000 cycles	N/A	7
Pt-Hg	<96@0.2~0.4	1	N/A	~3	N/A	N/A	7
$Au_{1-x}Pd_x/C$	89.3@0.3	1	N/A	~1.4	N/A	0.19	11
Pt/TiN	54@0.4	1	N/A	~1.2	1 h	N/A	12
O-CNTs	52.5@0.1	1	N/A	0	N/A	0.1	13
Carbon-coated Pt	41@0.1	0	N/A	1.2	A decay of 10% in current for 24 h	0.08	14
Mesoporous NC	65.2@0.1	1	~20@0.1 V (5.75 h)	1.25	6 h	0.325	15
NCMK3IL50_800T	93@0.1	1	N/A	0.25	6 h	0.05	16
Au/C-35	81@0.1	1	N/A	~2.2	N/A	N/A	17
HPC-H24	91.2@0	1	7568.4@-0.2 V (2.5 h)	2.4	6 times of succesive H_2O_2 synthesis (150 min/each)	0.04	18
Vulcan XC-72R	70@0.1	1	170@0.1 V (24 h)	0	N/A	2.5	19
g-N-CNH	82@0.2	1	1@0.3 V (60 min)	N/A	24 h	15	20

Ag	87@0.2	1	N/A	2	N/A	N/A	8
Pd-Hg/C	85@0.3	1	N/A	~0.56	N/A	N/A	8
RF-AQ/VC	83@0	1	432@0.1 V (24 h)	0	N/A	2.5	19
Mn-N/C	80@0	1	N/A	N/A	N/A	N/A	21
FePc/C	78.2@-0.26	0.7	~230@-1 V (60 min)	0	N/A	N/A	22
Pt/HSC	94@0.5	1	~2.65@0V (60 min)	~0.64	6 h	0.05	23
Co_1-NG(O)	43@0.55	1	N/A	~1.8	N/A	0.01	24
Co-N-C	60@0.5	0.3	N/A	~2.7	N/A	0.1	25
**EA-CoN@CNTs**	**97.0@0.63**	**1**	**N/A**	**3**	**12 h**	**0.25**	**This work**
**HE-CoN@CNTs**	**97.5@0.50**	**1**	**1198.5@0 V (30 min) 1266.5@0.3 V (120 min)**	**2.6**	**12 h**	**0.25**	**This work**

Supplementary References:

1. Chong, L. *et al.* Ultralow-loading platinum-cobalt fuel cell catalysts derived from imidazolate frameworks. *Science* **362**, 1276-1281 (2018).
2. Goellner, V., Armel, V., Zitolo, A., Fonda, E. & Jaouen, F. Degradation by Hydrogen Peroxide of Metal-Nitrogen-Carbon Catalysts for Oxygen Reduction. *J. Electrochem. Soc.* **162,** H403–H414 (2015).
3. Cao, L. *et al.* Identification of single-atom active sites in carbon-based cobalt catalysts during electrocatalytic hydrogen evolution. *Nat. Catal.* **2**, 134-141 (2018).
4. Guo, D. *et al.* Active sites of nitrogen-doped carbon materials for oxygen reduction reaction clarified using model catalysts. *Science* **351**, 361–365 (2016).

5. Wang, X. X. *et al.* Nitrogen-Coordinated Single Cobalt Atom Catalysts for Oxygen Reduction in Proton Exchange Membrane Fuel Cells. *Adv. Mater.* **30**, 1706758 (2018).
6. Xing, T. *et al.* Observation of active sites for oxygen reduction reaction on nitrogen-doped multilayer graphene. *ACS Nano* **8,** 6856–6862 (2014).
7. Siahrostami, S. *et al.* Enabling direct H_2O_2 production through rational electrocatalyst design. *Nat. Mater.* **12**, 1137–1143 (2013).
8. Verdaguer-Casadevall, A. *et al.* Trends in the electrochemical synthesis of H_2O_2: Enhancing activity and selectivity by electrocatalytic site engineering. *Nano Lett.* **14**, 1603–1608 (2014).
9. Man, I. C. *et al.* Universality in Oxygen Evolution Electrocatalysis on Oxide Surfaces. *ChemCatChem* **3,** 1159–1165 (2011).
10. Nørskov, J. K. *et al.* Origin of the overpotential for oxygen reduction at a fuel-cell cathode. *J. Phys. Chem. B* **108,** 17886–17892 (2004).
11. Jirkovský, J. S. *et al.* Single atom hot-spots at Au-Pd nanoalloys for electrocatalytic H_2O_2 production. *J. Am. Chem. Soc.* **133,** 19432–19441 (2011).
12. Yang, S., Kim, J., Tak, Y. J., Soon, A. & Lee, H. Single-atom catalyst of platinum supported on titanium nitride for selective electrochemical reactions. *Angew. Chem. Int. Ed.* **55**, 2058–2062 (2016).
13. Lu, Z. *et al.* High-efficiency oxygen reduction to hydrogen peroxide catalysed by oxidized carbon materials. *Nat. Catal.* **1**, 156–162 (2018).
14. Choi, C. H. *et al.* Hydrogen Peroxide Synthesis via Enhanced Two – Electron Oxygen Reduction Pathway on Carbon – Coated Pt Surface. *J. Phys. Chem. C* **118,** 30063–30070 (2014).
15. Fellinger, T. P., Hasché, F., Strasser, P. & Antonietti, M. Mesoporous nitrogen-doped carbon for the electrocatalytic synthesis of hydrogen peroxide. *J. Am. Chem. Soc.* **134**, 4072–4075 (2012).
16. Sun, Y. *et al.* Efficient Electrochemical Hydrogen Peroxide Production from Molecular Oxygen on Nitrogen-Doped Mesoporous Carbon Catalysts. *ACS Catal.* **8**, 2844–2856 (2018).
17. Jirkovský, J. S., Halasa, M. & Schiffrin, D. J. Kinetics of electrocatalytic reduction of oxygen and hydrogen peroxide on dispersed gold nanoparticles. *Phys. Chem. Chem. Phys.* **12,** 8042–8052 (2010).

18. Liu, Y., Quan, X., Fan, X., Wang, H. & Chen, S. High-yield electrosynthesis of hydrogen peroxide from oxygen reduction by hierarchically porous carbon. *Angew. Chem. Int. Ed.* **54**, 6837–6841 (2015).
19. Wang, A., Bonakdarpour, A., Wilkinson, D. P. & Gyenge, E. Novel organic redox catalyst for the electroreduction of oxygen to hydrogen peroxide. *Electrochim. Acta* **66,** 222–229 (2012).
20. Iglesias, D. *et al.* N-Doped Graphitized Carbon Nanohorns as a Forefront Electrocatalyst in Highly Selective O_2 Reduction to H_2O_2. *Chem* **4,** 106–123 (2018).
21. Yamanaka, I. *et al.* Study of direct synthesis of hydrogen peroxide acid solutions at a heat-treated MnCl-porphyrin/activated carbon cathode from H_2 and O_2. *J. Phys. Chem. C* **116,** 4572–4583 (2012).
22. Silva, F. L., Reis, R. M., Barros, W. R. P., Rocha, R. S. & Lanza, M. R. V. Electrogeneration of hydrogen peroxide in gas diffusion electrodes: Application of iron (II) phthalocyanine as a modifier of carbon black. *J. Electroanal. Chem.* **722–723,** 32–37 (2014).
23. Choi, C. H. *et al.* Tuning selectivity of electrochemical reactions by atomically dispersed platinum catalyst. *Nat. Commun.* **7,** 10922 (2016).
24. Jung, E. *et al.* Atomic-level tuning of Co–N–C catalyst for high-performance electrochemical H_2O_2 production. *Nat. Mater.* (2020). doi:10.1038/s41563-019-0571-5
25. Sun, Y. *et al.* Activity–Selectivity Trends in the electrochemical production of hydrogen peroxide over single-site metal–nitrogen–carbon catalysts. *J. Am. Chem. Soc.* **141,** 12372–12381 (2019).
