# Supplementary material for: Direct insights into the role of epoxy groups on cobalt sites for acidic H2O2 production
Source: Nat Commun. 2020 Aug 21;11:4181. doi: 10.1038/s41467-020-17782-5 (PMC7442824; doi:10.1038/s41467-020-17782-5)
Supplement: Supplementary file 2 — Peer Review File [file 41467_2020_17782_MOESM2_ESM.docx]

Peer Review Information

**Journal:** Nature Communications

**Manuscript title:** Direct insights into the role of epoxy groups on cobalt sites for acidic H2O2 production

**Corresponding author name(s):** Dr Xunyu Lu

**Editorial notes:**

**Transferred manuscripts**

This manuscript has been previously reviewed at another journal that is not operating a transparent peer review scheme. This document only contains reviewer comments and rebuttal letters for versions considered at *Nature Communications*.

**Reviewer comments & decisions:**

**Reviewer comments, first version:**

Reviewer #1 (Remarks to the Author):

This reviewer has argued previously that the paper by Zhang, et al. on the ORR electrocatalytic production of H2O2 by epoxy enhanced dispersed CoN4 sites in CNTs deserves publication in a Nature journal. While the catalyst itself is likely somewhat complex and inhomogeneous, the authors have done all humanely possible to characterize it morphologically, spectroscopically, electrochemically and theoretically, and their arguments are mostly convincing. In addition, this catalyst is probably the best known catalyst for H2O2 production in non-alkaline solutions and therefore could evolve into a technically important H2O2 catalyst. The authors have added significantly to the paper’s original submission by addressing the concerns of the reviewers, so that in my view it is acceptable for publication in Nat Comm in its present form.


Reviewer #2 (Remarks to the Author):

I enjoyed reading the comments from previous reviewers and additional results/discussion from the authors. I agree with most comments from the reviewers, which seems to be responded well in the rebuttal EXCEPT reviewer #3’s comment. The reviewer #3 suggested that authors needed to consider the previous two reports, but his/her comments were fully ignored. After careful reading of the two papers, I also agree the reviewer #3’s comment, many similar points highlighted in the present works with the conclusion made in the previous reports. As noted by the authors, although the depth and purpose of the works are different, I do not think that the earlier works fail to give any lessons. Comparison and discussion with the previous results will provide many interesting stories for future readers.
Furthermore, honestly…the performance of the catalysts in the device system is not such surprising. As cited in Ref. 54, the production of 20 % H2O2 was already achieved. Even though the ref. 54 is about to the systematic study (rather than catalytic materials), authors need to compare the production rates reported in 10.1038/ncomms10922 (similar device setup).
One question. I guess the authors used a GC electrode of 4 mm diameter (0.126 cm2) and the ORR was examined at 1600 rpm. If so (is it right?), the four-electron ORR should lead to diffusion current density of ca. 5~6 mA cm-2. In other words, diffusion current density from the selective two-electron ORR should be ca. 2.5~3 mA cm-2. They are theoretical values and good indicators calculating the number of electrons transferred. Thus, all the data should be transferred from current to current density. For comparison of the diffusion current densities, ORR polarization of Pt electrode should be provided. I think, then, the diffusion current density of the sample will be > 3 (I calculated…), not 100% H2O2 selectivity.


Reviewer #3 (Remarks to the Author):

The authors present Co-N4 single-atom catalysts (SACs) as selective materials to catalyse the electrochemical production of hydrogen peroxide in acidic media. They combine electrochemical characterisation with ex situ structural and chemical investigation of their catalysts and theoretical calculations. After all the detailed and relevant insights from the previous reviewers, I would like to add that I have two main concerns regarding this manuscript: novelty and stability, as explained in the points below. In summary, I cannot recommend this manuscript for publication in Nature Communications, given the reasons below:

i) The work is definitely not novel, as this kind of isolated sites have been previously presented for the selective reduction of oxygen to H2O2 by different groups. Apart from the articles mentioned by reviewer 3, Jung et al. have very recently published a manuscript in Nature Materials (https://doi.org/10.1038/s41563-019-0571-5) on Co-N4 SACs for H2O2 production as well, both in alkaline and acidic media. Interestingly, they also combine electrochemistry with detailed structural and chemical characterisation as well as theoretical calculations, leading to similar volcano-type relationships with Co-N4 catalysts at the very top of the volcano. Given the amount of work published on SACs for this reaction, before submitting this work to a more specialised journal in e.g. physical chemistry or electrochemistry, the authors would need to compare their catalysts with the state of the art, with a special emphasis in the work that has been carried out in acidic media. For instance, I am missing a summary of the activity and stability of catalysts for this reaction as represented by e.g. Tafel plots. The results need to include a discussion of these catalyst materials compared to all the other relevant work carried out on the same type of materials for this very same reaction, and emphasise what is really the new insight from this work from in situ studies.

ii) As I understood it, one interesting idea is to use this kind of sites for H2O2 production in acidic media, which I definitely agree would be key. In this regard, this work lacks a more systematic understanding of the stability of these catalysts in acidic media. Although the authors present some characterisation after the electrochemical tests, they would need to include the spectroscopic studies (e.g. X-ray absorption and infrared spectroscopy) in situ/in operando for the conclusions to be relevant. Ex-situ studies are not enough, as the structure and chemical composition of the catalysts can definitely change, and most likely will. Apart from the in situ spectroscopic studies, I miss a more systematic study on the stability and the reasons why the catalyst seem to be stable, as this is not straightforward and one would expect that a Co-based material would not be stable under these acidic conditions.

**Author rebuttal, first version:**

Referee 1

Comments to the Author:

This reviewer has argued previously that the paper by Zhang, et al. on the ORR electrocatalytic production of H_2_O_2_ by epoxy enhanced dispersed CoN_4_ sites in CNTs deserves publication in a Nature journal. While the catalyst itself is likely somewhat complex and inhomogeneous, the authors have done all humanely possible to characterize it morphologically, spectroscopically, electrochemically and theoretically, and their arguments are mostly convincing. In addition, this catalyst is probably the best known catalyst for H_2_O_2_ production in non-alkaline solutions and therefore could evolve into a technically important H_2_O_2_ catalyst. The authors have added significantly to the paper’s original submission by addressing the concerns of the reviewers, so that in my view it is acceptable for publication in Nat Comm in its present form.

**Response:** We thank the comments from this reviewer.

Referee 2:

Comments to the Author:

I enjoyed reading the comments from previous reviewers and additional results/discussion from the authors. I agree with most comments from the reviewers, which seems to be responded well in the rebuttal EXCEPT reviewer #3’s comment. The reviewer #3 suggested that authors needed to consider the previous two reports, but his/her comments were fully ignored. After careful reading of the two papers, I also agree the reviewer #3’s comment, many similar points highlighted in the present works with the conclusion made in the previous reports. As noted by the authors, although the depth and purpose of the works are different, I do not think that the earlier works fail to give any lessons. Comparison and discussion with the previous results will provide many interesting stories for future readers.

Furthermore, honestly…the performance of the catalysts in the device system is not such surprising. As cited in Ref. 54, the production of 20 % H2O2 was already achieved. Even though the ref. 54 is about to the systematic study (rather than catalytic materials), authors need to compare the production rates reported in 10.1038/ncomms10922 (similar device setup).

One question. I guess the authors used a GC electrode of 4 mm diameter (0.126 cm2) and the ORR was examined at 1600 rpm. If so (is it right?), the four-electron ORR should lead to diffusion current density of ca. 5~6 mA cm-2. In other words, diffusion current density from the selective two-electron ORR should be ca. 2.5~3 mA cm-2. They are theoretical values and good indicators calculating the number of electrons transferred. Thus, all the data should be transferred from current to current density. For comparison of the diffusion current densities, ORR polarization of Pt electrode should be provided. I think, then, the diffusion current density of the sample will be > 3 (I calculated…), not 100% H2O2 selectivity.

**Response:** We appreciate the suggestions from this reviewer. We have compared and discussed our work with two previous reports, and the following conclusions can be obtained:

(1) Jaouen and coworkers (*Energy* *Environ. Sci.*, **2018**, *11*, 3176-3182) investigated systematically the degradation mechanism of a representative Fe-N-C catalyst in acidic media during ORR. It is found that the surface oxidation of carbon caused by the reactive oxygen species is responsible for the decreased turn over frequency on FeN_x_C_y_ sites, reflected by an increased H_2_O_2_ selectivity (up to 30% in acid);
(2) Interestingly, Choi et al (DOI: 10.1016/j.cattod.2019.05.034) also reported a similar phenomenon on MOF-derived CoNC, MnNC and FeNC composites, revealing a significant increase of H_2_O_2_ selectivity (up to 80% for CoNC) after surface oxidation of carbon. Hence, the combined findings above suggest that the surface oxidation is mainly responsible for the changes of H_2_O_2_ selectivity on MNC catalysts;
(3) However, in these reports, the specific role of a certain type of oxygen functionality in affecting the H_2_O_2_ productivity is not studied. As shown in our manuscript, different oxygen groups tend to affect the H_2_O_2_ productivity in distinctive ways. For instance, ketonic groups (C=O) will suppress the H_2_O_2_ generation while epoxy groups play an advocating role, which thusly highlight the necessity of carrying out such study;
(4) With the aid of a suite of characterizations (including electrochemical and spectroscopic), we further confirm the role of epoxy groups in enhancing the H_2_O_2_ selectivity (~95 % after modification). This finding is **complementary** to these previous reports and provides a useful guideline for the future development of cost effective and active catalyst materials to produce H_2_O_2_ electrochemically.

The above discussion has been included into the revised manuscript (page 18, line 436), giving more insight and in-depth understanding into the influence of carbon oxidation toward H_2_O_2_ selectivity.

The high production rate mentioned by this reviewer (*Science*, **2019**, *366*, 226-231) is achieved with a fuel cell setup using solid electrolytes, which sheds light on the further productivity enhancement of our catalyst if such a setup could be employed. It has to be noted that the apparent performance of devices depends greatly on many factors, such as ohmic losses, flow rate, gas diffusion efficiency and cell configurations, which are equally or in some cases even more important than the catalysts themselves (*Nat. Rev. Chem.*, **2019**, *3*, 442–458; *ACS Cataly.*, **2018**, *8*, 4064-4081). The current work is mainly emphasizing on the catalyst design and investigation of the origin of H_2_O_2_ selectivity on Co-N-C materials, where RRDE is employed as the main technique for the performance assessment. Further improvement of the productivity of our catalyst could be readily achieved by using different cell setups. For example, within a gas diffusion electrode setup (GDE, **Supplementary Figure 30,** ORR at the cathode and OER at the anode), ~1200 ppm of H_2_O_2_ can be obtained within 30 min at 0 V_RHE_, which is one of the highest among all reports using similar devices in **acidic** media (**Supplementary Table 4**). Notably, the work mentioned by the reviewer in *Nat. Commun.*, **2016**, *7*, 10922 is also added into the comparison (SI, **Supplementary Table 4**), reporting a S-coordinated Pt sing-atom (noble metal) catalyst with a good H_2_O_2_ selectivity while its apparent activity (~1.4 mA cm^-2^ at 0.2 V_RHE_) is significantly lower than ours with cost-effective metal (~2.9 mA cm^-2^ at 0.2 V_RHE_ for HE-CoN@CNTs) using the same setup, giving a low H_2_O_2_ productivity (~2.65 ppm at 0 V_RHE_ for 1 h) in this report.

According to the comment, all presented data based on RRDE measurements are converted from current (*i*) to current density (*j*). It is true that the theoretical mass transport limit for the two-electron reduction of O_2_ is around 3 mA cm^-2^ under 1600 rpm. Hence, an obviously large *j* recorded on CoN@CNTs (4.4 mA cm^-2^ at 0.2 V_RHE_) is ascribed to a combined 2e^-^ and 4e^-^ ORR process, resulting in a relatively low H_2_O_2_ selectivity. In contrast, after electrochemical treatments, EA-CoN@CNTs exhibits a much receded current density (almost <3 mA cm^-2^) and an improved H_2_O_2_ selectivity (~90%), suggesting a 2e^-^-dominated ORR pathway. The slightly larger *j* (3.02 mA cm^-2^) at 0.3 V_RHE_ than the theoretical limit is due to the relatively low content of epoxy groups on EA-CoN@CNTs, giving a small amount of electrons that are consumed to form water (a H_2_O_2_ selectivity around 90%). Notably, after a combined epoxy-modification, a high coverage of epoxy groups is achieved on HE-CoN@CNTs, leading to a current density (*j*) below 3 mA cm^-2^ within the whole potential range (~2.9 mA cm^-2^ at 0.2 V_RHE_ with a surface area of ~0.126 cm^2^, **Figure 4a**) and indicating a genuine 2e^-^ ORR process where a H_2_O_2_ selectivity close to 100% is obtained. For comparison, the polarization curve of Pt/C (20%, Sigma) is adopted (SI, **Supplementary** **Figure 40** and **R1**) according to the comment, which is a superior catalyst for ORR to water, rather than H_2_O_2_.

**Figure R1.** ORR polarization curve of commercial Pt/C (20%, Sigma) in O_2_-saturated 0.1 M HClO_4_ under 1600 rpm. The Pt ring is held at 1.2 V to oxidize the H_2_O_2_ generated from disk electrode.

Referee 3:

Comments to the Author:

The authors present Co-N4 single-atom catalysts (SACs) as selective materials to catalyse the electrochemical production of hydrogen peroxide in acidic media. They combine electrochemical characterisation with ex situ structural and chemical investigation of their catalysts and theoretical calculations. After all the detailed and relevant insights from the previous reviewers, I would like to add that I have two main concerns regarding this manuscript: novelty and stability, as explained in the points below. In summary, I cannot recommend this manuscript for publication in Nature Communications, given the reasons below:

**Response:** Thanks for the comments. The concern over stability has been thoroughly addressed as shown below. Moreover, after reading the very recent work, we strongly believe that our work is novel and will have a high impact to the community due to the significant differences in the focus. The details are shown as below.

i) The work is definitely not novel, as this kind of isolated sites have been previously presented for the selective reduction of oxygen to H2O2 by different groups. Apart from the articles mentioned by reviewer 3, Jung et al. have very recently published a manuscript in Nature Materials (https://doi.org/10.1038/s41563-019-0571-5) on Co-N4 SACs for H2O2 production as well, both in alkaline and acidic media. Interestingly, they also combine electrochemistry with detailed structural and chemical characterisation as well as theoretical calculations, leading to similar volcano-type relationships with Co-N4 catalysts at the very top of the volcano. Given the amount of work published on SACs for this reaction, before submitting this work to a more specialised journal in e.g. physical chemistry or electrochemistry, the authors would need to compare their catalysts with the state of the art, with a special emphasis in the work that has been carried out in acidic media. For instance, I am missing a summary of the activity and stability of catalysts for this reaction as represented by e.g. Tafel plots. The results need to include a discussion of these catalyst materials compared to all the other relevant work carried out on the same type of materials for this very same reaction, and emphasise what is really the new insight from this work from in situ studies.

**Response:** Single-atom catalysts (SACs) have been widely used for electrocatalytic reactions and appear to be promising alternatives to the precious metals in many applications. However, for oxygen reduction reaction (ORR), there is a long-held puzzle that why the Co-N_4_-C catalysts prepared within similar methods exhibit different H_2_O_2_ selectivity and ORR activity, especially for those in acid, making the reaction mechanism under strong debate. Moreover, the activity of the previous reports, including the very recent Nature Materials work (~1.7 mA cm^-2^ at 0.2 V_RHE_ in 0.1 M HClO_4_ with a H_2_O_2_ selectivity below 50%), still call for further improvement in **acid** to meet the requirement of those in industry. Hence, in-depth studies are required to investigate the real origin of H_2_O_2_ selectivity on Co-N-C materials to further enhance their catalytic performance in acid.

The Nature Materials work (https://doi.org/10.1038/s41563-019-0571-5) mentioned by this reviewer presents an interesting method in reducing the oxygen content on the Co-N_4_ embedded graphene via thermal reduction, concluding the significance of synergies between Co and O species for achieving a high H_2_O_2_ selectivity in **alkaline**. Nevertheless, similar to the two previous reports (*Adv. Mater.*, **2019**, *31*, 1808173 and https://doi.org/10.1016/j.cattod.2019.05.034), no direct correlations between specific functional groups and H_2_O_2_ selectivity is established. Thus, detailed **experimental studies** are required to systematically point out the effective oxygen species in determine the H_2_O_2_ selectivity on Co-N_4_ sites, which is answered to the best as we can in our manuscript by precisely controlling the modification of epoxy groups.

Moreover, this manuscript was submitted at a similar time (16^th^ June 2019) with the Nature Materials work, and we are more than excited to know that the peer researchers conducted a concurrent work with us to push forward the research in this field. Thanks for these reports, we have a chance to deepen the understanding in this area by combining different important findings together (actually complementary to each other to some extent), and I believe this is the reason why we’re doing research.

According to reviewer’s comments, additional comparisons with the state-of-the-art catalysts in acidic media is given in the updated manuscript (**Figure 4d** and **Supplementary Table 4**), including the very recent published papers. As suggested, a summary of the activity and stability of catalysts for H_2_O_2_ production in acid is also added into the manuscript. The Tafel slopes of CoN@CNTs and other comparison samples are presented in **Supplementary Figure 8**, while the comparison between our catalyst and other reported composites based on Tafel plots cannot be carried out due to the lack of authorized/accurate experimental data presented in others’ works. Alternatively, comparisons based on apparent H_2_O_2_ productivity, such as selectivity (%), current density (mA cm^-2^), overpotential (mV) and H_2_O_2_ production rate (mg L^-1^), is more informative than Tafel plots since the performance under practical operation conditions can be well reflected by these parameters.

ii) As I understood it, one interesting idea is to use this kind of sites for H2O2 production in acidic media, which I definitely agree would be key. In this regard, this work lacks a more systematic understanding of the stability of these catalysts in acidic media. Although the authors present some characterisation after the electrochemical tests, they would need to include the spectroscopic studies (e.g. X-ray absorption and infrared spectroscopy) in situ/in operando for the conclusions to be relevant. Ex-situ studies are not enough, as the structure and chemical composition of the catalysts can definitely change, and most likely will. Apart from the in situ spectroscopic studies, I miss a more systematic study on the stability and the reasons why the catalyst seem to be stable, as this is not straightforward and one would expect that a Co-based material would not be stable under these acidic conditions.

**Response:** Thanks for the comments. Metallic Co and cobalt oxides are not stable in acid as they will dissolve swiftly. However, it is well established that nitrogen coordinated Co atoms that are embedded within carbon substrates (Co-N-C) are highly stable in acidic media. This type of catalysts has been employed widely for ORR in acids (*Science*, **2018**, *362*, 1276-1281; *Nat. Commun.*, **2017**, *8*, 957; *Nat. Catal.*, **2018**, *1*, 935-945; *Adv. Mater.*, **2018**, *30*, 1706758; *Energy Environ. Sci.*, **2019**, *12*, 250-260) without any stability issues observed. Moreover, *in situ* study on the Co-N-C catalysts for ORR has already been carried out by many previous works (*Nat. Commun.*, **2017**, *8*, 957; *Proc. Natl. Acad. Sci. USA*, **2018**, *115*, 12692-12697), showing a stable property of CoN_4_ moieties under the reaction conditions. Incremental work should not be carried out to reveal the same information again. Therefore, we don’t see the necessity of carrying out another *in-situ* study to confirm the information that has been long-proven.

Apparently, the stability of our catalyst in acidic media during H_2_O_2_ production has already been studied systematically through a series of physical and chemical characterizations. Firstly, post-reaction XPS results (**Supplementary Figure 27**) show that no detectable changes of Co, N and O elements were observed on the HE-CoN@CNTs after long-term H_2_O_2_ production, suggesting its superior chemical stability. Then, TEM images (**Figure R2**) confirm the robust stability of the structural features of our catalyst, revealing a similar CNTs morphology before and after reaction. FTIR measurements (**Supplementary Figure 28**) conducted after reaction show that the epoxy groups are well retained on the HE-CoN@CNTs, further proving the stable chemical property of the active sites. Moreover, the catalysts are not only stable under the long-term operation conditions, but also under the electrochemical/chemical treatments (**Supplementary Figure 19**, **20** and **25**). ICP-MS results prove that no leaching of Co elements was observed even under the electrochemical oxidation condition, further corroborating the stability of the CoN_4_ active sites. Typically, the stability of catalysts mainly depends on the chemical status of active sites and the robustness of catalysts’ physical structure. Hence, the above results provide solid evidences to confirm the stability of our catalysts for H_2_O_2_ production in acids.

**Figure R2.** TEM images of HE-CoN@CNTs after 12-h H_2_O_2_ production via ORR in O_2_-saturated 0.1 M HClO_4_.

**Figure R3.** The chronoamperometric curve of HE-CoN@CNTs loaded GDE obtained at 0.3 V vs. RHE for H_2_O_2_ production after long-term H_2_O_2_ production sessions (shown in Supplementary Figure 31A and B) in the refreshed electrolyte (surface area = 9 cm^2^).

Further, no significant decay of activity is observed on our catalyst in not only a H-cell setup but also the gas diffusion electrode (GDE) device during a 12-h stability test (**Figure 3g** and **Supplementary Figure 31**), demonstrating a superior stability of catalytic activity. Even though a slight decrease of current was observed in the GDE after 12h of reaction, this phenomenon is mainly caused by the adsorption of peroxide species or metal cations on the surface of catalysts. The activity can be easily recovered by cleaning the electrode surface and changing the electrolyte (**Supplementary Figure 31**), which can be used for another 12 hours of reaction with stable current (**Figure R3**).

The collective results have proved that the as-prepared catalyst is chemically and physically stable for H_2_O_2_ production in acid.

**Reviewer comments, second version:**

Reviewer #2 (Remarks to the Author):

Most concerns raised by this reviewer were well responded. I recommend publishing this work in Nature Communications, AFTER one MINOR correction on ORR activity measurement. ORR polarization curve of commercial Pt/C catalyst, newly added in the revised manuscript, shows the very poor activity as compared to that reported in other groups. Note that the inferior activity may result from unexpected impurities in the electrolyte, artifacts induced by potential-scan direction, or many other experimental parameters. Hence, I recommend the authors to measure the ORR activity of Pt/C again and to check whether it fits well with other results reported in the literature.

**Author rebuttal, second version:**

Referee 2:

Comments to the Author:

Most concerns raised by this reviewer were well responded. I recommend publishing this work in Nature Communications, AFTER one MINOR correction on ORR activity measurement. ORR polarization curve of commercial Pt/C catalyst, newly added in the revised manuscript, shows the very poor activity as compared to that reported in other groups. Note that the inferior activity may result from unexpected impurities in the electrolyte, artifacts induced by potential-scan direction, or many other experimental parameters. Hence, I recommend the authors to measure the ORR activity of Pt/C again and to check whether it fits well with other results reported in the literature.

**Response:** We thank the suggestions given by this reviewer. It is true that the electrocatalytic performances of Pt/C tend to be affected by many factors, including but not limiting to, reaction media, catalyst loading, carbon support, system setup and impurities in the electrolytes. Therefore, the ORR performances of the same Pt/C catalyst reported by different groups always vary.

To re-testify the catalytic ORR performances of Pt/C catalyst, commercially available Pt/C composites obtained from two different suppliers (Sigma-Aldrich and Premetek, 20 wt% of Pt) were evaluated and compared in O_2_-saturated 0.1 M HClO_4_ (**Figure R1**). During the electrochemical measurements, a rotating disk electrode (RDE, 5 mm, ALS Japan) mounted with Pt/C catalyst (0.25 mg cm^-2^) was employed as the working electrode under a rotating speed of 1600 rpm. A graphite rode and saturated calomel electrode (SCE) were used as counter and reference electrode, respectively. Before testing, the SCE reference electrode was calibrated carefully using the hydrogen oxidation method (*ACS Energy Lett.* **2020**, *5*, 1083−1087), thus giving the reliability of the reported potentials that have been converted to values referred to reversible hydrogen electrode (RHE). As shown in **Figure R1**, the ORR activities on two different Pt/C catalysts both exhibit an onset potential (*E*_onset_) and half-wave potential (*E*_1/2_) of around 0.94 and 0.78 V, respectively, which are very similar to those reported in literature (*Chem* **2018**, *4*, 2345-2356; *Nat. Commun.* **2017**, *8*, 15938; *J. Am. Chem. Soc.* **2017**, *139*, 17269-17272; *Nano Energy* **2017**, *37*, 187-194; *ACS Catal.* **2015**, *5*, 1857-1862; *J. Am. Chem. Soc.* **2013**, *135*, 7130-7133), suggesting the authenticity of ORR activities obtained over our Pt/C catalysts in acidic environment. To note, the slight variations in *E*_1/2_ (±20 mV) between our results and those reported in literature can be ascribed to the differences in testing conditions, as mentioned previously. The re-evaluated ORR performance of Pt/C (Sigma-Aldrich) has been added into the manuscript accordingly.

**Figure R1.** ORR polarization curves of commercial Pt/C (20 wt%) obtained from different companies in O_2_-saturated 0.1 M HClO_4_ solution under 1600 rpm using a RDE (diameter: 5 mm, ALS Japan) setup without *i*R correction.

**Reviewer comments, third version:**

Reviewer #2 (Remarks to the Author):

All the concerns raised by this reviewer were addressed well. Now, I recommend accepting the manuscript for publication in this journal.

**Author rebuttal, third version:**
